# Prevalence and associated risk factors of *Pseudomonas aeruginosa* infection in Diabetic wounds in Duhok province, Iraq

**DOI:** 10.1371/journal.pone.0354256

**Published:** 2026-07-24

**Authors:** Marwa S. Ibrahim, Ibrahim A. Naqid

**Affiliations:** 1 Department of Medical Laboratory Technology, College of Health and Medical Technology-Shekhan, Duhok Polytechnic University, Duhok, Kurdistan Region, Iraq; 2 Department of Biomedical Sciences, College of Medicine, University of Zakho, Duhok, Kurdistan Region, Iraq; The University of Jordan, JORDAN

## Abstract

**Background and aims:**

Diabetes mellitus is a chronic metabolic disorder and a major global health problem, predisposing patients to wound infections, with *Pseudomonas aeruginosa* being a frequent and resistant pathogen. This study aimed to identify *P. aeruginosa*, assess associated risk factors, and determine its antibiotic susceptibility pattern in diabetic wound infections in Duhok Province, Kurdistan Region, Iraq.

**Methods:**

This cross-sectional study was conducted from June 2024 to July 2025 in Duhok Province, Kurdistan Region, Iraq. A total of 141 wound swab samples were collected from diabetic patients aged ≥18 years. *P. aeruginosa* was identified using biochemical tests, Vitek®2, and 16S rDNA PCR. Antimicrobial susceptibility testing was performed by the disc diffusion method on Mueller–Hinton agar, and data were analyzed using GraphPad Prism software.

**Results:**

Out of 141 wound infection samples collected from diabetic patients, *P. aeruginosa* was isolated in 44.68% of cases. Overall, sociodemographic factors showed no significant association with PsA infection, except for occupational status in the multivariable analysis 0.12 [0.02–0.97, p = 0.046] and overweight 0.29 [0.10–0.82, p = 0.019]. Clinically, most variables including HbA1c, duration of diabetes, comorbidities, treatment type, and wound characteristics were not significantly associated with PsA infection. The only significant predictor was wound location, with leg-region infections showing a higher risk of PsA 3.94 [1.24–12.5, p = 0.02]. *P. aeruginosa* isolates showed high levels of multidrug resistance, especially to aztreonam, ceftazidime, cefepime, and tobramycin. Colistin showed the lowest resistance and highest sensitivity, followed by relatively better susceptibility to carbapenems.

**Conclusion:**

*P. aeruginosa* is a common cause of wound infections in patients with diabetes mellitus, with its prevalence significantly associated with body mass index, occupational status and wound location, with leg-region wounds showing a higher risk. *P. aeruginosa* isolates demonstrated high multidrug resistance, although colistin and carbapenems retained relatively better activity. Early detection and appropriate antibiotic selection are essential to reduce complications and improve patient outcomes.

## Introduction

Diabetes mellitus (DM) is a chronic metabolic disorder characterized by persistent hyperglycemia and disturbances in carbohydrate and lipid metabolism, resulting in insulin resistance [1]. It is recognized as a major global public health problem and an emerging epidemic, contributing significantly to morbidity and disability in both developing and developed countries [1,2]. DM is classified into type 1 and type 2 diabetes [3]. Patients with type 2 diabetes are at increased risk of developing multiple complications, including hypertension, cardiovascular disease, renal failure, cerebrovascular accidents, retinopathy, and neuropathy [4,5]. In addition to comorbidities, several factors influence the risk and severity of infection. These include local factors such as poor tissue perfusion, presence of foreign bodies, necrosis, wound location and size, and poorly controlled diabetes. Behavioral factors such as malnutrition, non-adherence to treatment plans, and smoking, as well as social determinants such as educational level and socioeconomic status, also play important roles [6].

Furthermore, peripheral ischemic disease is a frequent and severe complication that can lead to ulceration and wound infection, particularly diabetic foot ulcers (DFU) [1]. Ulceration is the most common clinical manifestation of wound infections, followed by cellulitis and gangrene. The dorsum of the foot is the most commonly affected site, followed by the forefoot and toes [7]. This condition may further progress to osteomyelitis [8]. Among DM patients who develop foot ulcers, approximately 20–30% require minor amputation, while about 5% undergo major amputation [6].

Chronic wound infections are typically polymicrobial, and microbial colonization of a localized wound can lead to extensive replication on the wound surface. As the microbial load increases, pathogens may penetrate deeper tissues, resulting in clinical signs and symptoms in diabetic patients [6]. Both Gram-positive and Gram-negative bacteria can cause wound infections, with *P. aeruginosa* being one of the most common pathogens in diabetic patients [9,10]. This organism can cause severe infections in multiple sites, including pneumonia, urinary tract infections, keratitis, and soft tissue infections of open wounds, particularly diabetic foot ulcers and burn injuries [11]. It produces multiple toxins, evades phagocytosis, and exhibits broad antibiotic resistance, making it a highly virulent and difficult-to-treat pathogen [2]. A previous study in Erbil, Iraq reported a prevalence of *P. aeruginosa* in diabetic foot infections of 35.33% [11]. Another study conducted in Erbil [12] found *P. aeruginosa* to be the most common Gram-negative bacterium, accounting for 21.43% of isolates. In contrast, a study in Sulaymaniyah reported a lower prevalence of *P. aeruginosa* among diabetic foot infections (8.5%) [13].

Antibiotic resistance in *P. aeruginosa* has become a major global public health concern, particularly due to the limited availability of effective alternative treatment. Infections caused by this pathogen significantly contribute to morbidity and mortality worldwide. According to the European Centre for Disease Prevention and Control, 13.7% of *P. aeruginosa* isolates are resistant to at least three antimicrobial groups, while 5.5% are resistant to all five groups under surveillance. This highlights the growing challenge of multidrug resistance and the need for effective antibiotic stewardship and infection control measures [14]. The declining sensitivity of *P. aeruginosa* to commonly used antibiotics underscores the urgent need to prevent antibiotic overuse and to strengthen antimicrobial stewardship efforts, which may help curb resistance in the future [10]. Furthermore, antibiotic selection for the treating aeruginosa infections is often inconsistent, highlighting the critical importance of choosing appropriate, evidence-based therapeutic regimens [11].

Most previous studies in the Kurdistan Region of Iraq have focused mainly on *P. aeruginosa* in diabetic foot ulcers. Therefore, the present study aimed to isolate P. aeruginosa from wound infections at various anatomical sites among diabetic patients in different hospitals in Duhok Province, Kurdistan Region, Iraq. In addition, it aimed to identify key risk factors associated with *P. aeruginosa* wound infections and determine the antimicrobial susceptibility patterns of the isolates.

## Materials and methods

### Study design, and sample collection

This cross-sectional study was conducted in Duhok Province, Kurdistan Region, Iraq, from July 2024 to July 2025. A total of 141 wound samples were collected from diabetic outpatients (77 females, 64 males) aged ≥18 years at Zakho General Hospital and Azadi Teaching Hospital using a **convenience sampling** approach, based on the availability of eligible patients during the study period. The sample size of 141 was based on all eligible diabetic wound infection cases collected during the study period in selected hospitals; therefore, a census sampling approach was applied. Wounds were first cleaned with normal saline, after which samples were obtained using sterile swabs and transported in Amies medium. All samples were cultured on appropriate media at the Microbiology Laboratory of Zakho Technical Institute. Written informed consent was obtained from all participants before sampling.

### Severity of wound infection

The severity of wound infection was classified based on the degree of tissue involvement. The severity was defined based on the degree of tissue involvement and clinical presentation, including ulcers (necrosis with erythema, pain, and discharge), abscess formation (pus collection), cellular necrosis (tissue death due to hypoxia or vascular occlusion), and gangrene (advanced necrosis with or without systemic symptoms). Poor skin care and unrecognized skin injuries may progress to chronic ulcers, facilitating microbial invasion of skin tissue. As infection extends to deeper layers, it may involve muscles, joints, and tendon sheaths, leading to pus accumulation, tissue necrosis, and abscess formation. Diabetic ulcers are open skin wounds characterized by necrosis, surrounding erythema, pain, and purulent discharge [15]. Necrosis may also result from vascular occlusion or trauma, leading to tissue hypoxia and cell death. Advanced necrosis can results in eschar formation and may progress to wet or dry gangrene, often accompanied by systemic symptoms such as fever and malaise. Wet gangrene is frequently associated with foul odor due to secondary infection [16].

### Body Mass Index (BMI)

Patients’ weight and height were measured, and body mass index (BMI, kg/m²) was calculated and classified as underweight (<18.5), normal weight (18.5–24.9), overweight (25.0–29.9), or obese (≥30.0) [17].

### Bacterial isolation and identification

Wound swab samples were immediately cultured on Blood, MacConkey, and Chocolate agars and incubated overnight at 37 °C. Suspected *P. aeruginosa* colonies were selected and sub-cultured on MacConkey agar to obtain pure isolates. Further characterization was performed using biochemical tests, including peptone water, methyl red, Voges–Proskauer, Simmons citrate, Triple Sugar Iron (TSI), cetrimide agar, oxidase test, and Gram staining [18]. All isolates were additionally confirmed using the automated Vitek®2 system (bioMérieux, France).

### Antibiotic susceptibility test

Antimicrobial susceptibility *test* of *P. aeruginosa* isolates was performed using twelve antibiotic discs (Bioanalyses, Turkey). The disc diffusion method was performed on Mueller-Hinton agar to assess antibiotic susceptibility and resistance. After 24 hours of incubation, the diameters of the inhibition zone around each disc were measured, and compared to standard reference value. Based on the size of the inhibition zones, the isolates were interpreted as susceptible or resistant in accordance with Clinical and Laboratory Standards Institute (CLSI) guidelines (M100 35rd Edition 2025) [19].

### Molecular investigation

#### Genomic DNA extraction.

Genomic DNA of all isolated *P. aeruginosa strains* was extracted, using a commercial extraction kit (PrimePrepTM Genomic DNA Extraction Kit, GeNet Bio, Korea). Following extraction, the concentration and purity of the DNA samples were determined using a NanoDrop spectrophotometer (Thermo scientific, USA). The extracted genomic DNA was subsequently used as a template for polymerase chain reaction (PCR) amplification. All procedures were conducted at the Research Center of the Biology Department, Faculty of Science, University of Zakho.

#### Primers.

A 16S rDNA species-specific primer set was used for the molecular identification of the 16S rDNA gene in *P. aeruginosa.* The primer sequences used were: forward (GGGGGATCTTCGGACCTCA) and reverse (TCCTTAGAGTGCCCACCCG), yielding an expected amplicon size of 956 base pairs as previously described [20]. A positive control for the 16S rDNA gene was obtained from [21] with the accession No. (OR882879).

#### PCR amplification and gel electrophoresis.

The final volume of the PCR amplification reaction was 20 µl consisting of 8 µL of master mix (2x Taq Mix, manufacturer by GDSBio- China, Lot number: 130) 2 µL of forward primer**,** 2 µL of reverse primer**,** 2 µL of genomic DNA (25–50 ng/µL)**,** and 6 µL of PCR-grad. Following preparation of the reaction mixture, the tubes were placed into the thermal cycler for amplification. The amplification conditions are summarized in Table 1. After amplification, the PCR products were analysed by gel electrophoresis. A 1.25% (w/v) agarose gel was prepared using 1 × Tris–Borate–EDTA (TBE) buffer**,** and samples were electrophoresed for 5 minutes at 45 V, followed by 30 minutes at 80 V. DNA bands were subsequently visualized under a UV transilluminator (Cleaver Scientific, UK). The procedure followed the standard protocols described by by Ausubel et al [22].

**Table 1:** The amplification condition of 16S rDNA PCR analysis for 16S rDNA primer.

Initial denaturation	Denaturation	Annealing	Extension	Final extension	Reference
94 °C	92 °C	54 °C	72 °C	72 °C	[20]
2 min	20 sec	20 sec	40 sec.	5 min	
1 cycle	25 cycles			1 cycle	

### Inclusion and exclusion criteria

All clinical samples obtained from diabetic patients aged ≥ 18 years who met the diagnostic criteria for wound infection and attended to hospitals or outpatients’ clinics were included in the study. The exclusion criteria were as follows: (1) patients younger than 18-years of age, (2) patients who declined participation in the study and (3) patient who had received antimicrobial therapy prior to sample collection.

### Ethics

Ethical approval for this study was obtained from Medical and General Health Research Ethics Committee from the Ministry of Health, Duhok Directorate General of Health, under the reference number (29052024-4-2).

### Statistical analysis

raphPad Prism 10.6.1 was used for statistical analysis. Binary logistic regression was employed to calculate odds ratios (ORs), 95% confidence intervals (95% CIs), and p-values. All variables were included in a multivariate logistic regression model. A p-value < 0.05 was considered statistically significant, indicating strong evidence of a relationship between the independent variables and the outcomes under investigation.

## Result

### Demographic characteristics of the diabetic patients with wound infection

A total of 141 patients were included in the analysis. *Pseudomonas aeruginosa*–associated diabetic wound infections (PsA DWIs) were identified in 63 patients (44.68%), whereas non-PsA DWIs were detected in 78 patients (55.32%). The sociodemographic characteristics of patients with diabetic wound infections, with and without *P. aeruginosa*, are presented in Table 2.

**Table 2:** Sociodemographic characteristics of diabetes wound patients with P. aeruginosa and without P. aeruginosa infection.

Characteristic	Total No. (%) 141 (100)	Non-PsA DWIs No. (%) 78 (55.32)	PsA DWIs No. (%) 63 (44.68)	Unadjusted OR [95% CI, p-value]	Adjusted OR [95% CI, p-value]
**Age Group (Year)**					
18-49	29 (20.57)	17 (58.62)	12 (41.38)	Ref	Ref
50-59	45 (31.91)	25 (55.56)	20 (44.44)	1.13 [0.44–2.91, p = 0.80]	1.25 [0.41–3.81, p = 0.69]
60-69	42 (29.79)	23 (54.76)	19 (45.24)	1.17 [0.45–3.05, p = 0.75]	1.07 [0.33–3.48, p = 0.91]
≥ 70	25 (17.73)	13 (52)	12 (48)	1.31 [0.45–3.84, p = 0.63]	1.08 [0.29–4.08, p = 0.91]
Mean ± SD	57.48 ± 10.66	57.26 ± 10.52	57.76 ± 10.91	1.00 [0.97–1.04, p = 0.78]	------------------------
**Sex**					
Female	77 (54.61)	40 (51.95)	37 (48.05)	Ref	Ref
Male	64 (45.39)	38 (59.38)	26 (40.63)	0.74 [0.38–1.45, p = 0.38]	1.02 [0.06–17.7, p = 0.99]
**Region**					
Urban	110 (78.01)	62 (56.36)	48 (43.64)	Ref	Ref
Rural	31 (21.99)	16 (51.61)	15 (48.39)	1.21 [0.55–2.69, p = 0.64]	1.5 [0.60–3.78, p = 0.39]
**BMI (kg/m**^**2**^)					
Normal	24 (17.02)	10 (41.67)	14 (58.33)	Ref	Ref
Overweight	45 (31.91)	32 (71.11)	13 (28.89)	0.29 [0.10–0.82, p = 0.019^*^]	0.35 [0.11–1.13, p = 0.08]
Obesity	72 (51.06)	36 (50)	36 (50)	0.71 [0.28–1.82, p = 0.48]	0.69 [0.23–2.09, p = 0.51]
Mean ± SD	30.28 ± 5.82	29.80 ± 4.99	30.88 ± 6.69	1.03 [0.98–1.096, p = 0.26]	------------------------
**Educational Level**					
Non-educational (illiterate)	84 (59.57)	42 (50)	42 (50)	Ref	Ref
Primary school	30 (21.48)	21 (70)	9 (30)	0.43 [0.18–1.04, p = 0.06]	0.42 [0.14–1.23, p = 0.11]
Secondary and Tertiary school	19 (13.48)	11 (57.89)	8 (42.11)	0.73 [0.27–1.99, p = 0.54]	0.95 [0.26–3.5, p = 0.94]
High educational (Institute or college)	8 (5.67)	4 (50)	4 (50)	1 [0.24–4.27, p = 1]	4.84 [0.48–48.9, p = 0.18]
**Occupational status**					
Daily Labor	14 (9.93)	7 (50)	7 (50)	Ref	Ref
Government Employee	15 (10.64)	12 (80)	3 (20)	0.25 [0.05–1.29, p = 0.098]	0.12 [0.02–0.97, p = 0.046^*^]
Pension	8 (5.67)	7 (87.5)	1 (12.5)	0.14 [0.01–1.49, p = 0.10]	0.05 [0.003–0.96, p = 0.047^*^]
Housewife	73 (51.77)	37 (50.68)	36 (49.32)	0.97 [0.31–3.05, p = 0.96]	0.86 [0.04–16.9, p = 0.92]
Unemployed	31 (21.99)	15 (48.39)	16 (51.61)	1.07 [0.30–3.77, p = 0.92]	1.18 [0.24–5.77, p = 0.84]
**Smoking**					
Non smoker	93 (65.96)	49 (52.69)	44 (47.31)	Ref	Ref
Former smoker	24 (17.02)	16 (66.67)	8 (33.33)	0.56 [0.22–1.43, p = 0.22]	0.81 [0.23–2.85, p = 0.74]
Current smoker	24 (17.02)	13 (54.17)	11 (45.83)	0.94 [0.38–2.32, p = 0.90]	1.94 [0.49–7.63, p = 0.35]
**Family History of DM**					
No	67 (47.52)	40 (59.7)	27 (40.3)	Ref	Ref
Yes	74 (52.48)	38 (51.35)	36 (48.65)	1.40 [0.72–2.74, p = 0.32]	1.45 [0.66–3.22, p = 0.36]

Note: **PsA** = *Pseudomonas aeruginosa,* **DWIs** = Diabetes Wound Infections, **OR**= odd ratio, * = statistically significant difference.

The age distribution among patients with diabetes mellitus (DM) showed no statistically significant association with PsA infection. The highest proportion of cases was observed in the 50–59-year age group (31.91%), with a higher prevalence of non-PsA DWIs than PsA DWIs. The odds ratios (ORs) for PsA infection were 1.13 (95% CI: 0.44–2.91, p = 0.80), 1.17 (95% CI: 0.45–3.05, p = 0.75), and 1.31 (95% CI: 0.45–3.84, p = 0.63) for the 50–59, 60–69, and ≥70-year age groups, respectively, compared with the reference group (18–49 years). The mean age was also not significantly associated with PsA infection (OR = 1.00, 95% CI: 0.97–1.04, p = 0.78).

Similarly, sex distribution did not differ significantly between the two groups. Males had an OR of 0.74 (95% CI: 0.38–1.45, p = 0.38) compared with females. Females comprised 54.61% of the total sample, with comparable proportions in the non-PsA (51.95%) and PsA DWI (48.05%) groups.

Most participants resided in urban areas (78.01%), compared with 21.99% in rural areas. In both settings, the prevalence of PsA DWIs was lower than that of non-PsA DWIs. However, the difference was not statistically significant, with an OR of 1.21 for rural residence (95% CI: 0.55–2.69, p = 0.64).

Among all patients with diabetes mellitus (DM), obesity was the most prevalent BMI category (51.06%), with an equal distribution of PsA and non-PsA diabetic wound infections (DWIs). This was followed by the overweight group (31.91%) and the normal-weight group (17.02%). The highest proportion of PsA DWIs was observed in the normal-weight group (58.33%). Overweight individuals had significantly lower odds of PsA infection compared with the normal-weight group (OR = 0.29, 95% CI: 0.10–0.82, p = 0.019), reflecting a lower proportion of PsA DWIs (28.89%) relative to non-PsA DWIs (71.11%). In contrast, obesity was not significantly associated with PsA infection (OR = 0.71, 95% CI: 0.28–1.82, p = 0.48). Furthermore, the overall mean BMI was not significantly associated with PsA infection (OR = 1.03, 95% CI: 0.98–1.096, p = 0.26

Educational attainment varied among participants: 59.57% were illiterate, 21.48% had primary education, 13.48% had secondary or tertiary education, and 5.67% had higher education. Across all categories, the prevalence of PsA diabetic wound infections (DWIs) was less than or equal to that of non-PsA DWIs. Compared with the illiterate group (reference), the odds ratios (ORs) for PsA infection were 0.43 (95% CI: 0.18–1.04, p = 0.06) for primary education, 0.73 (95% CI: 0.27–1.99, p = 0.54) for secondary/tertiary education, and 1.00 (95% CI: 0.24–4.27, p = 1.00) for higher education; none of these associations were statistically significant.

Approximately three-quarters (73.76%) of patients were not formally employed, including housewives (51.77%) and unemployed males (21.99%). The remaining participants were government employees (10.64%), daily laborers (9.93%), and pensioners (5.67%). A higher proportion of PsA DWIs was observed only among unemployed individuals (51.61%). However, no statistically significant association was found between occupational status and PsA infection in the unadjusted analysis, with daily laborers serving as the reference group. The ORs were 0.25 (p = 0.098) for government employees, 0.14 (p = 0.10) for pensioners, 0.97 (p = 0.96) for housewives, and 1.07 (p = 0.92) for unemployed individuals.

Regarding smoking status, non-smokers constituted the largest proportion of patients (65.96%), followed by former smokers (17.02%) and current smokers (17.02%). Non-smokers had a slightly higher prevalence of non-PsA DWIs compared with PsA DWIs (52.69% vs. 47.31%). In contrast, PsA DWIs were less frequent among former and current smokers compared with non-PsA DWIs. Neither former smoking (OR = 0.56, p = 0.22) nor current smoking (OR = 0.94, p = 0.90) showed a statistically significant association with PsA infection when compared with non-smokers. The number of patients with and without a family history of diabetes mellitus was nearly equal (74 vs. 67). In both groups, the prevalence of PsA DWIs was slightly lower than that of non-PsA DWIs; however, this difference was not statistically significant (OR = 1.40, p = 0.32).

In the multivariable analysis, some associations changed. The previously observed significant association for overweight individuals was no longer statistically significant (p = 0.08). In contrast, occupational categories such as government employees (p = 0.046) and pensioners (p = 0.047) became statistically significant, indicating an independent association with reduced odds of PsA infection.

### The clinical characteristics of diabetic patients with PsA DWIs and non-PsA DWIs

The clinical characteristics of PsA diabetic wound infections (DWIs) and non-PsA DWIs are presented in Table 3. Among the study population, 14 patients had glycated hemoglobin (HbA1c) levels between 4 and 6.9, while 127 patients had HbA1c levels ranging from 7 to 16. The overall mean HbA1c level was 9.23 ± 2.18.The mean HbA1c level was slightly higher in patients with non-PsA DWIs compared with those with PsA DWIs (9.25 ± 1.98 vs. 9.20 ± 2.43); however, this difference was not statistically significant (OR = 0.99, 95% CI: 0.85–1.15, p = 0.90).

**Table 3:** Clinical characteristics of diabetes wound patients with *P. aeruginosa* and without *P. aeruginosa* infection.

Characteristic	Total No. (%) 141 (100)	Non-PsA DWIs No. (%) 78 (55.32)	PsA DWIs No. (%) 63 (44.68)	Unadjusted OR [95% CI, p-value]	Adjusted OR [95% CI, p-value]
**HBA1C (Mean ±SD)**	9.23 ± 2.18	9.25 ± 1.98	9.20 ± 2.43	0.99 [0.85–1.15, p = 0.90]	0.88 [0.73–1.07, p = 0.19]
**Duration of DM by year (Mean ±SD)**	13.92 ± 8.42	13.97 ± 8.99	13.86 ± 7.72	0.99 [0.96–1.04, p = 0.87]	0.98 [0.92–1.03, p = 0.40]
**History of ulcer by months (Wound infection)**	9.86 ± 29.29	13.13 ± 38.41	5.82 ± 11.34	0.99 [0.97–1.01, p = 0.16]	0.98 [0.95–1.01, p = 0.23]
**Location of wound infection (skin ulcer)**					
Non-Leg region	30 (21.28)	22 (73.33)	8 (26.67)	Ref	Ref
Leg region (Leg and Foot)	111 (78.72)	56 (50.45)	55 (49.55)	2.7 [1.11–6.58, p = 0.03^*^]	3.94 [1.24–12.5, p = 0.02^*^]
**Type of Wound infection**					
Dry	50 (35.46)	32 (64)	18 (36)	Ref	Ref
Wet	86 (60.99)	43 (50)	43 (50)	1.78 [0.87–3.64, p = 0.12]	2.13 [0.90–5.22, p = 0.09]
Both	5 (3.55)	3 (60)	2 (40)	1.19 [0.18–7.77, p = 0.86]	0.63 [0.07–5.68, p = 0.68]
**Degree of Wound infection (Lesion Severity) (Clinical presentations)**					
Abscess	11 (7.8)	7 (63.64)	4 (36.36)	Ref	Ref
Cellular death	3 (2.13)	2 (66.67)	1 (33.33)	0.88 [0.06–12.97, p = 0.92]	0.59 [0.03–13.3, p = 0.75]
Gangrene	6 (4.26)	3 (50)	3 (50)	1.75 [0.23–13.2, p = 0.59]	1.53 [0.14–17.1, p = 0.71]
Ulcer	37 (26.24)	23 (62.16)	14 (37.84)	1.07 [0.26–4.31, p = 0.93]	1.93 [0.32–11.73, p = 0.48]
Ulcer with Cellular Death	73 (51.77)	38 (52.05)	35 (47.95)	1.61 [0.43–5.98, p = 0.48]	2.02 [0.36–11.52, p = 0.43]
Ulcer with Cellular death and Gangrene	11 (7.8)	5 (45.45)	6 (54.55)	2.10 [0.38–11.6, p = 0.40]	2.52 [0.26–24.01, p = 0.42]
**Comorbid condition**					
Hypertension	79 (56)	41 (51.90)	38 (48.10)	1.35 [0.57–3.21, p = 0.5]	1.69 [0.62–4.59, p = 0.30]
Cardiac disease	43 (30.5)	26 (60.47)	17 (39.53)	0.74 [0.33–1.67, p = 0.46]	0.58 [0.22–1.5, p = 0.26]
Renal disease	33 (23.4)	20 (60.61)	13 (39.39)	0.74 [0.29–1.91, p = 0.54]	0.55 [0.18–1.68, p = 0.30]
Eye problem	70 (49.6)	40 (57.14)	30 (42.86)	0.73 [0.30–1.79, p = 0.5]	0.72 [0.26–1.99, p = 0.53]
Periodontal (gum) disease	31 (22)	15 (48.39)	16 (51.61)	1.61 [0.66–3.95, p = 0.3]	1.84 [0.65–5.2, p = 0.25]
Other infection	32 (22.7)	21 (65.63)	11 (34.38)	0.5 [0.20–1.2, p = 0.12]	0.67 [0.24–1.91, p = 0.46]
No other comorbid conditions	29 (20.57)	17 (58.62)	12 (41.38)	0.7 [0.19–2.3, p = 0.52]	0.69 [0.17–2.81, p = 0.61]
**Types of Diabetic treatment**					
Diets Only	5 (3.55)	4 (80)	1 (20)	Ref	Ref
Insulin Only	4 (2.84)	2 (50)	2 (50)	4.0 [0.21–75.7, p = 0.36]	1.76 [0.06–53.75, p = 0.75]
Oral Hypoglycemic Tablets Only	19 (13.48)	10 (52.63)	9 (47.37)	3.60 [0.34–38.5, p = 0.29]	1.19 [0.09–16.35, p = 0.9]
Diets and Insulin	33 (23.4)	20 (60.61)	13 (39.39)	2.60 [0.26–25.9, p = 0.42]	1.15 [0.09–14.92, p = 0.92]
Diets and Oral Hypoglycemic Tablets	44 (31.21)	22 (50)	22 (50)	4.0 [0.41–38.7, p = 0.23]	1.73 [0.14–20.9, p = 0.67]
Insulin and Oral Hypoglycemic Tablets	6 (4.26)	2 (33.33)	4 (66.67)	8.0 [0.50–127.9, p = 0.14]	2.69 [0.13–56.28, p = 0.52]
Diet, Insulin and Hypoglycemic Tablets	25 (17.73)	14 (56)	11 (44)	3.14 [0.31–32.3, p = 0.34]	1.29 [0.09–17.54, p = 0.85]
No treatment	5 (3.55)	4 (80)	1 (20)	1.0 [0.05–22.2, p = 1.0]	0.13 [0.003–5.63, p = 0.30]
**Exercise Status**					
No	127 (90.07)	69 (54.33)	58 (45.67)	Ref	Ref
Yes	14 (9.93)	9 (64.29)	5 (35.71)	0.66 [0.21–2.08, p = 0.48]	0.50 [0.12–2.08, p = 0.34]
**Wound Dressing Applied**					
No	12 (8.51)	7 (58.33)	5 (41.67)	Ref	Ref
Yes	129 (91.49)	71 (55.04)	58 (44.96)	1.14 [0.35–3.79, p = 0.83]	0.46 [0.09–2.38, p = 0.35]

Note: **PsA** = *Pseudomonas aeruginosa,* **DWIs** = Diabetes Wound Infections, **OR**= odd ratio * = statistically significant difference.

The mean duration of diabetes was 13.86 ± 7.72 years in patients with PsA diabetic wound infections (DWIs) and 13.97 ± 8.99 years in those with non-PsA DWIs, with no statistically significant difference between the groups (OR = 0.99, p = 0.87). Similarly, the duration of wound infection (in months) did not differ significantly between the two groups (OR = 0.99, p = 0.16), although a higher mean duration was observed in non-PsA DWI cases compared with PsA DWI cases (13.13 ± 38.41 vs. 5.82 ± 11.34 months). In terms of wound location, infections in the leg region were the most common (78.72%), comprising 49.55% of PsA DWIs and 50.45% of non-PsA DWIs. Leg infections were significantly associated with the outcome, with approximately 2.7-fold higher odds of PsA infection (OR = 2.70, 95% CI: 1.11–6.58, p = 0.03).

Regarding wound type, wet wounds were the most prevalent (60.99%) and showed higher, though not statistically significant, odds of PsA infection (OR = 1.78, 95% CI: 0.87–3.64, p = 0.12). This was followed by dry wounds (35.46%) and mixed wounds (3.55%), with ORs of 1.19 (95% CI: 0.18–7.77, p = 0.86). Among wet wounds, PsA and non-PsA DWIs were equally distributed (50% each). In contrast, PsA DWIs accounted for 39% of dry wounds and 40% of mixed wounds, whereas non-PsA DWIs represented 64% and 60% of these categories, respectively.

A higher prevalence of PsA DWIs was observed in ulcers with cellular necrosis and gangrene (54.55%) compared with non-PsA DWIs (45.45%). In other lesion types, PsA DWIs were equal to or less frequent than non-PsA DWIs (36.36% in abscesses, 33.33% in cellular necrosis, 50% in gangrene, 37.84% in ulcers, and 47.95% in ulcers with cellular necrosis). Although some categories showed higher odds ratios (ORs) than abscesses, none were statistically significant (all p > 0.05) and confidence intervals (CIs) were wide. Among 112 patients with comorbidities, hypertension was most common (56%). PsA DWIs were more frequent in patients with periodontal disease (51.61%) and hypertension (48.1%), but no significant associations were found (all p > 0.05; CIs included 1). Regarding treatment, 31.12% used diet plus oral hypoglycemic agents, while 23.4% used diet plus insulin. PsA DWIs were most common among patients receiving insulin plus oral agents (66.67%). However, treatment type was not significantly associated with PsA DWIs (all p > 0.05; CIs included 1). Most patients (90.07%) did not exercise. Non-PsA DWIs were more prevalent in both active and inactive groups, with no significant association (p = 0.48), although exercise showed a lower OR (0.66; 95% CI: 0.21–2.08). Wound dressing was used by 91.49% of patients. PsA DWIs were less frequent than non-PsA DWIs, with no statistically significant difference.

All independent variables were not statistically significant predictors of PsA in the multivariable logistic regression analysis, except for wound location (leg region), which was significant (p = 0.02).

### Antibiotics susceptibility profile

Table 4 summarizes the antimicrobial susceptibility profile of *P. aeruginosa* isolates. High resistance rates were observed for aztreonam (89%) and ceftazidime (84%), followed by cefepime (81%) and tobramycin (86%). Moderate resistance was noted for amikacin (75%), ciprofloxacin (79%), and levofloxacin (79%), while lower resistance rates were observed for gentamicin (62%) and norfloxacin (56%).

**Table 4:** Antibiotics susceptibility profile among *P. aeruginosa* bacteria.

Category	Antibiotics/µg	Resistance No. (%)	Sensitive No. (%)
Monobactam	Aztreonam (ATM 30)	56 (89)	7 (11)
Carbapenem	Imipenem (IPM 10)	33 (52)	30 (48)
	Meropenem (MEM 10)	26 (41)	37 (59)
3^rd^ generation Cephalosporins	Ceftazidime (CAZ 30)	53 (84)	10 (16)
4^th^ generation Cephalosporins	Cefepime (FEP 10)	51 (81)	12 (19)
Aminoglycosides	Amikacin (AK 10)	47 (75)	16 (25)
	Gentamicin (CN 10)	39 (62)	24 (38)
	Tobramycin (TOB 10)	54 (86)	9 (14)
Fluoroquinolones	Ciprofloxacin (CIP 10)	50 (79)	13 (21)
	Norfloxacin (NOR 30)	35 (56)	28 (44)
	levofloxacin (LEV 5)	50 (79)	13 (21)
Polypeptides	Colistin (CT 10)	23 (37)	40 (63)

Among carbapenems, resistance to imipenem and meropenem was 52% and 41%, respectively. Notably, colistin demonstrated the lowest resistance rate (37%) and the highest sensitivity (63%) among all tested antibiotics. Overall, *P. aeruginosa* isolates exhibited high levels of multidrug resistance, with relatively better susceptibility to colistin and carbapenems compared to other antibiotic classes.

### Molecular identification

**Fig 1:**
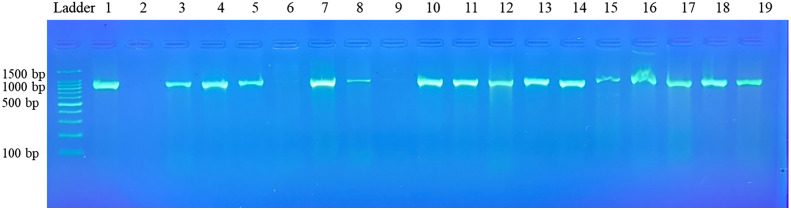
The amplification of PCR for species identification of P. aeruginosa using 16S rDNA primer. The left lane shows the DNA ladder (100–1500 bp), lane 1: representative positive control, lane 2: representative negative control, lanes 3-19 representative patient’s samples. All tested isolates exhibit a distinct single band at approximately 956 bp, confirming the successful amplification of the target gene and molecular identification of P. aeruginosa.

All *Pseudomonas aeruginosa* isolates were confirmed by PCR amplification of the 16S rDNA gene, producing a single band of 956 base pairs (Fig 1).

## Discussion

Patients with diabetes mellitus represent a major risk group for skin wound infections, which may be caused by a variety of microorganisms, including *P. aeruginosa*. Previous studies on *P. aeruginosa* in diabetic patients have primarily focused on diabetic foot infections (DFIs). In contrast, the present study investigated P*. aeruginosa*–associated wound infections across all anatomical sites, reporting a prevalence of 63 cases (44.68%) among 141 patients. The identification of *P.s aeruginosa* in this study was confirmed using conventional biochemical tests, the Vitek®2 system, and 16S rDNA PCR amplification. The use of PCR provided a high level of specificity and accuracy in bacterial identification, thereby strengthening the reliability of the laboratory findings. This molecular confirmation reduces the possibility of misidentification that may occur with conventional methods alone and ensures more precise detection of *P. aeruginosa* in diabetic wound infections. Therefore, the combination of phenotypic and molecular techniques improves the validity of the reported prevalence and supports the robustness of the study results. This finding is consistent with a study conducted in Duhok city, in which all bacterial isolates were similarly confirmed using the 16S rDNA gene [23].

The prevalence of *P. aeruginosa* among DFU observed in this study is comparable to reports from Najaf, Iraq (34.4%) [9], Iran (35%) [24], and various regions of India, including North (23.7%), South (29.8%), North-East (11.89%), East (11.7%) and West India (27%) [25]. Similarly, a study conducted in Erbil, Iraq, reported that 53 of 150 DFU samples (35.33%) were positive with *P. aeruginosa* [11]. In the present study, PsA DWIs were less frequent than non-PsA DWIs, which contrasts with findings from a study in Anhui Province, China, where *P. aeruginosa* accounted for only 9% of infections compared to 91% for non-*P. aeruginosa* pathogens [26]. These variations may be attributed to differences in causative microorganisms, geographic location, and the severity and types of infections [27].

The current study found a higher prevalence rate of DM among females compared to males. Additionally, a higher rate of PsA DWIs was observed in the 50–59 age groups. A comparable study conducted in Kermanshah Province, Iran, reported a female prevalence of 57.4% and a male prevalence of 42.6% [1]. In contrast, a previous study in Najaf City, Iraq, found a lower proportion of females (44.3%) compared to males (55.7%) among 282 DM patients with foot wound infections [9]. Furthermore, another study in Iraq reported a male to female ratio of 2.5:1, while demonstrating a similar age distribution, with the highest incidence of DFI occurring in the 51–60 age groups [28]. The higher proportion of cases among females in the present study may be attributed to cultural variations influencing access to healthcare services [1].

The present study indicated that individuals residing in urban areas are more likely to develop wound infection compared to those living in rural areas. This study is consistent with a study conducted in Ethiopia, by Molla et al (2023), which reported a higher prevalence of DFI in urban population (62.4%) compared to rural (37.6%) [29]. Similarly, a study carried out in Erbil City, Iraq, demonstrated that infection rates were higher in urban areas (54%) than in rural areas (46%) [11]. These variations may be attributed to differences in study populations and methodologies. Furthermore, several indicators used to assess socioeconomic status at the neighborhood level may provide insight into the environmental and social factors influencing the risk of infection among residents [30].

Conversely, obese individual exhibited the highest rates of infection compared to those who were overweight or normal weight. However, the prevalence rate of PsA DWIs was highest among individuals with normal weight, followed by overweight and obese groups. This finding is consistent with a study conducted in Iraq, which reported a higher incidence of diabetic foot infections (DFIs) among obese participants [5]. Additionally, a study from the United States found that the mean body mass index (BMI) among DFI patients with and without *P. aeruginosa* infection was 33.6% and 30.6%, respectively [31]. Over recent decades, obesity rates have increased dramatically, leading the World Health Organization to classify as a global epidemic and a major threat to public health [17]. Obesity is associated with several comorbid conditions, such as atherosclerosis, which may contribute to the development of diabetic foot ulcers (DFUs) and subsequent infections [5].

Educational level varied among participants, with illiterate individuals showing a higher likelihood of infection compared to other groups. In all categories, the prevalence of *P. aeruginosa* diabetic wound infections (DWIs) was lower than or equal to that of non-*P. aeruginosa* DWIs. To date, no studies have specifically examined the relationship between educational level and *P. aeruginosa* DWIs. Consistent with the present findings, a study conducted in Colombia reported that the majority of individuals with DM had not completed primary education [32]. Similarly, a study from Kirkuk City, Iraq, reported an illiteracy rate of 24%, compared to only 4% among those with higher education [4]. In contrast, several other studies have reported conflicting results, indicating higher rates of diabetic foot infections (DFIs) among individuals with higher educational attainment, including those with high school and university education [5,33] while lower rates were observed among illiterate patients [29].

Moreover, evidence suggests that individuals with lower levels of education are more likely to develop DM and experience its complications [30]. These findings indicated that the level of education is important in taking care of metabolic diseases and the promotion of health-protective behaviors [4]. Patients with limited education may have reduced awareness regarding glycemic control and proper wound care practices, increasing their susceptibility to infection.

This study is partly consistent with findings from Iraq, where employment status was distributed as follows: housewives (37.6%), employees (12.4%), daily laborers (22.8%), and pensioners (27%) [5]. Similarly, a study from Ethiopia reported housewives (19%), government employees (26.8%), daily laborers (5.3%), pensioners (5%), unemployed (3.8%), and others (farmers and merchants) [29]. In our study, the highest rates of PsA DWIs were observed among non-working, non-occupational, and uneducated patients of both sexes.

Smoking patterns in our study were similar to a previous report, in which most patients were non-smokers (74.9%), followed by smokers (1.6%) and ex-smokers (9.5%) [5]. Another study reported a higher rate of PsA in foot infections compared with non-PsA cases (37% vs. 19%) [8]. In our study, no significant association was found between smoking status and PsA DWIs, although non-smokers had higher rates than current and former smokers.

The distribution of family history of diabetes in our study differs from findings in Iraq, where rates among DFI patients were negative (47%), positive (36%), and unknown (16.2%) [5]. Similarly, other studies reported negative rates of 62.2% and 72.7%, and positive rates of 37.8% and 27.3%, respectively [29,34]. Evidence consistently shows that individuals with a family history of diabetes are at higher risk of developing the disease, likely due to genetic factors [34,35].

The mean HbA1c in our study was comparable to previous reports (8.3 ± 2.2 [24], 8.94 ± 2.1 [36], and 8.61 ± 2.24 [37]), although one study reported a higher mean (10.26 ± 2.0) [5]. Similarly, a U.S. study reported a mean HbA1c of 8.3% [8]. HbA1c was higher in patients with PsA DFI than in non-PsA DFI (8.5% vs. 7.3%). Chronic hyperglycemia impairs wound healing, increases wound size, and is associated with complications and mortality [38,39]. Therefore, maintaining optimal HbA1c levels is essential for preventing diabetic foot complications.

Several studies have reported the mean ± SD of diabetes duration, including 14.5 ± 4.5 years [24], 13.6 ± 8.5 years [36], and 13.54 ± 6.98 years [5]. One study reported that 40.3% of patients had a duration ≤10 years, while 59.7% had > 10 years [33], and another reported 11.1% [37]. The mean ulcer duration among DFU patients has been reported as 14.5 ± 4.5 months [24] and 1.8 months [37]. In contrast to studies focusing solely on foot infections, our study included multiple anatomical sites. Toe infections were most common (44%), including 30% PsA and 45% non-PsA DFI. The lateral plantar region accounted for 29% (22% PsA, 30% non-PsA), the heel for 16% (19% PsA, 16% non-PsA), and other sites for 14% (37% PsA, 11% non-PsA). Unspecified sites comprised 9% (4% PsA, 9% non-PsA) [8]. A significant association was observed between wound location and DM (p = 0.001), except for foot-related sites.

Based on infection severity, DFIs in our study were mainly ulcers (70%), followed by abscesses (21%) and combined ulcer–abscess lesions (10%). PsA DFIs were more frequent in ulcers (82%) than in non-PsA cases (68%), whereas abscesses and mixed lesions were more common in non-PsA DFIs [8]. Other studies reported varying distributions, including ulcers (44%), cellulitis (20%), abscesses (32%), gangrene (24%), and neuropathic ulcers (4%) [7], while another found ulcers in 50.2% of cases [37]. Overall, wound infection is a common complication of diabetes, often resulting from ulceration and inflammation, and may progress to gangrene and lower limb amputation, contributing to significant morbidity, hospitalization, and mortality [7].

Several studies have examined DFU and associated complications. In one study of 54 DFU cases, hypertension, retinopathy, nephropathy, neuropathy, and osteomyelitis were reported in 11, 8, 11, 24, and 10 patients, respectively [24]. Another study (2023) found that 62.2% of patients had comorbidities, most commonly hypertension (30.8%), followed by combinations of cardiovascular, renal, and other chronic diseases, while 37.8% had none [29]. Similarly, another report showed hypertension (43.2%) as the most frequent comorbidity, followed by hypercholesterolemia (10.7%). Complications were present in 16% of cases, including nephropathy (1.9%), retinopathy (5.8%), neuropathy (4.9%), and other conditions, while 84% had no complications [33]. Ultimately, poor blood supply, infection, and neuropathy may progress to tissue necrosis, osteomyelitis, gangrene, and amputation [7,39]. The presence of multiple (≥3) complications in DM patients with wound infection is an indicator of increased amputation risk. Therefore, improved education, early detection, and timely management are essential to reduce major amputations [26].

Regarding treatment methods, one study reported that among DM patients, 26.6% used oral hypoglycemic agents, 43.6% insulin, 29.4% both insulin and oral agents, 0.3% other treatments, and 3.3% received no treatment [37]. Another study reported diet alone (10.7%), oral hypoglycemic agents (55.8%), insulin alone (8.3%), combined therapy (12.6%), and 12.6% with no treatment [29]. Similarly, oral hypoglycemic agents, insulin, and combination therapy were reported in 42.9%, 31.6%, and 25.6% of patients, respectively [29]. In addition, 43.2% of DM patients engaged in exercise, while 56.8% did not [33]. The risk of developing type 2 diabetes can be reduced through a healthy diet and regular exercise. Improved insulin sensitivity reduces hepatic glucose production, leading to lower HbA1c levels, which in turn decreases the risk of diabetes-related complications [40].

Most patients are managed with wound dressing and debridement [7], and about two-thirds of DM patients with wound infection receive antimicrobial dressings [37]. Selection of wound dressings should follow best practice recommendations (BPRs) and clinical practice guidelines (CPGs) to ensure appropriate moisture balance, optimal wound care, and support for surgical cleansing. Antimicrobial dressings may also promote faster healing compared with non-antimicrobial dressings [6].

The multivariate logistic regression results of the present study differ from previous reports, which identified various independent predictors of infection in DFU/DFI patients. One study reported that age > 65 years (OR=1.242, 95% CI: 1.251–2.114, p = 0.001) and HbA1c > 7% (OR=2.155, 95% CI: 1.287–2.946, p = 0.032) were the only significant factors associated with infection in DFUs [24]. In another study, smoking and insulin use were significant in unadjusted analysis (OR=2.6, 95% CI: 1.1–6, p = 0.022 and OR=0.21, 95% CI: 0.08–5.2, p < 0.001, respectively), but they lost significance or were excluded in the final regression model [8]. Furthermore, a study on DFU-related amputation found that infection site was associated with lower limb amputation in both univariate (OR=0.347, 95% CI: 0.790–0.926, p < 0.001) and multivariate analyses (OR=0.816, 95% CI: 0.724–0.919, p < 0.001) [26].

This study has several limitations. Its cross-sectional design limits the ability to establish causal or temporal relationships between risk factors and *P. aeruginosa* infection. In addition, being conducted in a single province with a relatively small sample size may restrict the generalizability of the findings. However, the study also has important strengths. Clinical wound samples were collected over a 12-month period, minimizing seasonal variation. Samples were obtained from multiple hospitals and private clinics in Zakho and Duhok, improving representativeness. Identification of P. aeruginosa was confirmed using biochemical methods and 16S rDNA PCR, ensuring high diagnostic accuracy. The study also included a broad range of demographic, clinical, and behavioral variables (e.g., age, BMI, smoking, wound characteristics, and glycemic control), allowing a comprehensive assessment of potential risk factors. Finally, antimicrobial susceptibility testing was performed using CLSI-standard disc diffusion methods, ensuring reliable and comparable resistance data.

## Conclusion

Diabetic patients with wound infections represent complex cases that may lead to major complications. In the present study, most sociodemographic factors, including age, sex, residence, education level, smoking status, and family history of diabetes, were not significantly associated with *P. aeruginosa* diabetic wound infections in both unadjusted and adjusted analyses. BMI showed a significant association in the unadjusted model for overweight patients (p = 0.019), but this was not maintained after adjustment. In the multivariate analysis, only occupational status showed a significant association, where government employees (p = 0.046) and pensioners (p = 0.047) had a lower likelihood of *P. aeruginosa* infection compared with daily laborers. Overall, sociodemographic characteristics had limited independent predictive value for *P. aeruginosa* DWIs, except for occupational status. In this study, most clinical variables—including HbA1c level, duration of diabetes, ulcer history, comorbidities, treatment type, exercise status, and wound dressing—were not significantly associated with *P. aeruginosa* diabetic wound infections in both unadjusted and adjusted logistic regression analyses. The only significant independent predictor was wound location, where infections involving the leg region showed a significantly higher risk of *P. aeruginosa* infection (adjusted OR = 3.94, p = 0.02). Overall, these findings suggest that anatomical site of infection plays a key role in *P. aeruginosa* occurrence, while other clinical and behavioral factors showed no independent association. *P. aeruginosa* isolates showed high levels of multidrug resistance, with the highest resistance observed against aztreonam, ceftazidime, and tobramycin. Carbapenems showed moderate resistance, while colistin remained the most effective antibiotic with the highest sensitivity rate.

Therefore, early detection and prompt management of wounds are essential to prevent progression to deep tissue infection. Individuals with a family history of diabetes should be particularly aware of the risk of earlier onset, especially in the presence of high BMI. Improving patient outcomes also depends on strengthening the education level and training of healthcare professionals, particularly nurses, who play a key role in patient education on wound care and complication prevention. Strengthening infection control practices, promoting rational antibiotic use, and improving patient education on wound care are essential to reduce complications and antimicrobial resistance in diabetic patients. In addition, further clinical trials are needed to guide the selection of appropriate antimicrobial agents for wound dressings to ensure optimal patient care

## Supporting information

S1 Raw Image The left lane shows the DNA ladder (100–1500 bp), lane 1: representative positive control, lane 2: representative negative control, lanes 3-19 representative patient’s samples. All tested isolates exhibit a distinct single band at approximately 956 bp, confirming the successful amplification of the target gene and molecular identification of *P. aeruginosa.* (PDF)
