# Supplementary material for: Prevalence and associated risk factors of Pseudomonas aeruginosa infection in Diabetic wounds in Duhok province, Iraq
Source: PLoS One. 2026 Jul 24;21(7):e0354256. doi: 10.1371/journal.pone.0354256 (PMC13399349; doi:10.1371/journal.pone.0354256)

Original unadjusted and uncropped gel image (Supported Fig 1)

The gel images were captured using a Gel Doc™ XR+ imaging system (Bio-Rad Laboratories, USA) equipped with a UV transilluminator (Cleaver Scientific Ltd., UK).

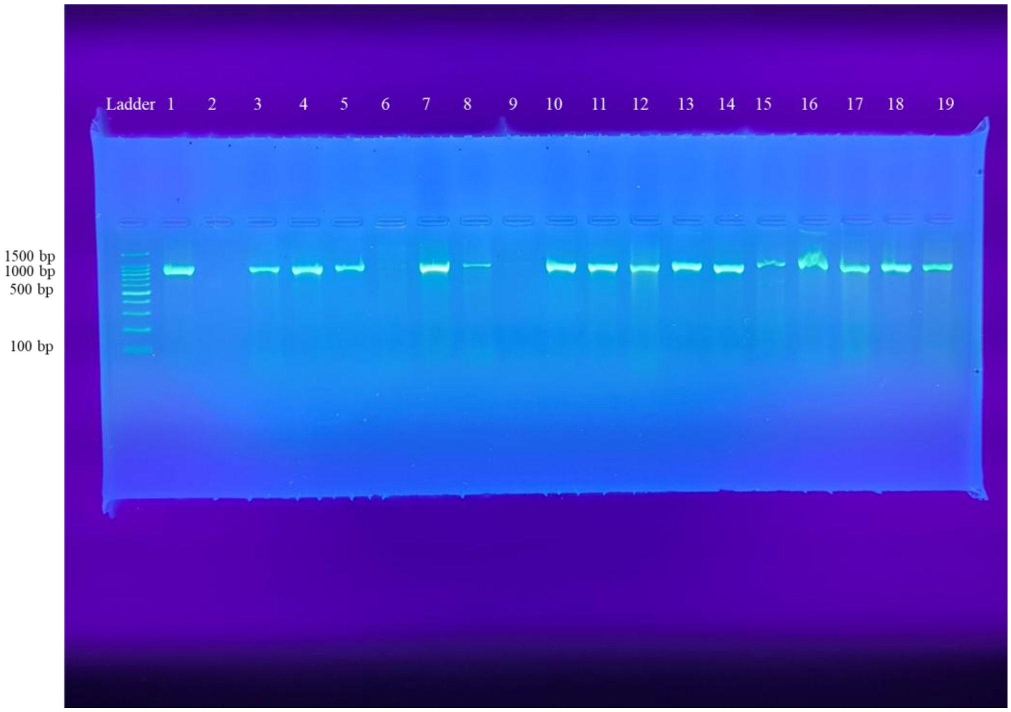

Fig 1: The amplification of PCR for species identification of P. aeruginosa using 16S rDNA primer. *The left lane shows the DNA ladder (100–1500 bp), lane 1: representative positive control, lane 2: representative negative control, lanes 3-19 representative patient's samples. All tested isolates exhibit a distinct single band at approximately 956 bp, confirming the successful amplification of the target gene and molecular identification of P. aeruginosa*.

Gel used in the manuscript:

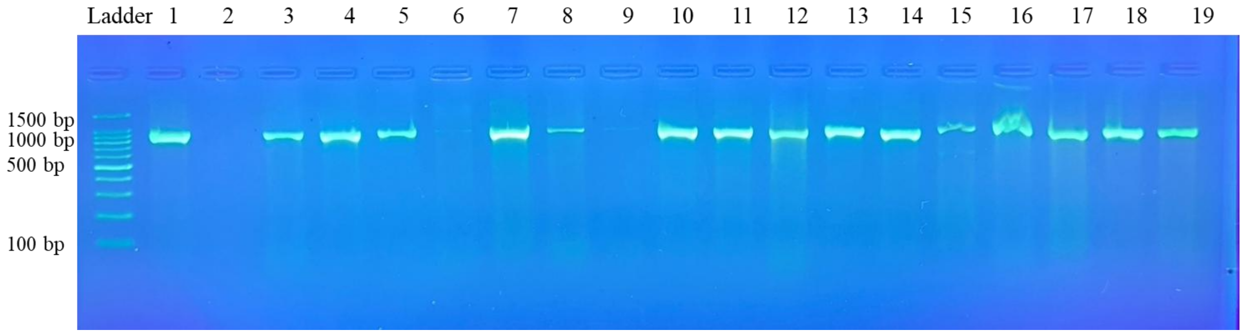

Supplement: S1 Raw Image — (PDF) [file pone.0354256.s001.pdf]
