# Supplementary material for: Prevalence and associated risk factors of Pseudomonas aeruginosa infection in Diabetic wounds in Duhok province, Iraq
Source: PLoS One. 2026 Jul 24;21(7):e0354256. doi: 10.1371/journal.pone.0354256 (PMC13399349; doi:10.1371/journal.pone.0354256)
Supplement: Attachment — Submitted filename: PONE-D-25-68291_R1.pdf [file pone.0354256.s003.pdf]

PLOS One

Prevalence and associated risk factors of Pseudomonas aeruginosa infection in Diabetic wounds in Duhok province, Iraq

--Manuscript Draft--

Manuscript Number:	PONE-D-25-68291R1
Article Type:	Research Article
Full Title:	Prevalence and associated risk factors of Pseudomonas aeruginosa infection in Diabetic wounds in Duhok province, Iraq
Short Title:	Prevalence and risk factors of Pseudomonas aeruginosa in Diabetic wounds
Corresponding Author:	Ibrahim A. Naqid, Ph.D<br>University of Zakho<br>Zakho/Duhok, Kurdistan Region/Iraq IRAQ
Keywords:	Pseudomonas aeruginosa; Diabetic wound infection; prevalence; risk factors; antibiotic susceptibility; Duhok Province; Iraq
Abstract:	Background and aims<br>Diabetes mellitus is a chronic metabolic disorder and a major global health problem, predisposing patients to wound infections, with Pseudomonas aeruginosa being a frequent and resistant pathogen. This study aimed to identify P. aeruginosa, assess associated risk factors, and determine its antibiotic susceptibility pattern in diabetic wound infections in Duhok Province, Kurdistan Region, Iraq.<br>Methods<br>This cross-sectional study was conducted from June 2024 to July 2025 in Duhok Province, Kurdistan Region, Iraq. A total of 141 wound swab samples were collected from diabetic patients aged ≥18 years. P. aeruginosa was identified using biochemical tests, Vitek®2, and 16S rDNA PCR. Antimicrobial susceptibility testing was performed by the disc diffusion method on Mueller–Hinton agar, and data were analyzed using GraphPad Prism software.<br>Results<br>Out of 141 wound infection samples collected from diabetic patients, P. aeruginosa was isolated in 44.68% of cases. Overall, sociodemographic factors showed no significant association with PsA infection, except for occupational status in the multivariable analysis 0.12 [0.02-0.97, p=0.046] and overweight 0.29 [0.10-0.82, p=0.019]. Clinically, most variables including HbA1c, duration of diabetes, comorbidities, treatment type, and wound characteristics were not significantly associated with PsA infection. The only significant predictor was wound location, with leg-region infections showing a higher risk of PsA 3.94 [1.24-12.5, p=0.02]. P. aeruginosa isolates showed high levels of multidrug resistance, especially to aztreonam, ceftazidime, cefepime, and tobramycin. Colistin showed the lowest resistance and highest sensitivity, followed by relatively better susceptibility to carbapenems.<br><br>Conclusion<br>P. aeruginosa is a common cause of wound infections in patients with diabetes mellitus, with its prevalence significantly associated with body mass index, occupational status and wound location, with leg-region wounds showing a higher risk. P. aeruginosa isolates demonstrated high multidrug resistance, although colistin and carbapenems retained relatively better activity. Early detection and appropriate antibiotic selection are essential to reduce complications and improve patient outcomes.
Order of Authors:	Marwa S Ibrahim, Ph.D
	Ibrahim A. Naqid, Ph.D
Response to Reviewers:	Dear Editor,<br>We are pleased to submit the revised version of our manuscript entitled “Prevalence and Associated Risk Factors of Pseudomonas aeruginosa Infection in Diabetic Wounds in Duhok Province, Iraq.”

Powered by Editorial Manager® and ProduXion Manager® from Aries Systems Corporation

	We would like to thank the reviewers and editorial team for their valuable comments and constructive suggestions, which have significantly improved the quality of our manuscript. We have carefully addressed all comments, and all revisions and changes have been highlighted in red color in the revised manuscript for ease of review.<br>We hope that the revised version meets the journal's requirements and is suitable for publication.<br>Sincerely,<br>Dr Ibrahim Naqid<br><br>Reviewer #1:<br>1.The consent form that was supposed to be written was not part of the submission, even though it was mentioned in the manuscript.<br>Thank you for your comment. We apologize for the oversight regarding the consent form. The written informed consent, which was mentioned in the manuscript, has now been uploaded to the submission system as required.<br><br>2.The English language needs substantial revision. Pseudomonas aeruginosa must consistently be typed in Italic font.<br>Thank you for your comment. We have thoroughly revised the manuscript to improve the English language quality throughout the text. In addition, Pseudomonas aeruginosa has been consistently formatted in italic font in all instances, as required.<br><br>3.The classification of woutnd infection severity in the methodology section requires further clarification. What criteria were used to classify the severity, including the count and percentage?<br>Thank you for your comment. We have clarified the classification of wound infection severity in the methodology section. The severity was defined based on the degree of tissue involvement and clinical presentation, including ulcers (necrosis with erythema, pain, and discharge), abscess formation (pus collection), cellular necrosis (tissue death due to hypoxia or vascular occlusion), and gangrene (advanced necrosis with or without systemic symptoms). The count and percentage of each category have been clearly reported in Table 3.<br>4.Table 3 presents the findings, however, the column headings do not describe the significance of the numbers presented, specifically which number indicates the sample size and which represents the % ?<br>Thank you for your comment. We have revised Table 3 to improve clarity. The column headings have been updated to clearly distinguish the sample size (n) and percentage (%), ensuring that each value is correctly identified and easily interpreted.<br><br>5.What is the significance of (*) in the P-value column of table 3?<br>Thank you for your comment. The asterisk () in the P-value column of Table 3 indicates a statistically significant result ($p < 0.05$). This clarification has now been added in the footnote of Table 3 as " = statistically significant difference" to ensure clear interpretation.<br><br>6.What is the significance of the PCR amplification in this study? No discussion of the findings in the context of the manuscript<br>Thank you for your comment. The required changes added in the first paragraph of discussion. PCR amplification of the 16S rDNA gene was used in this study to confirm the identification of Pseudomonas aeruginosa with high specificity and accuracy. This molecular method strengthens the reliability of the conventional biochemical and Vitek®2 identification results by reducing the possibility of misidentification. Therefore, PCR confirmation supports the validity of the reported prevalence and ensures accurate detection of P. aeruginosa in diabetic wound infections.<br><br>7.The discussion should not reiterate the results; it should analyze and interret the findings in relation to the existing published data in the literature.<br>Thank you for your comment. We have revised the Discussion section by removing repeated results and ensuring that it focuses on interpretation and comparison of findings with relevant published literature. All redundant result descriptions have been deleted and the section has been restructured to improve analytical depth and alignment with standard discussion format.<br><br>8.The conclusion should recommend measures to be taken to address the problems

Powered by Editorial Manager® and ProduXion Manager® from Aries Systems Corporation

raised in the research questions.
Thanks. The required changes have been added in the conclusion section.
Pseudomonas aeruginosa is a significant pathogen in diabetic wound infections with high multidrug resistance, while most sociodemographic and clinical factors showed no strong independent association. The findings highlight the importance of early detection and proper management of diabetic wounds, particularly in high-risk anatomical sites such as the leg region. Strengthening infection control practices, promoting rational antibiotic use, and improving patient education on wound care are essential to reduce complications and antimicrobial resistance in diabetic patients.

9. What are the limitations associated with the current study?
Thank you for your comment. We have addressed this point by clearly stating the study limitations in the Discussion section. These include the cross-sectional design, which limits causal inference, the single-province setting, and the relatively small sample size, which may affect generalizability.

9.Reviewer #2: I. Summary of the Research
This manuscript investigates the prevalence, associated risk factors, and antimicrobial susceptibility patterns of Pseudomonas aeruginosa in diabetic wound infections in Duhok Province, Iraq. A notable strength of this work is its departure from the standard focus on diabetic foot ulcers (DFUs) to include various anatomical sites, which provides a more comprehensive understanding of the pathogen's impact. The study utilizes a multi-pronged identification approach, including molecular confirmation via 16S rDNA PCR, which enhances the analytical validity of the findings.

Thank you for your thoughtful and constructive evaluation of our manuscript. We appreciate your recognition of the study’s broader scope beyond diabetic foot ulcers, as well as the inclusion of multiple anatomical sites to provide a more comprehensive understanding of Pseudomonas aeruginosa infections in diabetic wounds.
Your positive feedback is highly valued and encourages us to further refine and strengthen our work.

However, the manuscript contains a critical temporal error and several major methodological and statistical deficiencies that must be addressed to ensure scientific integrity and reproducibility.

II. Major Revisions (Critical)
1.Fundamental Temporal Inconsistency
Issue: The study period is reported as being in the future (“July 2024 to July 2025”) in the Abstract (Line 35) and the Methods (Line 112). This discrepancy suggests the results are hypothetical or simulated, which severely undermines the credibility of the entire paper.
Recommendation: Immediately correct the dates to reflect the actual, past timeframe of data collection. This is a non-negotiable requirement for truthful reporting.
Thank you for this important comment. We apologize for the error in reporting the study period. The dates have been corrected to reflect the actual data collection timeframe, and all inconsistencies in the Abstract and Methods sections have been revised accordingly to ensure accurate and consistent reporting throughout the manuscript.

2.Statistical Rigor and Multivariable Analysis
Issue: The statistical analysis is underdeveloped, appearing only in Lines 187. The authors list t-tests and Chi-square tests but omit any multivariable analysis (e.g., binary logistic regression). Without controlling for confounders, the claims regarding "associated risk factors" are not robustly supported.
Recommendation: Conduct a multivariable analysis to identify independent risk factors and report adjusted odds ratios (AORs) with 95% confidence intervals (CIs).
Thank you for your comment. We have performed a full reanalysis using appropriate multivariable logistic regression to control for potential confounders. The results have been updated throughout the manuscript, and adjusted odds ratios (AORs) with 95% confidence intervals (95% CIs) are now reported. In addition, the Discussion section has been revised accordingly to reflect the findings of the adjusted analysis and to ensure accurate interpretation of independent risk factors.

Powered by Editorial Manager® and ProduXion Manager® from Aries Systems Corporation

	3.Sample Size Justification<br>Issue: The manuscript mentions the sample size of 141 in Line 113, but provides no justification for this number. Without a power analysis, it is unclear if the study was adequately powered to detect significant associations, such as those for BMI and wound site.<br>Recommendation: Provide a detailed sample size calculation or a retrospective power analysis in the Methods section.<br><br>Thank you for your valuable comment. This has been addressed and the sample size justification has been added in the Methods section. The sample size of 141 was determined based on all eligible diabetic wound infection cases admitted during the study period in selected hospitals. Therefore, a census sampling approach was used rather than a calculated sample size.<br><br><br>4. Data Availability and Transparency<br>Issue: The statement that “All relevant data are within the manuscript and its Supporting Information files” is misleading because there are no Supporting Information files included in the submission.<br>Recommendation: Upload the anonymized dataset as per the journal’s format (e.g. as Supplementary Files).<br>Thank you for your comment. We apologize for the confusion. The data availability statement has been revised accordingly, and the anonymized dataset will be uploaded as Supplementary Files in the required journal format.<br><br>III. Specific Comments & Technical Points<br>Results and Logical Consistency<br>- BMI Misinterpretation: The text in Line 209 (Results) and Line 325 (Discussion) incorrectly implies that obese individuals had the highest "rate" of P. aeruginosa infection. However, Table 2 (Line 233) clearly shows Normal weight individuals had the highest rate (58.33%) within their group. Revise the narrative to match the data.<br>Thank you for your careful observation, and we apologize for the confusion caused by this inconsistency. Although obesity was the most prevalent BMI category among diabetic patients in the study population, the highest proportion of Pseudomonas aeruginosa diabetic wound infections (PsA DWIs) was observed in the normal-weight group (58.33%).<br>- Unsupported Conclusions: The conclusion in Line 352 states that the highest rates were among the "uneducated". This contradicts Line 217, which explicitly states the association with educational level was not statistically significant (P = 0.2933).<br>Thank you for your careful review, and we apologize for the confusion. We have revised the conclusion accordingly to ensure consistency and accuracy<br><br><br>- Clarity and Style<br>Redundancy: Remove the redundant phrasing ("widespread epidemic" repeated) in Line 64. Thank you for your comment. The redundant phrasing (“widespread epidemic”) in Line 64 has been removed as suggested.<br><br>Standardization: Ensure "P. aeruginosa" is formatted consistently throughout.<br>Thank you for your comment. The formatting of P. aeruginosa has been standardized consistently throughout the manuscript.<br><br>Sentence Structure: Simplify the convoluted description of wound locations in Line 246 and treatment types in Line 265.<br>Thank you for your comment. The sentence structure describing wound locations and treatment types has been simplified for clarity<br><br>- Reproducibility Details:<br>Sampling: Explicitly state if consecutive, random, or convenience sampling was used in Line 112.<br>We have clarified the sampling method in the Methods section<br>CLSI version: Specify the exact version/year of the CLSI guidelines used in Line 149.<br>Thank you for your comment. The exact version/year of the Clinical and Laboratory Standards Institute (CLSI) guidelines

Powered by Editorial Manager® and ProduXion Manager® from Aries Systems Corporation

	PCR Master Mix: Provide the manufacturer and catalog number for the master mix in Line 164.<br>Thank you for your comment. The manufacturer and catalog number of the PCR master mix have been added as requested
Additional Information:	
Question	Response
**Financial Disclosure**<br><br>Enter a financial disclosure statement that describes the sources of funding for the work included in this submission. Review the submission guidelines for detailed requirements. View published research articles from *PLOS ONE* for specific examples.<br><br>This statement is required for submission and **will appear in the published article** if the submission is accepted. Please make sure it is accurate.<br><br>**Funded studies**<br>Enter a statement with the following details:<br>• Initials of the authors who received each award<br>• Grant numbers awarded to each author<br>• The full name of each funder<br>• URL of each funder website<br>• Did the sponsors or funders play any role in the study design, data collection and analysis, decision to publish, or preparation of the manuscript?<br><br>Did you receive funding for this work?	The author(s) received no specific funding for this work.
**Competing Interests**<br><br>Use the instructions below to enter a competing interest statement for this submission. On behalf of all authors, disclose any competing interests that could be perceived to bias this work—acknowledging all financial support and any other relevant financial or non-financial competing interests.	The authors have declared that no competing interests exist.

This statement is required for submission and **will appear in the published article** if the submission is accepted. Please make sure it is accurate and that any funding sources listed in your Funding Information later in the submission form are also declared in your Financial Disclosure statement.<br><br>View published research articles from *PLOS ONE* for specific examples.<br><br>**NO authors have competing interests**<br><br>Enter: *The authors have declared that no competing interests exist.*<br><br>**Authors with competing interests**<br><br>Enter competing interest details beginning with this statement:<br><br>*I have read the journal's policy and the authors of this manuscript have the following competing interests: [insert competing interests here]*<br><br>* typeset	
**Data Availability**<br><br>Authors are required to make all data underlying the findings described fully available, without restriction, and from the time of publication. PLOS allows rare exceptions to address legal and ethical concerns. See the PLOS Data Policy and FAQ for detailed information.<br><br>A Data Availability Statement describing where the data can be found is required at submission. Your answers to this question constitute the Data Availability Statement and **will be published in the article**, if accepted.	Yes - all data are fully available without restriction

Powered by Editorial Manager® and ProduXion Manager® from Aries Systems Corporation

**Important:** Stating 'data available on request from the author' is not sufficient. If your data are only available upon request, select 'No' for the first question and explain your exceptional situation in the text box.<br><br>Do the authors confirm that all data underlying the findings described in their manuscript are fully available without restriction?	
Additional data availability information:	Tick here if the URLs/accession numbers/DOIs will be available only after acceptance of the manuscript for publication so that we can ensure their inclusion before publication.
**Ethics Statement**<br><br>Enter an ethics statement for this submission. This statement is required if the study involved:<br><br>• Human participants<br>• Human specimens or tissue<br>• Vertebrate animals or cephalopods<br>• Vertebrate embryos or tissues<br>• Field research<br><br>Write "N/A" if the submission does not require an ethics statement.<br><br>General guidance is provided below. Consult the submission guidelines for detailed instructions. **Make sure that all information entered here is included in the Methods section of the manuscript.**	Ethical approval for this study was obtained from the Ethical Committee for Medical and General Health Research Ethics from the Ministry of Health/ Duhok Directorate General of Health, under the reference number (29052024-4-2). Written informed consent was obtained from all participants before sampling

Powered by Editorial Manager® and ProduXion Manager® from Aries Systems Corporation

**Format for specific study types**<br><br>**Human Subject Research (involving human participants and/or tissue)**<br>• Give the name of the institutional review board or ethics committee that approved the study<br>• Include the approval number and/or a statement indicating approval of this research<br>• Indicate the form of consent obtained (written/oral) or the reason that consent was not obtained (e.g. the data were analyzed anonymously)<br><br>**Animal Research (involving vertebrate animals, embryos or tissues)**<br>• Provide the name of the Institutional Animal Care and Use Committee (IACUC) or other relevant ethics board that reviewed the study protocol, and indicate whether they approved this research or granted a formal waiver of ethical approval<br>• Include an approval number if one was obtained<br>• If the study involved *non-human primates*, add *additional details* about animal welfare and steps taken to ameliorate suffering<br>• If anesthesia, euthanasia, or any kind of animal sacrifice is part of the study, include briefly which substances and/or methods were applied<br><br>**Field Research**<br><br>Include the following details if this study involves the collection of plant, animal, or other materials from a natural setting:<br>• Field permit number<br>• Name of the institution or relevant body that granted permission	
Data Availability<br><br>Before publication, Authors are required to make fully available and without restriction all data underlying their findings. Sharing the data underlying your findings increases the visibility, credibility, and impact of your work, while supporting transparency and future scientific discovery. Please see our PLOS Data Policy page for detailed information on this policy.</p><br>A Data Availability	All relevant data are within the manuscript and its Supporting Information files.

Statement, detailing where the data can be accessed, is required at first submission. Insert your Data Availability Statement in the box below.</p> Please see the data reporting of our submission guidelines for instructions on what you need to include in your Data Availability Statement. Examples of acceptable Data Availability Statements are included here for reference:</p> - The minimal data set [and accompanying code, where generated] is available at [repository name] via [DOI hyperlink] - No data was generated by this study. The following existing data sources were used: [dataset name] from [dataset location] available via [web address or DOI hyperlink]. - For Study Protocols: No datasets were generated or analysed during the current study. All relevant data from this study will be made available upon study completion. <i>PLOS allows rare exemptions to address legal and ethical concerns. If you have legal or ethical restrictions, please use the box below to detail these in full sentences for the Journal team to consider.</i></p> This statement will be typeset if the manuscript is accepted for publication.</p>	

Manuscript

Click here to access/download;Manuscript;Manusceipt-Pseudomonas research 21-12-2025.docx

Prevalence and associated risk factors of *Pseudomonas aeruginosa* infection in Diabetic wounds in Duhok province, Iraq

Marwa S. Ibrahim[1] and Ibrahim A. Naqid[2*]

[1]Department of Medical Laboratory Technology, College of Health and Medical Techniques-Shekhan, Duhok Polytechnic University, Duhok, Kurdistan Region, Iraq

Department of Biomedical Sciences, College of Medicine, University of Zakho, Kurdistan Region, Iraq

Abstract

Background and aims

Diabetes mellitus is a chronic metabolic disorder and a major global health problem. Individuals with diabetes are at increased risk of wound infections, with *Pseudomonas aeruginosa* being a common causative pathogen due to its virulence and antibiotic resistance. This study aimed to isolate *P. aeruginosa* and assess associated risk factors and antibiotic susceptibility patterns in diabetic wound infections across hospitals in Duhok Province, Kurdistan Region, Iraq.

Methods

This cross-sectional study was conducted from July 2024 to July 2025 in Duhok Province, Kurdistan Region, Iraq. A total 141 wound swab samples were collected from diabetic patients aged ≥18 years. Samples were cultured on standard media, and *Pseudomonas aeruginosa* was identified using conventional biochemical tests, the Vitek®2 system, and molecular confirmation by 16S rDNA PCR. Antimicrobial susceptibility testing was performed using the disc diffusion method on Mueller–Hinton agar. Data were analyzed using GraphPad Prism software.

Results

Out of 141 wound infection samples collected from diabetic patients, *Pseudomonas aeruginosa* was isolated in 44.68% of cases. Among the sociodemographic and clinical variables, body mass index and anatomical site of the wound were significantly associated with PsA diabetic wound infections ($p = 0.0277$ and $p = 0.0253$, respectively). All isolates were confirmed by 16S rDNA PCR. Antimicrobial susceptibility testing showed highest sensitivity to colistin (63%) and meropenem (59%), while high resistance was observed to most other antibiotics.

Conclusion

Pseudomonas aeruginosa is a common cause of wound infections in patients with diabetes mellitus, with its prevalence significantly associated with body mass index and wound site. The high level of antibiotic resistance presents a major therapeutic challenge. Early detection and appropriate antibiotic selection are essential to reduce complications and improve patient outcomes.

Keywords:

Pseudomonas aeruginosa; Diabetic wound infection; Prevalence; Risk factors; Antibiotic susceptibility; Duhok Province; Iraq

Introduction

Diabetes mellitus (DM) is a chronic metabolic disorder characterized by elevated blood glucose levels and disturbance in the metabolism of carbohydrates, lipids profile leading to insulin resistance [1]. It is also recognized as a major public health concern and a widespread epidemic, leading to disability and a widespread global epidemic, contributing substantially to disability in both developing and developed countries[1, 2]. DM is classified into type 1 and type 2 diabetes [3]. Patient with type 2 diabetes are at increased risk of developing multiple complications, including hypertension, cardiovascular disease, renal failure, cerebrovascular accident, retinopathy and neuropathy[4, 5]. In addition to comorbidities, several other factors can influence the risk and severity of infection. These include local conditions such as poor tissue perfusion, the presence of foreign bodies, necrosis, wound location and size, poorly controlled diabetes. Behavioural determinants such as malnutrition, non-adherence to treatment plan, and smoking; as well as social determinants such as educational level and socioeconomic status, also play a significant role [6]. Furthermore, peripheral ischemic disease is a frequent and severe complication that can lead to ulceration and wound infection, especially diabetics foot ulcer (DFU) [1]. Ulceration represents the most common clinical manifestation of wound infections, followed by cellulitis and gangrene. The dorsum of the foot is the most frequent site of infection, followed by the forefoot and toes [7]. This complication may be further progress to osteomyelitis [8]. Among DM patients who develop foot ulcers, approximately 20–30% require minor amputation, whereas about 5% undergo major amputation [6].

All types of chronic wound infections contain various microorganisms, and microbial colonization of a localized wound can lead to extensive replication on the wound surface. As the microbial load increases, pathogens may penetrate from superficial layers into deeper tissues, , resulting in the clinical signs and symptoms among DM patients [6]. Both gram-positive and gram-negative bacteria causes wound infection, and *Pseudomonas aeruginosa* (*P. aeruginosa*) considered as one of the most common pathogenic bacteria among DM patients[9, 10]. *P. aeruginosa* can cause serious infection in multiple organs, including pneumonia, urinary tract infections, keratitis and soft tissue infections associated with open wounds leading to acute damage to tissue, particularly diabetics foot ulcers and burn injuries [11]. This pathogen is able to produce various toxins, evade phagocytosis, and exhibits a broad spectrum of antibiotic resistance, making it a highly dangerous and challenging organism to manage [2]. The previous study conducted on *P. aeruginosa* in diabetic foot infection (DFI) in Erbil, Iraq showed a prevalence of (35.33%) [11]. Another study, conducted on Erbil [12] found *P. aeruginosa* to be the most common gram-negative bacterium, at 21.43%. Meanwhile, a study conducted by [13] in Sulaymaniyah showed that the rate of *P. aeruginosa* infection among DFIs was 8.5% .

Antibiotic resistance in *P. aeruginosa* has become a major global public health concern, particularly due to the limited availability of effective alternative treatment. Infections caused by this pathogen significantly contribute to morbidity and mortality worldwide. According to the European Centers for Disease Prevention and Control indicated, 13.7% of *P. aeruginosa* isolates demonstrate resistant to at least three antimicrobial groups, while 5.5% show resistance to all five antimicrobial groups under

surveillance [14]. The declining sensitivity of *P. aeruginosa* to commonly used antibiotics underscores the urgent need to prevent antibiotic overuse and to strengthen antimicrobial stewardship efforts, which may help curb resistance in the future [10]. Furthermore, antibiotic selection for the treating aeruginosa infections is often inconsistent, highlighting the critical importance of choosing appropriate, evidence-based therapeutic regimens [11].

Most previous studies in the Kurdistan Region of Iraq have primarily focused on *Pseudomonas aeruginosa* in diabetic foot ulcers. Therefore, the present study aimed to isolate *P. aeruginosa* from wound infections occurring at various anatomical sites among diabetic patients across different hospitals in Duhok Province, Kurdistan Region, Iraq. Additionally, the study sought to identify major risk factors associated with *P. aeruginosa* wound infections and to determine the antibiotics to which these isolates are most susceptible.

Material and method

Study design, and sample collection

This cross-sectional study was conducted in Duhok Province, Kurdistan Region, Iraq, from July 2024 to July 2025. A total of 141 wound samples were collected from diabetic outpatients (77 females, 64 males) aged ≥18 years at Zakho General Hospital and Azadi Teaching Hospital. Wounds were first cleaned with normal saline, and then samples were obtained using sterile swabs and transported in Amies medium. All samples were cultured on appropriate media at the Microbiology Laboratory of Zakho Technical Institute. Written informed consent was obtained from all participants before sampling

Severity of wound infection:

The severity of wound infection was classified based on the degree of tissue involvement. Poor skin care and unrecognized skin injuries may progress to chronic ulcers, facilitating microbial invasion of skin tissue. As infection extends to deeper layers, it may involve muscles, joints, and tendon sheaths, leading to pus accumulation, tissue necrosis, and abscess formation. Diabetic ulcers are open skin wounds characterized by necrosis, surrounding erythema, pain, and purulent discharge [16]. Necrosis may also result from vascular occlusion or trauma, causing tissue hypoxia and cell death. Advanced necrosis leads to eschar formation and may progress to wet or dry gangrene, often accompanied by systemic symptoms such as fever and malaise. Wet gangrene is frequently associated with foul odor due to secondary infection [17]

Body Mass Index (BMI)

Patients' weight and height were measured, and body mass index (BMI, kg/m^2) was calculated and classified as underweight (<18.5), normal weight (18.5–24.9), overweight (25.0–29.9), or obese (≥30.0).[15].

Bacterial Isolation and Identification

Wound swab samples were immediately cultured on Blood, MacConkey, and Chocolate agars and incubated overnight at 37 °C. Suspected Pseudomonas aeruginosa colonies were selected and sub-cultured on MacConkey agar to obtain pure isolates. Further characterization was performed using biochemical tests, including peptone water, methyl red, Voges–Proskauer, Simmons citrate, Triple Sugar Iron (TSI), cetrimide agar, oxidase test, and Gram staining [18]. All isolates were additionally confirmed using the automated Vitek®2 system (bioMérieux, France).

Antibiotic susceptibility test

Antimicrobial susceptibility testing of *P. aeruginosa* isolates was performed using twelve antibiotic discs (Bioanalyses, Turkey). The disc diffusion method was performed on Mueller-Hinton agar to detect antibiotic susceptibility and resistance. After 24 hours of incubation, the diameters of the inhibition zone around each disc were measured, and compared to standard reference value. Based on on the size of the inhibition zones isolates were interpreted as susceptible or resistant, in accordance with clinical laboratory standards recommendations [19].

Molecular investigation

Genomic DNA extraction

Genomic DNA of all isolated *P. aeruginosa strains* was extracted, using a commercial extraction kit (PrimePrepTM Genomic DNA Extraction Kit) from GeNet Bio (Korea). Following extraction, the concentration and purity of the DNA samples were determined using a NanoDrop spectrophotometer (Thermo scientific, USA). The extracted genomic DNA was consequently used as a template for polymerase chain reaction (PCR) amplification. All procedure was conducted at the Research Center of the Biology Department, Faculty of Science, University of Zakho.

Primers

The 16S rDNA specific species primer set was used for the molecular identification of the 16S rDNA gene in *P. aeruginosa*. The primer sequences used were: forward (GGGGGATCTTCGGACCTCA) and reverse (TCCTTAGAGTGCCCACCCG), yielding an expected amplicon size of 956 base pairs as described previously [20]. A positive control for the 16S rDNA gene was obtained from [21] with the accession No. (OR882879).

PCR amplification and gel electrophoresis

The final volume of the PCR amplification reaction was 20 µl consisting of 8 µL of master mix**,** 2 µL of forward primer**,** 2 µL of reverse primer**,** 2 µL of genomic DNA (25–50 ng/µL)**,** and 8 µL of PCR-grade water. Following preparation of the reaction mixture, the tubes were placed into the thermal cycler for amplification. The amplification conditions are summarized in Table (1). After amplification, the PCR products were analysed by gel electrophoresis. A 1.25% (w/v) agarose gel was prepared using 1× Tris–Borate–EDTA (TBE) buffer**,** and samples were electrophoresed for 5 minutes at 45 V, followed by 30 minutes at 80 V. DNA bands were subsequently visualized under a UV transilluminator (Cleaver

Scientific, UK). The procedure followed the standard protocols described by Ausubel and his colleagues [22].

Table 1: The amplification condition of 16S rDNA PCR analysis for 16S rDNA primer

Initial denaturation	Denaturation	Annealing	Extension	Final extension	Reference
94 ºC	92 ºC	54 ºC	72 ºC	72 ºC	[20]
2 min	20 sec	20 sec	40 sec.	5 min	
1 cycle	25 cycles			1 cycle	

Inclusion and exclusion criteria

All clinical samples obtained from diabetic patients aged ≥ 18 years who met the diagnostic criteria for wound infection and were admitted to hospitals and clinical outpatients' settings were included in the study. The exclusion criteria were based on three main factors: (1) patients younger than 18-years of age, (2) patients who declined participation in the study and (3) patient who had received antimicrobial therapy prior to sample collection.

Ethics

Ethical approval for this study was obtained from the Ethical Committee for Medical and General Health Research Ethics from the Ministry of Health/ Duhok Directorate General of Health, under the reference number (29052024-4-2).

Statistical analysis

GraphPad Prism 8.1 software were used for statistical analysis. The *t*-test and Chi-square test were used to evaluate statistically significant differences between categorical variables. A $p < 0.05$ was considered statistically significant.

Result

Demographic characteristics of the diabetic patients with wound infection

A total of 141 patients were included in the analysis, *Pseudomonas aeruginosa*–associated diabetic wound infections (PsA DWIs) were identified in 63 patients (44.68%), whereas non-PsA DWIs were detected in 78 patients (55.32%). The sociodemographic characteristics of patients with diabetes wound infection with *P. aeruginosa* and without *P. aeruginosa* is presented in Table (2).

Sex distribution did not differ significantly between the two groups (p=0.3772). Females represented 54.61% of the total sample, and their proportions were comparable between non-PsA (51.95%) and PsA DWIs (48.05%).

Most participants with DM wound infection include in this study, who resided in urban areas rather than rural areas, at rates of 78.01% and 21.99%, respectively. The difference between groups was not statistically significant (P = 0.6384). In both urban and rural areas, PsA DWIs was lower than non-PsA DWIs.

Among all DM patients, obese individual had the highest BMI (51.06%), with an equal incidence of wound infection with PsA DWIs and those without. This was followed by overweight (31.91%) and normal weight (17.02). The rates of PsA DWIs were 58.33% and 28.89% for normal and overweight individuals, respectively, while the rates of non-PsA DWIs were 41.67% and 71.11% for normal and overweight individuals, respectively. Body mass index (BMI) was significantly associated with PsA status (P = 0.0277). The educational level varied, with 59.57% being illiterate, 21.48% having Primary school, 13.48% having Secondary and Tertiary school, 5.67% having High educational, and in all cases the incidence of PsA DWIs were lesser or equal to the incidence of non-PsA DWIs. The association between educational level and PsA DWIs was not statistically significant (P = 0.2933).

Nearly a three-quarter (73.76%) of the DWI were unemployed. This included, housewife female (51.77%) and unemployed male (21.99), followed by 10.64% of government employees, 9.93% daily labor, and 5.67% pension. In all cases, a higher rate of PsA DWIs was found only among the unemployed with rate of (51.61%), while the distribution of PsA and non-PsA DWIs was equal (50%) in other categories. The association between occupational status and PsA DWIs was not statistically significant (P = 0.0765).

The rates of non-smoker among patients were higher than former and current smokers with (65.96%, 17.02%, 17.02%, respectively), also among non-smoker, non-PsA DWIs was higher than PsA DWIs (52.69% vs 47.31%). Moreover, patients with PsA DWIs were also less likely to be former and current smokers than non-PsA DWIs (33.33%, 45.83% vs 66.67%, 54.17%). The association between smoking status and PsA DWIs was not statistically significant (P = 0.4668).

The number of patients with and without a family history of diabetes was nearly similar (74 vs. 67). In both groups, the prevalence of PsA DWIs was slightly lower than that of non-PsA DWIs. This difference was not statistically significant (P = 0.3193).

Table 2: Sociodemographic characteristics of diabetes wound patients with *P. aeruginosa* and without *P. aeruginosa* infection

**Characteristic**	**Total No. (%) = 141 (100))**	**Non- PsA DWIs (No. (%) = 78 (55.32))**	**PsA DWIs (No. (%) = 63 (44.68))**	**P Value**
**Sex**				
Female	77 (54.61)	40 (51.95)	37 (48.05)	0.3772
Male	64 (45.39)	38 (59.38)	26 (40.63)	
**Age Year (Mean** ±SD)	57.48 ± 10.66	57.26 ± 10.52	57.76 ± 10.91	0.7592
**Age Group (Year)**				
18-49	29 (20.57)	17 (58.62)	12 (41.38)	
50-59	45 (31.91)	25 (55.56)	20 (44.44)	0.9699
60-69	42 (29.79)	23 (54.76)	19 (45.24)	
≥70	25 (17.73)	13 (52)	12 (48)	
**Region**				
Urban	110 (78.01)	62 (56.36)	48 (43.64)	0.6384
Rural	31 (21.99)	16 (51.61)	15 (48.39)	
**BMI (Mean** ±SD)	30.28 ± 5.82	29.80 ± 4.99	30.88 ± 6.69	
**BMI (kg/m^2)**				
Normal	24 (17.02)	10 (41.67)	14 (58.33)	0.0277 *
Overweight (Pre-obesity)	45 (31.91)	32 (71.11)	13 (28.89)	
Obesity	72 (51.06)	36 (50)	36 (50)	
**Educational Level**				
Non-educational (illiterate)	84 (59.57)	42 (50)	42 (50)	
Primary school	30 (21.48)	21 (70)	9 (30)	0.2933
Secondary and Tertiary school	19 (13.48)	11 (57.89)	8 (42.11)	
High educational (Institute or college)	8 (5.67)	4 (50)	4 (50)	
**Occupational status**				
Daily Labor	14 (9.93)	7 (50)	7 (50)	
Government Employee	15 (10.64)	12 (80)	3 (20)	
Pension	8 (5.67)	7 (87.5)	1 (12.5)	0.0765
Housewife	73 (51.77)	37 (50.68)	36 (49.32)	
Unemployed	31 (21.99)	15 (48.39)	16 (51.61)	
**Smoking**				
Non smoker	93 (65.96)	49 (52.69)	44 (47.31)	0.4668
Former smoker	24 (17.02)	16 (66.67)	8 (33.33)	
Current smoker	24 (17.02)	13 (54.17)	11 (45.83)	
**Family History of DM**				
**Yes**	74 (52.48)	38 (51.35)	36 (48.65)	0.3193
**No**	67 (47.52)	40 (59.7)	27 (40.3)	

Note: **PsA** = *Pseudomonas aeruginosa,* **DWIs** = Diabetes Wound Infections

The clinical characteristics of diabetic patients with PsA DWIs and non-PsA DWIs

The clinical characteristics of PsA DWIs and non-PsA DWIs are illustrated in Table (3). The sample of diabetic patients with a glycated hemoglobin (HbA1C) level between 4 and 6.9 included 14 samples, while the sample with levels between 7 and 16 included 127 samples. The overall mean and standard deviation (SD) of HbA1C level was 9.23 ± 2.18, and HbA1C mean was higher in non-PsA DWIs cases compared to PsA DWIs (9.25 ± 1.98 vs 9.20 ± 2.43) and the difference was not statistically significant (P = 0.9016).

The mean duration of diabetics were 13.86 ± 7.72 years for patients with PsA DWIs and 13.97 ± 8.99 years for those with non-PsA DWIs, with no statistically significant difference (P = 0.9345)., Similarly, the history of wound infection (in months) did not differ significantly between groups (p=0.1468), with a higher mean duration observed in non-PsA DWI cases (13.13 ± 38.41 months) compared to PsA DWI cases (5.82 ± 11.34 months). Patients with PsA DWIs and non-PsA DWIs were more commonly had leg and foot site infection (49.55% vs 50.45%), with a totally rates of 78.72%, while the rate in other parts of the body was 21.28%, which included 26.67% with *Pseudomonas* infection and 73.33% without *Pseudomonas* infection. This difference was statistically significant (P = 0.0253).

Among all DWIs cases, wet wounds were the most common (60.99%), followed by dry wounds (35.46%) and mixed wounds (3.55%). In wet wounds, the proportion of PsA DWIs was equal to non-PsA DWIs (50% each). In dry and mixed wounds, PsA DWIs were observed in 39% and 40% of cases, respectively, whereas non-PsA DWIs accounted for 64% and 60%, respectively. The differences were not statistically significant (P = 0.279).

On the other side, the high rate of PsA DWIs was observed in ulcer with cellular necrosis and gangrene with PsA accounting for 54.55% of cases compared to 45.45% for non-PsA DWIs For other wound types, the proportion of PsA DWIs was equal to or lower than that of non-PsA DWIs: 36.36% for abscesses, 33.33% for cellular necrosis, 50% for gangrene, 37.84% for ulcers, and 47.95% for ulcers with cellular necrosis. The differences were not statistically significant (P = 0.8517). Approximately 112 patients (90.76%) with DWIs had additional chronic diseases, with eye problems being the most common with 66 (21.2%) cases. Among the comorbid disease, PsA DWIs were more frequent in patients with periodontal disease, and hypertension at rates of 51.61 and 48.31, respectively, while the incidence of other comorbid diseases was high among DM patients with non-PsA DWIs The association between comorbidities and PsA DWIs was not statistically significant (P = 0.7481).

Regarding their treatment methods, 44 (31.12%) of DM used both diets and oral hypoglycemic tablets, followed by diets and insulin at 33 (23.4%). In contrast, the highest rate of the treatment types among PsA DWIs was insulin and oral hypoglycemic tablets at 66.67%, followed by diets and oral hypoglycemic tablets, and insulin alone at 50% and 50% respectively, the differences in treatment methods between PsA and non-PsA DWIs were not statistically significant (P = 0.7061). Regarding physical activity, the majority of patients (90.07%) did not engage in regular exercise, while only 9.93% reported exercising.

In both cases, non-PsA DWIs were more prevalent than PsA DWIs, with no statistically significant association observed (P =0.4771).

The use of wound dressing and antiseptics among diabetic patients with wound infection was high at 91.49% and 92.2%, respectively, compared with those who did not use these interventions. In both cases, the prevalence of PsA DWIs was lower than that of non-PsA DWIs, and the differences were not statistically significant.

Table 3: clinical characteristics of diabetes wound patients with *P. aeruginosa* and without *P. aeruginosa* infection

Characteristic	Total Population	Non- PsA DWIs	PsA DWIs	P Value
**HBA1C (Mean ±SD)**	9.23 ± 2.18	9.25 ± 1.98	9.20 ± 2.43	0.9016
**Duration of DM (Mean ±SD)**	13.92 ± 8.42	13.97 ± 8.99	13.86 ± 7.72	0.9345
**History of ulcer (Wound infection)**	9.86 ± 29.29	13.13 ± 38.41	5.82 ± 11.34	0.1468
**Location of wound infection (skin ulcer):**				0.0253 *
Non-Leg region	30 (21.28)	22 (73.33)	8 (26.67)	
Leg region (Leg and Foot)	111 (78.72)	56 (50.45)	55 (49.55)	
**Type of Wound infection**				0.279
Dry	50 (35.46)	32 (64)	18 (36)	
Wet	86 (60.99)	43 (50)	43 (50)	
Both	5 (3.55)	3 (60)	2 (40)	
**Degree of Wound infection (Lesion Severity) (Clinical presentations)**				0.8517
Abscess	11 (7.8)	7 (63.64)	4 (36.36)	
Cellular death	3 (2.13)	2 (66.67)	1 (33.33)	
Gangrene	6 (4.26)	3 (50)	3 (50)	
Ulcer	37 (26.24)	23 (62.16)	14 (37.84)	
Ulcer with Cellular Death	73 (51.77)	38 (52.05)	35 (47.95)	
Ulcer with Cellular death and Gangrene	11 (7.8)	5 (45.45)	6 (54.55)	
**Comorbid condition**				
Hypertension	89 (25)	46 (51.69)	43 (48.31)	0.7481
Cardiac disease	43 (14.13)	26 (60.47)	17 (39.53)	
Renal disease	33 (10.87)	20 (60.61)	13 (39.39)	
Eye problem	66 (21.2)	39 (59.09)	27 (40.91)	
Periodontal (gum) disease	31 (8.15)	15 (48.39)	16 (51.61)	
Other infection	32 (11.41)	21 (65.63)	11 (34.38)	
No other comorbid conditions	29 (9.24)	17 (58.62)	12 (41.38)	
**Types of Diabetic treatment**				0.7061
Diets Only	5 (3.55)	4 (80)	1 (20)	

Insulin Only	4 (2.84)	2 (50)	2 (50)	
Oral Hypoglycemic Tablets Only	19 (13.48)	10 (52.63)	9 (47.37)	
Diets and Insulin	33 (23.4)	20 (60.61)	13 (39.39)	
Diets and Oral Hypoglycemic Tablets	44 (31.21)	22 (50)	22 (50)	
Insulin and Oral Hypoglycemic Tablets	6 (4.26)	2 (33.33)	4 (66.67)	
Diet, Insulin and Hypoglycemic Tablets	25 (17.73)	14 (56)	11 (44)	
No treatment	5 (3.55)	4 (80)	1 (20)	
**Exercise Status**				0.4771
No	127 (90.07)	69 (54.33)	58 (45.67)	
Yes	14 (9.93)	9 (64.29)	5 (35.71)	
**Wound Dressing Applied**				0.8262
No	12 (8.51)	7 (58.33)	5 (41.67)	
Yes	129 (91.49)	71 (55.04)	58 (44.96)	
**Use of Antiseptics for Wound Care**				0.9571
No	11 (7.8)	6 (54.55)	5 (45.45)	
Yes	130 (92.2)	72 (55.38)	58 (44.62)	

Note: **PsA** = *Pseudomonas aeruginosa,* **DWIs** = Diabetes Wound Infections

Antibiotics susceptibility profile

Of the twelve antibiotics tested against 63 *P. aeruginosa* isolates, only two antibiotics had showed high sensitivity: meropenem 37 (59%) and colistin 40 (63%). The remaining antibiotics exhibited higher resistance rates than sensitivity, as presented in Table 4.

Table 4: Antibiotics susceptibility profile among *P. aeruginosa* bacteria

Category	**Antibiotics /µg**	**Resistance No. (%)**	**Sensitive No. (%)**
Monobactam	Aztreonam (ATM 30)	56 (89)	7 (11)
Carbapenem	Imipenem (IPM 10)	33 (52)	30 (48)
	Meropenem (MEM 10)	26 (41)	37 (59)
3rd generation Cephalosporins	Ceftazidime (CAZ 30)	53 (84)	10 (16)
4th generation Cephalosporins	Cefepime (FEP 10)	51 (81)	12 (19)
Aminoglycosides	Amikacin (AK 10)	47 (75)	16 (25)
	Gentamicin (CN 10)	39 (62)	24 (38)
	Tobramycin (TOB 10)	54 (86)	9 (14)
Fluoroquinolones	Ciprofloxacin (CIP 10)	50 (79)	13 (21)
	Norfloxacin (NOR 30)	35 (56)	28 (44)
	levofloxacin (LEV 5)	50 (79)	13 (21)
Polypeptides	Colistin (CT 10)	23 (37)	40 (63)

Molecular identification

All Pseudomonas aeruginosa isolates were confirmed by PCR amplification of the 16S rDNA gene, producing a single band of 956 base pairs (Fig 1).

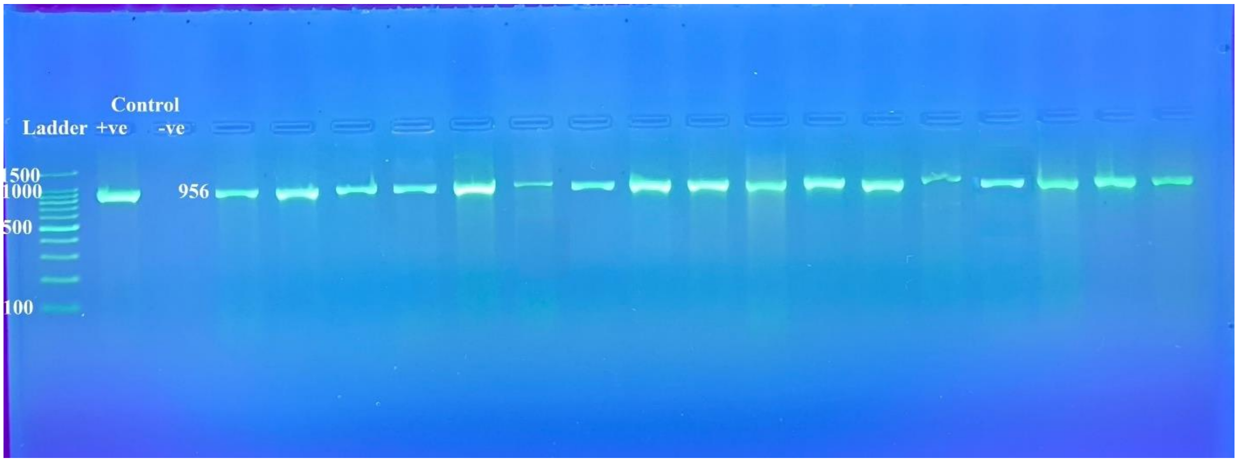

Fig 1: The amplification of PCR for species identification of *P.* aeruginosa using 16S rDNA primer

Discussion

Diabetes mellitus patients represent a major risk factor for the skin wound infections, different microorganisms can cause wound infections, including *P. aeruginosa*. Previous studies on *P. aeruginosa* in DM patients have largely focused on DFIs. In contrast, the present study examined *P. aeruginosa*–associated wound infections across all anatomical sites, with a prevalence of 63 (44.68%) out of 141 patients. In addition to phenotypic identification, all *P. aeruginosa* isolates were molecularly confirmed using the 16S rDNA gene. This finding is consistent with a study conducted in Duhok city, which confirmed all bacterial isolates using the same 16S rDNA gene [23].

The closest prevalence rate to our study, about *P. aeruginosa* among DFU was observed in Najaf city, Iraq (34.4%) [9], in Iran (35%) [24], and in different geographical areas in India, it were (23.7%) in north India, (29.8%) south India, (11.89%) north-east India, (11.7%) east India, and (27%) wear India [25]. Furthermore, a study conducted in Erbil city, Iraq, reported that 53 of 150 DFU samples (35.33%) were infected with *P. aeruginosa* [11]. Moreover, the rates of PsA DWIs was low compared to non-PsA DWIs and the result is different from the previous study, have been conducted in Anhui province-China, in which *P. aeruginosa* among 292 diabetics food ulcer patients was (9%) and non *P. aeruginosa* infection was (91%) [26]. This difference may be due to the variation bacteria causing the infection, changes in the geographical area, and the severity and types of infection [27].

The current study found a higher rates of DM among females compared to males, and a high rate of PsA DWIs have been found among the 50-59 age group. The closet study conducted in Kermanshah

province-Iran, showed a female prevalence of (57.4%) and a male prevalence of (42.6%) [1]. This was in contrasts with a previous study in Najaf city-Iraq, where female was (44.3%) and male was (55.7%), among 282 DM patients with foot wound infection [9]. Furthermore, another study in Iraq was various in which a male to female ratio of 2.5:1, while with a similar age group results, found the highest cases of patients with DFI in the 51-60 age group [28]. The reason behind the majority of cases among females may be attributed to cultural variation in access to healthcare [1].

The study indicated that people resided in urban areas are more likely to wound infection than those rural areas. This study is slightly similar to the study demonstrated in Ethiopia, by Molla and his collegue in 2023, in which DFI in the urban was 62.4% while the rural rates was 37.6% [29]. Moreover, another study conducted in Erbil city-Iraq, who showed that the infection in the urban was 54% and the rural was 46%. This may be due to the target patients and type of the studies [11], moreover, there are several indicators that assess the general socioeconomic factors in a neighborhood, that provide a quick evaluation of the neighborhoods inhabited by residents [30].

On the other hand, obese individual had the highest rates of infection compared to overweight and normal weight and the PsA DWIs rates among normal followed by overweight and obese. This study was same to the study reported in Iraq, where they found that that participants with obesity had a high number of DFI [5]. Another study was conducted in USA, they found that the average BMI among DFI patients with PsA and without PsA were 33.6 and 30.6, respectively [31]. Obesity rates increased dramatically over the following decades, leading the World Health Organization to classify it as a global epidemic threatening public health. The organization stated that obesity/overweight is one of the most significant risks to human health [15]. Instance, atherosclerosis is one of the diseases caused by obesity, and enable one of the causes of DFUs [5].

Their educational level varied, illiterate was more likely to be infected by DM than other, and in all cases the prevalence of PsA DWIs were lesser or equal to the incidence of non-PsA DWIs. There is no study related the education to PsA DWIs. In corresponded with this study, a study performed in Colombia, they reported that most people with DM had not completed their primary education [32]. Furthermore, in Kirkuk city, Iraq a study reported that an illiterate rate of (24%), and higher education rate of (4%) [4]. On the other hand, most other studies showed conflicting results, indicating that the high rate was found among DFI with higher education [5], and high school and university [33], while the rate was lower among illiterate patients [29]. Another study stated that people with lower levels of education are more likely to develops DM and more likely to be affected by its complications [30]. These results indicated that the level of education is important in taking care of metabolic diseases and in the care of health protection [4]. Diabetic patients with low level of education are unable to control their blood sugar levels and unaware of information about poor wound hygiene.

Most of the DM patients were housewife and unemployed, and the rates of PsA DWIs were high compared to non-PsA DWIs. This study was partly in agreement to the studies performed in Iraq, their founding as follows: housewife (37.6%), employee (12.4%), daily labor (22.8%), and pension (27%) [5]. Moreover, a study in Ethiopia, a study included that housewife (19%), government employee (26.8%),

daily labor (5.3%), and pension (5%), unemployed (3.8%), and other (farmer and merchant) [29]. Our research found that the highest rates were among non-working and non-occupational patients of both sexes, who were uneducated, and had a higher rate of PsA DWIs.
The rates of non-smoker among patients were higher than former and current smokers among all non-PsA DWIs was higher than PsA DWIs. The result were same to the previous study, they found that non-smoker was with rate of (74.9%), followed by smoker (1.6%) and ex-smoker (9.5%) [5]. Another study confirmed that the foot infection by PsA was higher than in those without PsA, at 37% and 19%, respectively [8]. This study found that no correlation between PsA DWIs and smoking, being non-smoker having higher rates than current and former smoker.

The result of the present and absent of family history of diabetics is disagreement to the study conducted in Iraq, they confirmed that the rates of cases of family history of diabetics among DFI was as follows: negative (47%), positive (36%), and unknown (16.2%) [5]. Furthermore, a study found that the rate of negative cases was 62.2% and positive cases was 37.8% [29]. Another study also reported that rate negative cases were 72.7%, while the rate of positive cases was 27.3%. The study revealed that DM patients with a family history of diabetics are more likely to develop DM than those without a family history [34]. A study in China stated that, patient may have diabetes due to the effects of genetic factors from a family history of diabetes [35].

The mean and standard deviation (SD) were similar to those of the various studies conducted on the mean of HbA1c level among DFU, such as: 8.3 ± 2.2 [24], 8.94 ± 2.1 [36], and 8.61 ± 2.24 [37]. however, another study indicated a higher average HbA1c level, reaching 10.26 ± 2.0 [5]. On another hand, a study conducted in USA, on 257 patients with HbA1c values showed that the overall mean HbA1c value was 8.3% [8]. The HbA1c value was higher in patients with PsA DFI compared to non-PsA DFI groups (8.5 vs 7.3). It is known that chronic hyperglycemia in DM patients disrupts wound healing [38], and that wound size increases with elevated HbA1c level. It is also associated with other complications and increased mortality rate. Therefore, maintaining the low level of HbA1c in DM patients is essential for preventing diabetic foot and other complications [39].

Various study had been conducted on the mean ± SD of the duration of DM by year, such as: 14.5 ± 4.5 [24], (11.1%) [37], 13.6 ± 8.5 [36], ≤10 equal to (40.3%) [33], while >10 was equal to (59.7%), and 13.54 ± 6.98 [5]. Otherwise, the mean duration of ulcer by months among DFU as follows: 14.5 ± 4.5 [24], and 1.8 [37].

The present study was taken samples from various anatomical parts of the body, which differs from all other studies that focused only on foot infection. Thus, the study conducted on DFI anatomical part included the toes, comprising (44%), of which (30%) were PsA DFI and (45%) were non-PsA DFI; the lateral under foot was (29%), with (22%) being PsA and (30%) being non-PsA DFI; the heel was (16%), with (19%) being PsA DFI and (16%) being non-PsA DFI; other part was (14%), with (37%) being PsA DFI and (11%) being non-PsA DFI; (9%) had no listed part, (4%) had PsA DFI and (9%) were non-PsA DFI [8]. There was correlation between wound infection according to other anatomical parts of the body with DM ($p = 0.001$), with the exception of the Foot.

Based on the degree of wound infection the PsA DWIs was with high rates only among ulcer with cellular death and gangrene while the lesion severity for the other is low compared to non-PsA DWIs. The study found that the types of DFI included: ulcers which were (70), with (82%) PsA DF and (68%) non-PsA DF; abscess which were (21%), with (11%) PsA DF and (22%) non-PsA DF; and ulcer and abscess together, which were (10%), with (7%) PsA DF and (10%) non-PsA DF [8]. Furthermore, out of 200 DFI samples, (44%) were ulcer, (20%) were cellulitis, (32%) were abscesses, (24%) were gangrenous, and (4%) were neuropathic ulcer [7]. Moreover, the foot with ulcer were (50.2%) [37]. The most common complication in DM patients was wound infection in the different anatomical body, which was caused by ulceration, inflammation, and lower limb amputation due to gangrene, often leading to widespread morbidity, frequent hospitalization, and death [7].

Of 112/141 (90.76%) DM patients with wound infection, they had other comorbidity and complication, various studies have been conducted on DFU and its relationship to patients complication such as: among 54 DFU infection, 11 had hypertension, 8 had retinopathy, 11 had nephropathy, 24 had neuropathy, 10 had osteomyelitis [24]. Meanwhile in 2023, a study indicated that (62.2%) of patients had other chronic disease and comorbidities, and (37.8%) had no comorbidities. Among the comorbidities, (30.8%) had hypertension, (6.5%) had hypertension and heart disease, (6.3%) had heart disease and kidney disease, (5.3%) had heart disease, (5.3%) had kidney impairment, (3.3%) had arthritis, and (2.0%) hah hypertension, heart and kidney disease, (1.0%) hah osteoarthritis, (1.8%) had other disease [29]. Furthermore, another study showed that (43.2%) of the cases had hypertension, (10.7%) had hypercholesterolaemia, and (6.8%) had others comorbidities. complications were present in (16%) cases, including (1.9%) nephropathy, (5.8%) retinopathy, (4.9%) neuropathy, and (3.4%) others condition, while (84%) did not suffer from any complication [33]. Ultimately, inadequate blood supply to body parts, localized infection, and neuropathy, can eventually lead to complete loss of blood vessels, necrosis of dead tissue and leading to osteomyelitis, resulting in gangrene and amputation [7], [39]. If DM patients with wound infection have more than three complications, this is an indicator of amputation. Therefore, the government should confirm the high promote education and taking care for DM through timely detection and complications treatment, in order to minimize major amputations [26].

Regarding their treatment methods, the highest rate of the types of treatment among PsA DWIs was insulin and oral hypoglycemic tablets, followed by diets and oral hypoglycemic tablets, and insulin alone. On the other hand, (90.07%) of the DM did not exercise and (9.93%) exercised. Another study stated that among DM, (26.6%) received oral hypoglycemic agents, (43.6%) received insulin, (29.4%) received both insulin and oral hypoglycemic agents, and (0.3%) received other treatments, while approximately (3.3%) had no treatment [37]. Furthermore, additional study indicated that patients taken various types of treatment, such as: diet alone (10.7%), oral hypoglycemic tablets (55.8%), insulin alone (8.3%), and both oral hypoglycemic tablets and insulin (12.6%), while (12.6%) cases no treatment given. Furthermore, oral hypoglycemic agent (42.9%), insulin (31.6%), and oral hypoglycemic agents and insulin (25.6%) [29]. Approximately (43.2%) of DM patients exercise, while (56.8%) did not exercise [33].

The risk of developing type 2 diabetics can be reduced by following a healthy diet and exercising regularly. Furthermore, glucose manufacture in the liver can be reduced by enhancing insulin action, which lowers the level of HbA1c, and this in turn relieve the risk of developing DM complications [40].

Most of the DM (91-93%) with wound infection in this study was dressing there wound by using antiseptic, and the rate of PsA DWIs was low compared to non-PsA DWIs. Most patients are treated by dressing and debridement the wounds [7], and two-thirds of the DM patients with wound infection using an antimicrobial dressing [37].

However, regarding wound dressings and their selection, the focus must be on the best practice recommendations (BPRs) and clinical practice guidelines (CPGs) that specify the best way for dressing and patient cares methods, the best moisture balance, and assistance in surgical cleansing for wound care. Wounds may heal more quickly when treated with an antimicrobial dressing compared to those that do not use an antimicrobial dressing [6].

This study has several limitations. The cross-sectional design prevents establishing causal or temporal relationships between risk factors and *Pseudomonas aeruginosa* infection. Additionally, the study was conducted in a single province with a relatively small sample size, potentially restricting the strength and applicability of the findings. Nevertheless, the study has notable strengths. Clinical wound samples were collected over a full 12-month period, reducing seasonal bias. Samples were obtained from multiple hospitals and private clinics across Zakho and Duhok, enhancing representativeness. *Pseudomonas aeruginosa* identification was performed using conventional biochemical methods and confirmed by 16S rDNA PCR, ensuring high diagnostic accuracy. The study also assessed a wide range of demographic, clinical, and behavioral variables, including age, BMI, smoking status, wound characteristics, and glycemic control, providing a comprehensive evaluation of potential risk factors. Finally, antimicrobial susceptibility testing was conducted using CLSI-standard disc diffusion methods, ensuring reliable and comparable resistance profiles.

Conclusion

Diabetic mellitus patients with wound infection are complex cases, in many cases it led to a major complication. The presence of *P. aeruginosa* among DM with wound infection is strongly associated with BMI and a location of wound infection, with leg and foot wounds being more frequently positive. However, statistically no significant associations were found between infection status and age group, sexes, residence, HbA1c level, family history of diabetes, educational, occupational status, smoking, type or degree of wound, comorbid conditions, type of diabetic treatment, wound dressing or use of antiseptics, and exercise status.

Therefore, it is essential to detect and treat the wound in its early stages, rather than in advanced stages when the infection has spread to deep tissues. Parents with a family history of DM should be concerned about their offspring being diagnosed at a younger age and also if they have a high BMI. To improving the knowledge, the most necessary thing is the educational level of the health professional,

especially the nurse, which is crucial in helping the patient with DM on how to take care, particularly regarding wound infections and preventing complications. Furthermore, physicians should conduct a clinical trial to select antimicrobial and their use during dressings, in order to choose what is best for their patients.

Acknowledgments
We would like to express sincere gratitude to all participants who took part in this study.

Conflict of Interest
The authors declared no conflict of interest regarding study content.

Financial Disclosure
The study did not receive any financial support

References

[1] Ghobadi A, Sarbarzeh PA, Jalilian M, Abdi A, Manouchehri S. Evaluation of Factors Affecting the Severity of Diabetic Foot Ulcer in Patients with Diabetes Referred to a Diabetes Centre in Kermanshah. Dovepress. 2020; 13: 693–703. http://doi.org/10.2147/DMSO.S242431

[2] Sivanmaliappan TS, Sevanan M. Antimicrobial Susceptibility Patterns of *Pseudomonas aeruginosa* from Diabetes Patients with Foot Ulcers. International Journal ofMicrobiology. 2011; 2011: 1–4. doi: 10.1155/2011/605195.

[3] Zhang J, Deng Y, Wan Y, Wang J, Xu J. Diabetes duration and types of diabetes treatment in data-driven clusters of patients with diabetes, Frontiers in Endocrinol. 2022; 10 (3389): 1–12. doi: 10.3389/fendo.2022.994836.

[4] Amen MR, Kumait AS, Jaafr AY. Risk Factors for Diabetic Foot Ulceration Among Patients: A Cross-Sectional Study in Kirkuk City , Iraq. Journal of Pioneering Medical Sciences.2025; 14 (07): 112–116, 2025.

[5] Mohammed AG, Al-waeli DK, Odhaib SA, Altemimi MT. Assessing the Features of Diabetic Foot Ulcers among Individuals with Type 2 Diabetes Mellitus in Thi Qar, Iraq. F1000 Research. 2025; 13 (539): 1–14.

[6] Ivory JD, Gethin G, Mcintosh C. Antimicrobial dressings for treating local infection in patients with diabetic foot ulcers. The Diabetic Foot Journal. 2018; 21 (3): 155–159.

[7] Kothari B. Clinical study and surgical management of diabetic foot: An observational study. International Journal of Surgery Science. 2022; 6 (1): 130–133.

[8] Veve MP, Mercuro NJ, Sangiovanni RJ, Santarossa M, Patel N. Prevalence and Predictors of *Pseudomonas aeruginosa* Among Hospitalized Patients With Diabetic Foot Infections. Open Forum Infectious Diseases. 2022; 9 (7): 1–8.

[9] Al-Khudhairy MK, Al-Shammari MMM. Prevalence of metallo-β-lactamase–producing *Pseudomonas aeruginosa* isolated from diabetic foot infections in Iraq. New Microbe and New Infect. 2000; 35 (100661): 1–6.

[10] Al Abbas LA, Khaddam W, Shreibati F. INVESTIGATING BACTERIA ISOLATED FROM DIABETIC FOOT ULCERS AND STUDYING THEIR SENSITIVITY TO ANTIBIOTICS –SYRIA. Bulletin of Pharmaceutical Sciences Assiut University. 2022; 45 (1): 451–458.

[11] Khader KA, Raad AZ. Molecular detection of toxA and lasB virulence genes of *Pseudomonas aeruginosa* strains isolated from Diabetic Foot Ulcers in Erbil, Iraq. Journal of Applied and Natural Science. 2025; 17 (3): 1386–1392.

[12] Muhammed S, Aziz AD, Ahmed MJ. Microbial Identification And Antibiotic Resistance Patterns In Diabetic Foot Ulcers From Patients In Erbil, Kurdistan Region, Iraq. The Review of DIABETIC STUDIES. 2025; 21: 634–644.

[13] Qadir AN, Mahmoud BM, Mahwi TO, Mohammed D, Arif R, Mahmood SO. Prevalence of Microorganisms and Antibiotic Sensitivity Among Patients with Diabetic Foot Ulcer in Sulaimani City, Iraq. Hospital Practices and Research. 2020; 5 (2): 56–63. doi: 10.34172/hpr.2020.11.

[14] Horcajada JP, Montero M, Oliver A, Sorlí L, Luque S, Gómez-Zorrilla S, et al. Epidemiology and Treatment of Multidrug-Resistant and Extensively Drug-Resistant. Clinical Microbiology Reviews. 2019; 32 (4): 1–52.

[15] Kolimechkov S, Petrov L. The Body Mass Index : A Systematic Review. Journal of Exercise Physiology and Health. 2020; 3 (2): 21–27.

[16] Apridamayanti P, Meilinasary KA, Sari R. Antibiotic Sensitivity in *Pseudomonas aeruginosa* of Diabetic Patient ' s Foot Ulcer. Pharm Sci Res. 2016; 3 (2): 80–87.

[17] Callahan A, Liam P. A Clinical Review of Necrotic Skin Lesions as Presentations of Systemic Infection. Preprints.org. 2025: 0–17. doi: 10.20944/preprints202507.1668.v1.

[18] Alexander SK, Strete D. Microbiology: A Photographic Atlas For The Laboratory. USA: Daryl F, 2001.

[19] Cheesbrough M. District Laboratory Practice in Tropical Countries. Second edition. New York, USA: Cambridge University Press, 2006.

[20] Spilker T, Coenye T, Vandamme P, Lipuma JJ. PCR-Based Assay for Differentiation of *Pseudomonas aeruginosa* from Other Pseudomonas Species Recovered from Cystic Fibrosis Patients. JOURNAL OF CLINICAL MICROBIOLOGY. 2004; 42 (5): 2074–2079. doi: 10.1128/JCM.42.5.2074.

[21] Polse RF. Bacteriological and Molecular Identification of *Pseudomonas aeruginosa* fron Burns and Wound Infection in Duhok Province/Kurdistan Region/Iraq. Doctoral dissertation, University of Zakho, 2024.

[22] Ausubel FM, Brent R, Kingston RE, Moore DD, Seidman JG, Smith JA, et al. Current Protocols in Molecular Biology. Ringbou. Wiley S, 2003.

[23] Polse RF, Khalid HM, Mero WMS. Distribution of blaOXA-10, blaPER-1, and blaSHV genes in ESBL-producing *Pseudomonas aeruginosa* strains isolated from burn patients. Scientific Reports. 2023; 13 (18402): 1–8. doi: 10.1038/s41598-023-45417-4.

[24] Samani S, Parsa H. Microbiological Features and Risk Factors in Patients With Diabetic Foot

Ulcers. Index Wounds. 2015; 27 (11): 308–312.

[25] Chatterjee D, Sivashanmugam K. Insights on MDR Mechanism of *Pseudomonas aeruginosa* with Emphasis on Diabetic Foot Ulcer in the Indian Subcontinent. Journal of Pure and Applied Microbiolgy. 2024; 18 (2): 837–852. doi: 10.22207/JPAM.18.2.08.

[26] Yao Y, Chen L, Qian Y. Age Characteristics of Patients With Type 2 Diabetic Foot Ulcers and Predictive Risk Factors for Lower Limb Amputation: A Population-Based Retrospective Study. Journal of Diabetes Research. 2024; 2024 (1): 1–8. doi: 10.1155/jdr/2380337.

[27] Garousi M, Monazamitabar S, Mirazi H, Farrokhi Z, Khaledi A, ShakerimoghaddamA. Epidemiology of *Pseudomonas aeruginosa* in diabetic foot infections: a global systematic review and meta-analysi. Germs. 2-23; 13 (4): 362–372.

[28] Mahmoud HM, Abdul S, Abid A, Qadir D. Prevalence of bacterial types in Wagner grade three of diabetic foot ulcer. Iraqi Journal of Science. 2014; 55 (3): 1232–1235.

[29] Molla IB, Berhie MA, Debele KA, Germossa GN, Hailu FB. Persons with Diabetes' Perceptions of Family Burden and Associated Factors. Journal of Diabetes Research. 2023; 2023: 1–8.

[30] Schmidt BM, Huang Y, Banerjee M, Hayek SS, Pop-busui R. Residential Address Amplifies Health Disparities and Risk of Infection in Individuals With Diabetic Foot Ulcers Residential Address Amplifies Health Disparities and Risk of Infection in Individuals With Diabetic Foot Ulcers. Diabetes Care. 2024; 47: 508–515.

[31] J. Lau, J. Huang, and Z. K. Escobar, "Antipseudomonal Antibiotics in Diabetic Foot Infections : A Practical Perspective From a Community Hospital," Open Forum Infect. Dis., vol. 11, no. 6, pp. 1–4, 2024, doi: 10.1093/ofid/ofae258.

[32] Ramirez-Perdomo C, Perdomo-Romero A, Rodríguez-Vélez M. Knowledge and practices for the prevention of the diabetic foot. Rev Gaúcha Enferm. 2019; 40 (e20180161): 1–7.

[33] Ghanem SS, Abdulkreem EM, Alfurayh MS, Ahmed AA, Rikabi HA. The Role of HbA1c in the Follow-Up and Control of Diabetes Mellitus. Cureus. 2025; 17 (2): 1–7. doi: 10.7759/cureus.78357.

[34] Erasmus RT, Blanco EB, Okesina AB, Arana JM, Gqweta Z, Matsha T. Importance of family history in type 2 black South African diabetic patients. Postgraduate Medical Journal. 2001;77:323–325. doi: 10.1136/pmj.77.907.323.

[35] Peng H, Wang M, Guo H, Hou T, Li Y, Zhang H, etal. Parental Transmission of Type 2 Diabetes Risk in Offspring: A Prospective Family-Based Cohort Study in Northern China. Nutrients. 2025; 17 (1361): 1–12.

[36] Alharithy MK, Alobaylan MM, Alsugai ZO, Alswat KA. IMPACT OF FAMILY HISTORY OF DIABETES ON DIABETES CONTROL AND COMPLICATIONS. ENDOCRINE PRACTICE. 2018; 24 (9): 773–779. doi: 10.4158/EP-2018-0071.

[37] Ndosi M, Wright-Hughes A, Brown S, Backhouse M, Lipsky BA, Bhogal M, et al. Prognosis of the infected diabetic foot ulcer: a 12-month prospective observational study. Diabetic Medicine. 2018; 35: 78–88. doi: 10.1111/dme.13537.

[38] Fesseha BK, Abularrage CJ, Hines KF, Sherman R, Frost P, Langan S, et al. Association of Hemoglobin A 1c and Wound Healing in Diabetic Foot Ulcers. Diabetes Care. 2018; 41: 1478–1485. doi: 10.2337/dc17-1683.

[39] Dogan M, Onar LC, Aydin B, Gumustas SA. Is high level of hemoglobin A1C an indicator for extended period of antibiotherapy in diabetic foot ulcers? Infectious Diseases & Microbiology. 2019; 6 (1): 21–27. doi: 10.14744/nci.2018.25582.

[40] Amer BE, Abdelgalil MS, Hamad AA, Abdelsayed K, Elaraby A, Abozaid AM, et al. Metformin plus lifestyle interventions versus lifestyle interventions alone for the delay or prevention of type 2 diabetes in individuals with prediabetes : a meta-analysis of randomized controlled trials. Diabetology & Metabolic Syndrome. 2024; 16 (273): 1–14.

Click here to access/download;Manuscript;Clean-Manusceipt-Pseudomonas research 25-04-2026.docx

Prevalence and associated risk factors of *Pseudomonas aeruginosa* infection in Diabetic wounds in Duhok province, Iraq

Marwa S. Ibrahim[1] and Ibrahim A. Naqid[2*]

[1]Department of Medical Laboratory Technology, College of Health and Medical Techniques-Shekhan, Duhok Polytechnic University, Duhok, Kurdistan Region, Iraq

Department of Biomedical Sciences, College of Medicine, University of Zakho, Kurdistan Region, Iraq

Abstract

Background and aims

Diabetes mellitus is a chronic metabolic disorder and a major global health problem, predisposing patients to wound infections, with *Pseudomonas aeruginosa* being a frequent and resistant pathogen. This study aimed to identify *P. aeruginosa*, assess associated risk factors, and determine its antibiotic susceptibility pattern in diabetic wound infections in Duhok Province, Kurdistan Region, Iraq.

Methods

This cross-sectional study was conducted from June 2024 to July 2025 in Duhok Province, Kurdistan Region, Iraq. A total of 141 wound swab samples were collected from diabetic patients aged ≥18 years. *P. aeruginosa* was identified using biochemical tests, Vitek®2, and 16S rDNA PCR. Antimicrobial susceptibility testing was performed by the disc diffusion method on Mueller–Hinton agar, and data were analyzed using GraphPad Prism software.

Results

Out of 141 wound infection samples collected from diabetic patients, *P. aeruginosa* was isolated in 44.68% of cases. Overall, sociodemographic factors showed no significant association with PsA infection, except for occupational status in the multivariable analysis 0.12 [0.02-0.97, p=0.046] and overweight 0.29 [0.10-0.82, p=0.019]. Clinically, most variables including HbA1c, duration of diabetes, comorbidities, treatment type, and wound characteristics were not significantly associated with PsA infection. The only significant predictor was wound location, with leg-region infections showing a higher risk of PsA 3.94 [1.24-12.5, p=0.02]. *P. aeruginosa* isolates showed high levels of multidrug resistance, especially to aztreonam, ceftazidime, cefepime, and tobramycin. Colistin showed the lowest resistance and highest sensitivity, followed by relatively better susceptibility to carbapenems.

Conclusion

P. aeruginosa is a common cause of wound infections in patients with diabetes mellitus, with its prevalence significantly associated with body mass index, occupational status and wound location, with leg-region wounds showing a higher risk. *P. aeruginosa* isolates demonstrated high multidrug resistance, although colistin and carbapenems retained relatively better activity. Early detection and appropriate antibiotic selection are essential to reduce complications and improve patient outcomes.

Keywords:
Pseudomonas aeruginosa; Diabetic wound infection; Prevalence; Risk factors; Antibiotic susceptibility; Duhok Province; Iraq

Introduction

Diabetes mellitus (DM) is a chronic metabolic disorder characterized by persistent hyperglycemia and disturbances in carbohydrate and lipid metabolism, resulting in insulin resistance [1]. It is recognized as a major global public health problem and an emerging epidemic, contributing significantly to morbidity and disability in both developing and developed countries [1, 2]. DM is classified into type 1 and type 2 diabetes [3]. Patients with type 2 diabetes are at increased risk of developing multiple complications, including hypertension, cardiovascular disease, renal failure, cerebrovascular accidents, retinopathy, and neuropathy [4,5]. In addition to comorbidities, several factors influence the risk and severity of infection. These include local factors such as poor tissue perfusion, presence of foreign bodies, necrosis, wound location and size, and poorly controlled diabetes. Behavioral factors such as malnutrition, non-adherence to treatment plans, and smoking, as well as social determinants such as educational level and socioeconomic status, also play important roles [6].

Furthermore, peripheral ischemic disease is a frequent and severe complication that can lead to ulceration and wound infection, particularly diabetic foot ulcers (DFU) [1]. Ulceration is the most common clinical manifestation of wound infections, followed by cellulitis and gangrene. The dorsum of the foot is the most commonly affected site, followed by the forefoot and toes [7]. This condition may further progress to osteomyelitis [8]. Among DM patients who develop foot ulcers, approximately 20–30% require minor amputation, while about 5% undergo major amputation [6].

Chronic wound infections are typically polymicrobial, and microbial colonization of a localized wound can lead to extensive replication on the wound surface. As the microbial load increases, pathogens may penetrate deeper tissues, resulting in clinical signs and symptoms in diabetic patients [6]. Both Gram-positive and Gram-negative bacteria can cause wound infections, with *P. aeruginosa* being one of the most common pathogens in diabetic patients [9, 10]. This organism can cause severe infections in multiple sites, including pneumonia, urinary tract infections, keratitis, and soft tissue infections of open wounds, particularly diabetic foot ulcers and burn injuries [11]. It produces multiple toxins, evades phagocytosis, and exhibits broad antibiotic resistance, making it a highly virulent and difficult-to-treat pathogen [2]. A previous study in Erbil, Iraq reported a prevalence of *P. aeruginosa* in diabetic foot infections of 35.33% [11]. Another study conducted in Erbil [12] found *P. aeruginosa* to be the most common Gram-negative bacterium, accounting for 21.43% of isolates. In contrast, a study in Sulaymaniyah reported a lower prevalence of *P. aeruginosa* among diabetic foot infections (8.5%) [13].

Antibiotic resistance in *P. aeruginosa* has become a major global public health concern, particularly due to the limited availability of effective alternative treatment. Infections caused by this pathogen significantly contribute to morbidity and mortality worldwide. According to the European Centre for Disease Prevention and Control, 13.7% of *P. aeruginosa* isolates are resistant to at least three

antimicrobial groups, while 5.5% are resistant to all five groups under surveillance. This highlights the growing challenge of multidrug resistance and the need for effective antibiotic stewardship and infection control measures [14]. The declining sensitivity of *P. aeruginosa* to commonly used antibiotics underscores the urgent need to prevent antibiotic overuse and to strengthen antimicrobial stewardship efforts, which may help curb resistance in the future [10]. Furthermore, antibiotic selection for the treating aeruginosa infections is often inconsistent, highlighting the critical importance of choosing appropriate, evidence-based therapeutic regimens [11].

Most previous studies in the Kurdistan Region of Iraq have focused mainly on *P. aeruginosa* in diabetic foot ulcers. Therefore, the present study aimed to isolate P. aeruginosa from wound infections at various anatomical sites among diabetic patients in different hospitals in Duhok Province, Kurdistan Region, Iraq. In addition, it aimed to identify key risk factors associated with *P. aeruginosa* wound infections and determine the antimicrobial susceptibility patterns of the isolates.

Material and method

Study design, and sample collection

This cross-sectional study was conducted in Duhok Province, Kurdistan Region, Iraq, from July 2024 to July 2025. A total of 141 wound samples were collected from diabetic outpatients (77 females, 64 males) aged ≥18 years at Zakho General Hospital and Azadi Teaching Hospital using a **convenience sampling** approach, based on the availability of eligible patients during the study period. The sample size of 141 was based on all eligible diabetic wound infection cases collected during the study period in selected hospitals; therefore, a census sampling approach was applied. Wounds were first cleaned with normal saline, after which samples were obtained using sterile swabs and transported in Amies medium. All samples were cultured on appropriate media at the Microbiology Laboratory of Zakho Technical Institute. Written informed consent was obtained from all participants before sampling.

Severity of wound infection:

The severity of wound infection was classified based on the degree of tissue involvement. The severity was defined based on the degree of tissue involvement and clinical presentation, including ulcers (necrosis with erythema, pain, and discharge), abscess formation (pus collection), cellular necrosis (tissue death due to hypoxia or vascular occlusion), and gangrene (advanced necrosis with or without systemic symptoms). Poor skin care and unrecognized skin injuries may progress to chronic ulcers, facilitating microbial invasion of skin tissue. As infection extends to deeper layers, it may involve muscles, joints, and tendon sheaths, leading to pus accumulation, tissue necrosis, and abscess formation. Diabetic ulcers are open skin wounds characterized by necrosis, surrounding erythema, pain, and purulent discharge [16]. Necrosis may also result from vascular occlusion or trauma, leading to tissue hypoxia and cell death. Advanced necrosis can results in eschar formation and may progress to wet or dry gangrene, often accompanied by systemic symptoms such as fever and malaise. Wet gangrene is frequently associated with foul odor due to secondary infection [17

Body Mass Index (BMI)

Patients' weight and height were measured, and body mass index (BMI, kg/m²) was calculated and classified as underweight (<18.5), normal weight (18.5–24.9), overweight (25.0–29.9), or obese (≥30.0).[15].

Bacterial Isolation and Identification

Wound swab samples were immediately cultured on Blood, MacConkey, and Chocolate agars and incubated overnight at 37 °C. Suspected *P. aeruginosa* colonies were selected and sub-cultured on MacConkey agar to obtain pure isolates. Further characterization was performed using biochemical tests, including peptone water, methyl red, Voges–Proskauer, Simmons citrate, Triple Sugar Iron (TSI), cetrimide agar, oxidase test, and Gram staining [18]. All isolates were additionally confirmed using the automated Vitek®2 system (bioMérieux, France).

Antibiotic susceptibility test

Antimicrobial susceptibility *test* of *P. aeruginosa* isolates was performed using twelve antibiotic discs (Bioanalyses, Turkey). The disc diffusion method was performed on Mueller-Hinton agar to assess antibiotic susceptibility and resistance. After 24 hours of incubation, the diameters of the inhibition zone around each disc were measured, and compared to standard reference value. Based on the size of the inhibition zones, the isolates were interpreted as susceptible or resistant in accordance with Clinical and Laboratory Standards Institute (CLSI) guidelines (M100 35rd Edition 2025) [19].

Molecular investigation

Genomic DNA extraction

Genomic DNA of all isolated *P. aeruginosa strains* was extracted, using a commercial extraction kit (PrimePrepTM Genomic DNA Extraction Kit, GeNet Bio, Korea). Following extraction, the concentration and purity of the DNA samples were determined using a NanoDrop spectrophotometer (Thermo scientific, USA). The extracted genomic DNA was subsequently used as a template for polymerase chain reaction (PCR) amplification. All procedures were conducted at the Research Center of the Biology Department, Faculty of Science, University of Zakho.

Primers

A 16S rDNA species-specific primer set was used for the molecular identification of the 16S rDNA gene in *P. aeruginosa.* The primer sequences used were: forward (GGGGGATCTTCGGACCTCA) and reverse (TCCTTAGAGTGCCCACCCG), yielding an expected amplicon size of 956 base pairs as previously described [20]. A positive control for the 16S rDNA gene was obtained from [21] with the accession No. (OR882879).

PCR amplification and gel electrophoresis

The final volume of the PCR amplification reaction was 20 µl consisting of 8 µL of master mix (2x Taq Mix, manufacturer by GDSBio- China, Lot number: 130) 2 µL of forward primer**,** 2 µL of reverse primer**,** 2 µL of genomic DNA (25–50 ng/µL)**,** and 6 µL of PCR-grad. Following preparation of the reaction mixture, the tubes were placed into the thermal cycler for amplification. The amplification conditions are summarized in Table (1). After amplification, the PCR products were analysed by gel electrophoresis. A 1.25% (w/v) agarose gel was prepared using 1× Tris–Borate–EDTA (TBE) buffer**,** and samples were electrophoresed for 5 minutes at 45 V, followed by 30 minutes at 80 V. DNA bands were subsequently visualized under a UV transilluminator (Cleaver Scientific, UK). The procedure followed the standard protocols described by by Ausubel et al [22].

Table 1: The amplification condition of 16S rDNA PCR analysis for 16S rDNA primer

Initial denaturation	Denaturation	Annealing	Extension	Final extension	Reference
94 °C	92 °C	54 °C	72 °C	72 °C	[20]
2 min	20 sec	20 sec	40 sec.	5 min	
1 cycle	25 cycles			1 cycle	

Inclusion and exclusion criteria

All clinical samples obtained from diabetic patients aged ≥ 18 years who met the diagnostic criteria for wound infection and attended to hospitals or outpatients' clinics were included in the study. The exclusion criteria were as follows: (1) patients younger than 18-years of age, (2) patients who declined participation in the study and (3) patient who had received antimicrobial therapy prior to sample collection.

Ethics

Ethical approval for this study was obtained from Medical and General Health Research Ethics Committee from the Ministry of Health, Duhok Directorate General of Health, under the reference number (29052024-4-2).

Statistical analysis

raphPad Prism 10.6.1 was used for statistical analysis. Binary logistic regression was employed to calculate odds ratios (ORs), 95% confidence intervals (95% CIs), and p-values. All variables were included in a multivariate logistic regression model. A p-value < 0.05 was considered statistically significant, indicating strong evidence of a relationship between the independent variables and the outcomes under investigation.

Result

Demographic characteristics of the diabetic patients with wound infection

A total of 141 patients were included in the analysis. *Pseudomonas aeruginosa*–associated diabetic wound infections (PsA DWIs) were identified in 63 patients (44.68%), whereas non-PsA DWIs were detected in 78 patients (55.32%). The sociodemographic characteristics of patients with diabetic wound infections, with and without *P. aeruginosa*, are presented in Table 2.

The age distribution among patients with diabetes mellitus (DM) showed no statistically significant association with PsA infection. The highest proportion of cases was observed in the 50–59-year age group (31.91%), with a higher prevalence of non-PsA DWIs than PsA DWIs. The odds ratios (ORs) for PsA infection were 1.13 (95% CI: 0.44–2.91, p = 0.80), 1.17 (95% CI: 0.45–3.05, p = 0.75), and 1.31 (95% CI: 0.45–3.84, p = 0.63) for the 50–59, 60–69, and ≥70-year age groups, respectively, compared with the reference group (18–49 years). The mean age was also not significantly associated with PsA infection (OR = 1.00, 95% CI: 0.97–1.04, p = 0.78).

Similarly, sex distribution did not differ significantly between the two groups. Males had an OR of 0.74 (95% CI: 0.38–1.45, p = 0.38) compared with females. Females comprised 54.61% of the total sample, with comparable proportions in the non-PsA (51.95%) and PsA DWI (48.05%) groups.

Most participants resided in urban areas (78.01%), compared with 21.99% in rural areas. In both settings, the prevalence of PsA DWIs was lower than that of non-PsA DWIs. However, the difference was not statistically significant, with an OR of 1.21 for rural residence (95% CI: 0.55–2.69, p = 0.64).

Among all patients with diabetes mellitus (DM), obesity was the most prevalent BMI category (51.06%), with an equal distribution of PsA and non-PsA diabetic wound infections (DWIs). This was followed by the overweight group (31.91%) and the normal-weight group (17.02%). The highest proportion of PsA DWIs was observed in the normal-weight group (58.33%). Overweight individuals had significantly lower odds of PsA infection compared with the normal-weight group (OR = 0.29, 95% CI: 0.10–0.82, p = 0.019), reflecting a lower proportion of PsA DWIs (28.89%) relative to non-PsA DWIs (71.11%). In contrast, obesity was not significantly associated with PsA infection (OR = 0.71, 95% CI: 0.28–1.82, p = 0.48). Furthermore, the overall mean BMI was not significantly associated with PsA infection (OR = 1.03, 95% CI: 0.98–1.096, p = 0.26

Educational attainment varied among participants: 59.57% were illiterate, 21.48% had primary education, 13.48% had secondary or tertiary education, and 5.67% had higher education. Across all categories, the prevalence of PsA diabetic wound infections (DWIs) was less than or equal to that of non-PsA DWIs. Compared with the illiterate group (reference), the odds ratios (ORs) for PsA infection were 0.43 (95% CI: 0.18–1.04, p = 0.06) for primary education, 0.73 (95% CI: 0.27–1.99, p = 0.54) for

secondary/tertiary education, and 1.00 (95% CI: 0.24–4.27, p = 1.00) for higher education; none of these associations were statistically significant.

Approximately three-quarters (73.76%) of patients were not formally employed, including housewives (51.77%) and unemployed males (21.99%). The remaining participants were government employees (10.64%), daily laborers (9.93%), and pensioners (5.67%). A higher proportion of PsA DWIs was observed only among unemployed individuals (51.61%). However, no statistically significant association was found between occupational status and PsA infection in the unadjusted analysis, with daily laborers serving as the reference group. The ORs were 0.25 (p = 0.098) for government employees, 0.14 (p = 0.10) for pensioners, 0.97 (p = 0.96) for housewives, and 1.07 (p = 0.92) for unemployed individuals.

Regarding smoking status, non-smokers constituted the largest proportion of patients (65.96%), followed by former smokers (17.02%) and current smokers (17.02%). Non-smokers had a slightly higher prevalence of non-PsA DWIs compared with PsA DWIs (52.69% vs. 47.31%). In contrast, PsA DWIs were less frequent among former and current smokers compared with non-PsA DWIs. Neither former smoking (OR = 0.56, p = 0.22) nor current smoking (OR = 0.94, p = 0.90) showed a statistically significant association with PsA infection when compared with non-smokers. The number of patients with and without a family history of diabetes mellitus was nearly equal (74 vs. 67). In both groups, the prevalence of PsA DWIs was slightly lower than that of non-PsA DWIs; however, this difference was not statistically significant (OR = 1.40, p = 0.32).

In the multivariable analysis, some associations changed. The previously observed significant association for overweight individuals was no longer statistically significant (p = 0.08). In contrast, occupational categories such as government employees (p = 0.046) and pensioners (p = 0.047) became statistically significant, indicating an independent association with reduced odds of PsA infection.

Table 2: Sociodemographic characteristics of diabetes wound patients with *P. aeruginosa* and without *P. aeruginosa* infection

**Characteristic**	**Total No. (%) 141 (100)**	**Non-PsA DWIs No. (%) 78 (55.32)**	**PsA DWIs No. (%) 63 (44.68)**	**Unadjusted OR [95% CI, p-value]**	**Adjusted OR [95% CI, p-value]**
**Age Group (Year)**					
18-49	29 (20.57)	17 (58.62)	12 (41.38)	Ref	Ref
50-59	45 (31.91)	25 (55.56)	20 (44.44)	1.13 [0.44-2.91, p=0.80]	1.25 [0.41-3.81, p=0.69]
60-69	42 (29.79)	23 (54.76)	19 (45.24)	1.17 [0.45-3.05, p=0.75]	1.07 [0.33-3.48, p=0.91]
≥70	25 (17.73)	13 (52)	12 (48)	1.31 [0.45-3.84, p=0.63]	1.08 [0.29-4.08, p=0.91]
Mean ± SD	57.48 ± 10.66	57.26 ± 10.52	57.76 ± 10.91	1.00 [0.97-1.04, p=0.78]	------------------------
**Sex**					
Female	77 (54.61)	40 (51.95)	37 (48.05)	Ref	Ref
Male	64 (45.39)	38 (59.38)	26 (40.63)	0.74 [0.38- 1.45, p=0.38]	1.02 [0.06-17.7, p=0.99]
**Region**					

Urban	110 (78.01)	62 (56.36)	48 (43.64)	Ref	Ref
Rural	31 (21.99)	16 (51.61)	15 (48.39)	1.21 [0.55-2.69, p=0.64]	1.5 [0.60-3.78, p=0.39]
**BMI (kg/m^2)**					
Normal	24 (17.02)	10 (41.67)	14 (58.33)	Ref	Ref
Overweight	45 (31.91)	32 (71.11)	13 (28.89)	0.29 [0.10-0.82, p=0.019*]	0.35 [0.11-1.13, p=0.08]
Obesity	72 (51.06)	36 (50)	36 (50)	0.71 [0.28-1.82, p=0.48]	0.69 [0.23-2.09, p=0.51]
Mean ± SD	30.28 ± 5.82	29.80 ± 4.99	30.88 ± 6.69	1.03 [0.98-1.096, p=0.26]	------------------------
**Educational Level**					
Non-educational (illiterate)	84 (59.57)	42 (50)	42 (50)	Ref	Ref
Primary school	30 (21.48)	21 (70)	9 (30)	0.43 [0.18-1.04, p=0.06]	0.42 [0.14-1.23, p=0.11]
Secondary and Tertiary school	19 (13.48)	11 (57.89)	8 (42.11)	0.73 [0.27-1.99, p=0.54]	0.95 [0.26-3.5, p=0.94]
High educational (Institute or college)	8 (5.67)	4 (50)	4 (50)	1 [0.24-4.27, p=1]	4.84 [0.48-48.9, p=0.18]
**Occupational status**					
Daily Labor	14 (9.93)	7 (50)	7 (50)	Ref	Ref
Government Employee	15 (10.64)	12 (80)	3 (20)	0.25 [0.05-1.29, p=0.098]	0.12 [0.02-0.97, p=0.046*]
Pension	8 (5.67)	7 (87.5)	1 (12.5)	0.14 [0.01-1.49, p=0.10]	0.05 [0.003-0.96, p=0.047*]
Housewife	73 (51.77)	37 (50.68)	36 (49.32)	0.97 [0.31-3.05, p=0.96]	0.86 [0.04-16.9, p=0.92]
Unemployed	31 (21.99)	15 (48.39)	16 (51.61)	1.07 [0.30-3.77, p=0.92]	1.18 [0.24-5.77, p=0.84]
**Smoking**					
Non smoker	93 (65.96)	49 (52.69)	44 (47.31)	Ref	Ref
Former smoker	24 (17.02)	16 (66.67)	8 (33.33)	0.56 [0.22-1.43, p=0.22]	0.81 [0.23-2.85, p=0.74]
Current smoker	24 (17.02)	13 (54.17)	11 (45.83)	0.94 [0.38-2.32, p=0.90]	1.94 [0.49-7.63, p=0.35]
**Family History of DM**					
No	67 (47.52)	40 (59.7)	27 (40.3)	Ref	Ref
Yes	74 (52.48)	38 (51.35)	36 (48.65)	1.40 [0.72-2.74, p=0.32]	1.45 [0.66-3.22, p=0.36]

Note: **PsA** = *Pseudomonas aeruginosa,* **DWIs** = Diabetes Wound Infections, **OR**= odd ratio, * = statistically significant difference

The clinical characteristics of diabetic patients with PsA DWIs and non-PsA DWIs

The clinical characteristics of PsA diabetic wound infections (DWIs) and non-PsA DWIs are presented in Table 3. Among the study population, 14 patients had glycated hemoglobin (HbA1c) levels between 4 and 6.9, while 127 patients had HbA1c levels ranging from 7 to 16. The overall mean HbA1c level was 9.23 ± 2.18.The mean HbA1c level was slightly higher in patients with non-PsA DWIs compared with those with PsA DWIs (9.25 ± 1.98 vs. 9.20 ± 2.43); however, this difference was not statistically significant (OR = 0.99, 95% CI: 0.85–1.15, p = 0.90).

The mean duration of diabetes was 13.86 ± 7.72 years in patients with PsA diabetic wound infections (DWIs) and 13.97 ± 8.99 years in those with non-PsA DWIs, with no statistically significant difference between the groups (OR = 0.99, p = 0.87). Similarly, the duration of wound infection (in months) did not differ significantly between the two groups (OR = 0.99, p = 0.16), although a higher mean duration was observed in non-PsA DWI cases compared with PsA DWI cases (13.13 ± 38.41 vs. 5.82 ± 11.34 months). In terms of wound location, infections in the leg region were the most common (78.72%),

comprising 49.55% of PsA DWIs and 50.45% of non-PsA DWIs. Leg infections were significantly associated with the outcome, with approximately 2.7-fold higher odds of PsA infection (OR = 2.70, 95% CI: 1.11–6.58, p = 0.03).

Regarding wound type, wet wounds were the most prevalent (60.99%) and showed higher, though not statistically significant, odds of PsA infection (OR = 1.78, 95% CI: 0.87–3.64, p = 0.12). This was followed by dry wounds (35.46%) and mixed wounds (3.55%), with ORs of 1.19 (95% CI: 0.18–7.77, p = 0.86). Among wet wounds, PsA and non-PsA DWIs were equally distributed (50% each). In contrast, PsA DWIs accounted for 39% of dry wounds and 40% of mixed wounds, whereas non-PsA DWIs represented 64% and 60% of these categories, respectively.

A higher prevalence of PsA DWIs was observed in ulcers with cellular necrosis and gangrene (54.55%) compared with non-PsA DWIs (45.45%). In other lesion types, PsA DWIs were equal to or less frequent than non-PsA DWIs (36.36% in abscesses, 33.33% in cellular necrosis, 50% in gangrene, 37.84% in ulcers, and 47.95% in ulcers with cellular necrosis). Although some categories showed higher odds ratios (ORs) than abscesses, none were statistically significant (all p > 0.05) and confidence intervals (CIs) were wide. Among 112 patients with comorbidities, hypertension was most common (56%). PsA DWIs were more frequent in patients with periodontal disease (51.61%) and hypertension (48.1%), but no significant associations were found (all p > 0.05; CIs included 1). Regarding treatment, 31.12% used diet plus oral hypoglycemic agents, while 23.4% used diet plus insulin. PsA DWIs were most common among patients receiving insulin plus oral agents (66.67%). However, treatment type was not significantly associated with PsA DWIs (all p > 0.05; CIs included 1). Most patients (90.07%) did not exercise. Non-PsA DWIs were more prevalent in both active and inactive groups, with no significant association (p = 0.48), although exercise showed a lower OR (0.66; 95% CI: 0.21–2.08). Wound dressing was used by 91.49% of patients. PsA DWIs were less frequent than non-PsA DWIs, with no statistically significant difference.

All independent variables were not statistically significant predictors of PsA in the multivariable logistic regression analysis, except for wound location (leg region), which was significant (p = 0.02).

Table 3: Clinical characteristics of diabetes wound patients with *P. aeruginosa* and without *P. aeruginosa* infection					
**Characteristic**	**Total No. (%) 141 (100)**	**Non-PsA DWIs No. (%) 78 (55.32)**	**PsA DWIs No. (%) 63 (44.68)**	**Unadjusted OR [95% CI, p-value]**	**Adjusted OR [95% CI, p-value]**
**HBA1C (Mean ±SD)**	9.23 ± 2.18	9.25 ± 1.98	9.20 ± 2.43	0.99 [0.85-1.15, p=0.90]	0.88 [0.73-1.07, p=0.19]
**Duration of DM by year (Mean ±SD)**	13.92 ± 8.42	13.97 ± 8.99	13.86 ± 7.72	0.99 [0.96-1.04, p=0.87]	0.98 [0.92-1.03, p=0.40]
**History of ulcer by months (Wound infection)**	9.86 ± 29.29	13.13 ± 38.41	5.82 ± 11.34	0.99 [0.97-1.01, p=0.16]	0.98 [0.95-1.01, p=0.23]
**Location of wound infection (skin ulcer)**					
Non-Leg region	30 (21.28)	22 (73.33)	8 (26.67)	Ref	Ref
Leg region (Leg and Foot)	111 (78.72)	56 (50.45)	55 (49.55)	2.7 [1.11-6.58, p=0.03[*]]	3.94 [1.24-12.5, p=0.02[*]]
**Type of Wound infection**					
Dry	50 (35.46)	32 (64)	18 (36)	Ref	Ref
Wet	86 (60.99)	43 (50)	43 (50)	1.78 [0.87-3.64, p=0.12]	2.13 [0.90-5.22, p=0.09]
Both	5 (3.55)	3 (60)	2 (40)	1.19 [0.18-7.77, p=0.86]	0.63 [0.07-5.68, p=0.68]
**Degree of Wound infection (Lesion Severity) (Clinical presentations)**					
Abscess	11 (7.8)	7 (63.64)	4 (36.36)	Ref	Ref
Cellular death	3 (2.13)	2 (66.67)	1 (33.33)	0.88 [0.06-12.97, p=0.92]	0.59 [0.03-13.3, p=0.75]
Gangrene	6 (4.26)	3 (50)	3 (50)	1.75 [0.23-13.2, p=0.59]	1.53 [0.14-17.1, p=0.71]
Ulcer	37 (26.24)	23 (62.16)	14 (37.84)	1.07 [0.26-4.31, p=0.93]	1.93 [0.32-11.73, p=0.48]
Ulcer with Cellular Death	73 (51.77)	38 (52.05)	35 (47.95)	1.61 [0.43-5.98, p=0.48]	2.02 [0.36-11.52, p=0.43]
Ulcer with Cellular death and Gangrene	11 (7.8)	5 (45.45)	6 (54.55)	2.10 [0.38-11.6, p=0.40]	2.52 [0.26-24.01, p=0.42]
**Comorbid condition**					
Hypertension	79 (56)	41 (51.90)	38 (48.10)	1.35 [0.57-3.21, p=0.5]	1.69 [0.62-4.59, p=0.30]
Cardiac disease	43 (30.5)	26 (60.47)	17 (39.53)	0.74 [0.33-1.67, p=0.46]	0.58 [0.22-1.5, p=0.26]
Renal disease	33 (23.4)	20 (60.61)	13 (39.39)	0.74 [0.29-1.91, p=0.54]	0.55 [0.18-1.68, p=0.30]
Eye problem	70 (49.6)	40 (57.14)	30 (42.86)	0.73 [0.30-1.79, p=0.5]	0.72 [0.26-1.99, p=0.53]
Periodontal (gum) disease	31 (22)	15 (48.39)	16 (51.61)	1.61 [0.66-3.95, p=0.3]	1.84 [0.65-5.2, p=0.25]
Other infection	32 (22.7)	21 (65.63)	11 (34.38)	0.5 [0.20-1.2, p=0.12]	0.67 [0.24-1.91, p=0.46]
No other comorbid conditions	29 (20.57)	17 (58.62)	12 (41.38)	0.7 [0.19-2.3, p=0.52]	0.69 [0.17-2.81, p=0.61]

**Types of Diabetic treatment**					
Diets Only	5 (3.55)	4 (80)	1 (20)	Ref	Ref
Insulin Only	4 (2.84)	2 (50)	2 (50)	4.0 [0.21-75.7, p=0.36]	1.76 [0.06-53.75, p=0.75]
Oral Hypoglycemic Tablets Only	19 (13.48)	10 (52.63)	9 (47.37)	3.60 [0.34-38.5, p=0.29]	1.19 [0.09-16.35, p=0.9]
Diets and Insulin	33 (23.4)	20 (60.61)	13 (39.39)	2.60 [0.26-25.9, p=0.42]	1.15 [0.09-14.92, p=0.92]
Diets and Oral Hypoglycemic Tablets	44 (31.21)	22 (50)	22 (50)	4.0 [0.41-38.7, p=0.23]	1.73 [0.14-20.9, p=0.67]
Insulin and Oral Hypoglycemic Tablets	6 (4.26)	2 (33.33)	4 (66.67)	8.0 [0.50-127.9, p=0.14]	2.69 [0.13-56.28, p=0.52]
Diet, Insulin and Hypoglycemic Tablets	25 (17.73)	14 (56)	11 (44)	3.14 [0.31-32.3, p=0.34]	1.29 [0.09-17.54, p=0.85]
No treatment	5 (3.55)	4 (80)	1 (20)	1.0 [0.05-22.2, p=1.0]	0.13 [0.003-5.63, p=0.30]
**Exercise Status**					
No	127 (90.07)	69 (54.33)	58 (45.67)	Ref	Ref
Yes	14 (9.93)	9 (64.29)	5 (35.71)	0.66 [0.21-2.08, p=0.48]	0.50 [0.12-2.08, p=0.34]
**Wound Dressing Applied**					
No	12 (8.51)	7 (58.33)	5 (41.67)	Ref	Ref
Yes	129 (91.49)	71 (55.04)	58 (44.96)	1.14 [0.35-3.79, p=0.83]	0.46 [0.09-2.38, p=0.35]

Note: **PsA** = *Pseudomonas aeruginosa,* **DWIs** = Diabetes Wound Infections, **OR**= odd ratio * = statistically significant difference

Antibiotics susceptibility profile

Table 4 summarizes the antimicrobial susceptibility profile of *P. aeruginosa* isolates. High resistance rates were observed for aztreonam (89%) and ceftazidime (84%), followed by cefepime (81%) and tobramycin (86%). Moderate resistance was noted for amikacin (75%), ciprofloxacin (79%), and levofloxacin (79%), while lower resistance rates were observed for gentamicin (62%) and norfloxacin (56%).

Among carbapenems, resistance to imipenem and meropenem was 52% and 41%, respectively. Notably, colistin demonstrated the lowest resistance rate (37%) and the highest sensitivity (63%) among all tested antibiotics. Overall, *P. aeruginosa* isolates exhibited high levels of multidrug resistance, with relatively better susceptibility to colistin and carbapenems compared to other antibiotic classes.

Table 4: Antibiotics susceptibility profile among *P. aeruginosa* bacteria

Category	**Antibiotics /µg**	**Resistance No. (%)**	**Sensitive No. (%)**
Monobactam	Aztreonam (ATM 30)	56 (89)	7 (11)
Carbapenem	Imipenem (IPM 10)	33 (52)	30 (48)
	Meropenem (MEM 10)	26 (41)	37 (59)
3rd generation Cephalosporins	Ceftazidime (CAZ 30)	53 (84)	10 (16)
4th generation Cephalosporins	Cefepime (FEP 10)	51 (81)	12 (19)
Aminoglycosides	Amikacin (AK 10)	47 (75)	16 (25)
	Gentamicin (CN 10)	39 (62)	24 (38)

	Tobramycin (TOB 10)	54 (86)	9 (14)
Fluoroquinolones	Ciprofloxacin (CIP 10)	50 (79)	13 (21)
	Norfloxacin (NOR 30)	35 (56)	28 (44)
	levofloxacin (LEV 5)	50 (79)	13 (21)
Polypeptides	Colistin (CT 10)	23 (37)	40 (63)

Molecular identification

All *Pseudomonas aeruginosa* isolates were confirmed by PCR amplification of the 16S rDNA gene, producing a single band of 956 base pairs (Fig 1).

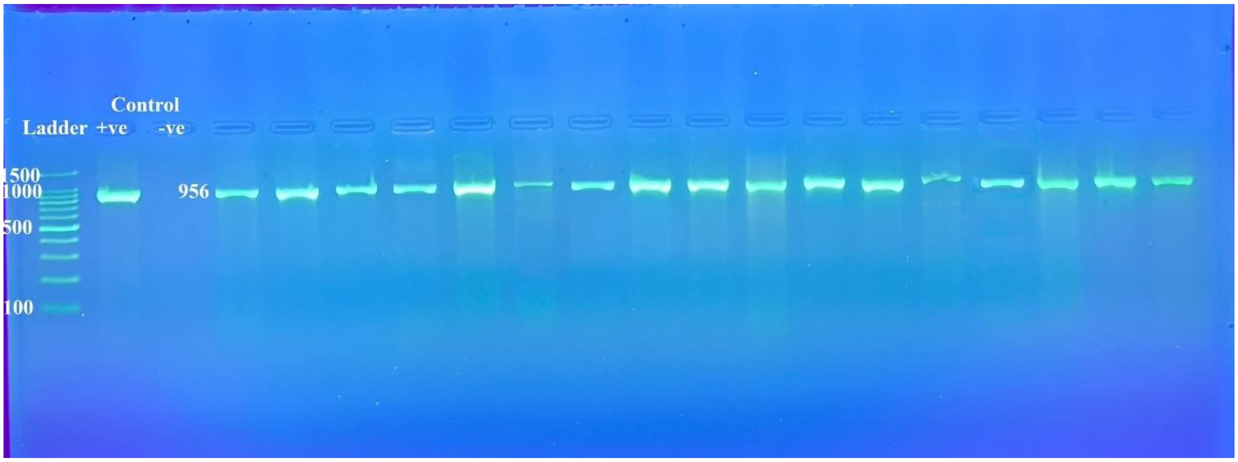

Fig 1: The amplification of PCR for species identification of P. aeruginosa using 16S rDNA primer. *The left lane shows the DNA ladder (100–1500 bp). The positive control (+ve) displays a clear band at 956 bp, while the negative control (−ve) shows no amplification. All tested isolates exhibit a distinct single band at approximately 956 bp, confirming the successful amplification of the target gene and molecular identification of* P. aeruginosa*.*

Discussion

Patients with diabetes mellitus represent a major risk group for skin wound infections, which may be caused by a variety of microorganisms, including *P. aeruginosa*. Previous studies on *P. aeruginosa* in diabetic patients have primarily focused on diabetic foot infections (DFIs). In contrast, the present study investigated P. *aeruginosa*–associated wound infections across all anatomical sites, reporting a prevalence of 63 cases (44.68%) among 141 patients. The identification of *P.s aeruginosa* in this study was confirmed using conventional biochemical tests, the Vitek®2 system, and 16S rDNA PCR amplification. The use of PCR provided a high level of specificity and accuracy in bacterial identification, thereby strengthening the reliability of the laboratory findings. This molecular confirmation reduces the possibility of misidentification that may occur with conventional methods alone and ensures more precise detection of *P. aeruginosa* in diabetic wound infections. Therefore, the combination of phenotypic and molecular techniques improves the validity of the reported prevalence and supports the robustness of the study results. This finding is consistent with a study conducted in Duhok city, in which all bacterial isolates were similarly confirmed using the 16S rDNA gene [23].

The prevalence of *P. aeruginosa* among DFU observed in this study is comparable to reports from Najaf, Iraq (34.4%) [9], Iran (35%) [24], and various regions of India, including North (23.7%), South (29.8%), North-East (11.89%), East (11.7%) and West India (27%) [25]. Similarly, a study conducted in Erbil, Iraq, reported that 53 of 150 DFU samples (35.33%) were positive with *P. aeruginosa* [11]. In the present study, PsA DWIs were less frequent than non-PsA DWIs, which contrasts with findings from a study in Anhui Province, China, where *P. aeruginosa* accounted for only 9% of infections compared to 91% for non-*P. aeruginosa* pathogens [26]. These variations may be attributed to differences in causative microorganisms, geographic location, and the severity and types of infections [27].

The current study found a higher prevalence rate of DM among females compared to males. Additionally, a higher rate of PsA DWIs was observed in the 50-59 age groups. A comparable study conducted in Kermanshah Province, Iran, reported a female prevalence of 57.4% and a male prevalence of 42.6% [1]. In contrast, a previous study in Najaf City, Iraq, found a lower proportion of females (44.3%) compared to males (55.7%) among 282 DM patients with foot wound infections [9]. Furthermore, another study in Iraq reported a male to female ratio of 2.5:1, while demonstrating a similar age distribution, with the highest incidence of DFI occurring in the 51-60 age groups [28]. The higher proportion of cases among females in the present study may be attributed to cultural variations influencing access to healthcare services [1].

The present study indicated that individuals residing in urban areas are more likely to develop wound infection compared to those living in rural areas. This study is consistent with a study conducted in Ethiopia, by Molla et al (2023), which reported a higher prevalence of DFI in urban population (62.4%) compared to rural (37.6%) [29]. Similarly, a study carried out in Erbil City, Iraq, demonstrated that

infection rates were higher in urban areas (54%) than in rural areas (46%) [11]. These variations may be attributed to differences in study populations and methodologies. Furthermore, several indicators used to assess socioeconomic status at the neighborhood level may provide insight into the environmental and social factors influencing the risk of infection among residents [30].

Conversely, obese individual exhibited the highest rates of infection compared to those who were overweight or normal weight. However, the prevalence rate of PsA DWIs was highest among individuals with normal weight, followed by overweight and obese groups. This finding is consistent with a study conducted in Iraq, which reported a higher incidence of diabetic foot infections (DFIs) among obese participants [5]. Additionally, a study from the United States found that the mean body mass index (BMI) among DFI patients with and without *P. aeruginosa* infection was 33.6% and 30.6%, respectively [31]. Over recent decades, obesity rates have increased dramatically, leading the World Health Organization to classify as a global epidemic and a major threat to public health [15]. Obesity is associated with several comorbid conditions, such as atherosclerosis, which may contribute to the development of diabetic foot ulcers (DFUs) and subsequent infections [5].

Educational level varied among participants, with illiterate individuals showing a higher likelihood of infection compared to other groups. In all categories, the prevalence of *P. aeruginosa* diabetic wound infections (DWIs) was lower than or equal to that of non-*P. aeruginosa* DWIs. To date, no studies have specifically examined the relationship between educational level and *P. aeruginosa* DWIs. Consistent with the present findings, a study conducted in Colombia reported that the majority of individuals with DM had not completed primary education [32]. Similarly, a study from Kirkuk City, Iraq, reported an illiteracy rate of 24%, compared to only 4% among those with higher education [4]. In contrast, several other studies have reported conflicting results, indicating higher rates of diabetic foot infections (DFIs) among individuals with higher educational attainment, including those with high school and university education [5, 33] while lower rates were observed among illiterate patients [29].

Moreover, evidence suggests that individuals with lower levels of education are more likely to develop DM and experience its complications [30]. These findings indicated that the level of education is important in taking care of metabolic diseases and the promotion of health-protective behaviors [4]. Patients with limited education may have reduced awareness regarding glycemic control and proper wound care practices, increasing their susceptibility to infection.

This study is partly consistent with findings from Iraq, where employment status was distributed as follows: housewives (37.6%), employees (12.4%), daily laborers (22.8%), and pensioners (27%) [5]. Similarly, a study from Ethiopia reported housewives (19%), government employees (26.8%), daily laborers (5.3%), pensioners (5%), unemployed (3.8%), and others (farmers and merchants) [29]. In our study, the highest rates of PsA DWIs were observed among non-working, non-occupational, and uneducated patients of both sexes.

Smoking patterns in our study were similar to a previous report, in which most patients were non-smokers (74.9%), followed by smokers (1.6%) and ex-smokers (9.5%) [5]. Another study reported a higher rate of PsA in foot infections compared with non-PsA cases (37% vs. 19%) [8]. In our study, no significant association was found between smoking status and PsA DWIs, although non-smokers had higher rates than current and former smokers.

The distribution of family history of diabetes in our study differs from findings in Iraq, where rates among DFI patients were negative (47%), positive (36%), and unknown (16.2%) [5]. Similarly, other studies reported negative rates of 62.2% and 72.7%, and positive rates of 37.8% and 27.3%, respectively [29,34]. Evidence consistently shows that individuals with a family history of diabetes are at higher risk of developing the disease, likely due to genetic factors [34,35].

The mean HbA1c in our study was comparable to previous reports (8.3 ± 2.2 [24], 8.94 ± 2.1 [36], and 8.61 ± 2.24 [37]), although one study reported a higher mean (10.26 ± 2.0) [5]. Similarly, a U.S. study reported a mean HbA1c of 8.3% [8]. HbA1c was higher in patients with PsA DFI than in non-PsA DFI (8.5% vs. 7.3%). Chronic hyperglycemia impairs wound healing, increases wound size, and is associated with complications and mortality [38,39]. Therefore, maintaining optimal HbA1c levels is essential for preventing diabetic foot complications.

Several studies have reported the mean ± SD of diabetes duration, including 14.5 ± 4.5 years [24], 13.6 ± 8.5 years [36], and 13.54 ± 6.98 years [5]. One study reported that 40.3% of patients had a duration ≤10 years, while 59.7% had >10 years [33], and another reported 11.1% [37]. The mean ulcer duration among DFU patients has been reported as 14.5 ± 4.5 months [24] and 1.8 months [37]. In contrast to studies focusing solely on foot infections, our study included multiple anatomical sites. Toe infections were most common (44%), including 30% PsA and 45% non-PsA DFI. The lateral plantar region accounted for 29% (22% PsA, 30% non-PsA), the heel for 16% (19% PsA, 16% non-PsA), and other sites for 14% (37% PsA, 11% non-PsA). Unspecified sites comprised 9% (4% PsA, 9% non-PsA) [8]. A significant association was observed between wound location and DM ($p = 0.001$), except for foot-related sites.

Based on infection severity, DFIs in our study were mainly ulcers (70%), followed by abscesses (21%) and combined ulcer–abscess lesions (10%). PsA DFIs were more frequent in ulcers (82%) than in non-PsA cases (68%), whereas abscesses and mixed lesions were more common in non-PsA DFIs [8]. Other studies reported varying distributions, including ulcers (44%), cellulitis (20%), abscesses (32%), gangrene (24%), and neuropathic ulcers (4%) [7], while another found ulcers in 50.2% of cases [37]. Overall, wound infection is a common complication of diabetes, often resulting from ulceration and inflammation, and may progress to gangrene and lower limb amputation, contributing to significant morbidity, hospitalization, and mortality [7].

Several studies have examined DFU and associated complications. In one study of 54 DFU cases, hypertension, retinopathy, nephropathy, neuropathy, and osteomyelitis were reported in 11, 8, 11, 24, and 10 patients, respectively [24]. Another study (2023) found that 62.2% of patients had

comorbidities, most commonly hypertension (30.8%), followed by combinations of cardiovascular, renal, and other chronic diseases, while 37.8% had none [29]. Similarly, another report showed hypertension (43.2%) as the most frequent comorbidity, followed by hypercholesterolemia (10.7%). Complications were present in 16% of cases, including nephropathy (1.9%), retinopathy (5.8%), neuropathy (4.9%), and other conditions, while 84% had no complications [33]. Ultimately, poor blood supply, infection, and neuropathy may progress to tissue necrosis, osteomyelitis, gangrene, and amputation [7,39]. The presence of multiple (≥3) complications in DM patients with wound infection is an indicator of increased amputation risk. Therefore, improved education, early detection, and timely management are essential to reduce major amputations [26].

Regarding treatment methods, one study reported that among DM patients, 26.6% used oral hypoglycemic agents, 43.6% insulin, 29.4% both insulin and oral agents, 0.3% other treatments, and 3.3% received no treatment [37]. Another study reported diet alone (10.7%), oral hypoglycemic agents (55.8%), insulin alone (8.3%), combined therapy (12.6%), and 12.6% with no treatment [29]. Similarly, oral hypoglycemic agents, insulin, and combination therapy were reported in 42.9%, 31.6%, and 25.6% of patients, respectively [29]. In addition, 43.2% of DM patients engaged in exercise, while 56.8% did not [33]. The risk of developing type 2 diabetes can be reduced through a healthy diet and regular exercise. Improved insulin sensitivity reduces hepatic glucose production, leading to lower HbA1c levels, which in turn decreases the risk of diabetes-related complications [40].

Most patients are managed with wound dressing and debridement [7], and about two-thirds of DM patients with wound infection receive antimicrobial dressings [37]. Selection of wound dressings should follow best practice recommendations (BPRs) and clinical practice guidelines (CPGs) to ensure appropriate moisture balance, optimal wound care, and support for surgical cleansing. Antimicrobial dressings may also promote faster healing compared with non-antimicrobial dressings [6].

The multivariate logistic regression results of the present study differ from previous reports, which identified various independent predictors of infection in DFU/DFI patients. One study reported that age >65 years (OR=1.242, 95% CI: 1.251–2.114, p=0.001) and HbA1c >7% (OR=2.155, 95% CI: 1.287–2.946, p=0.032) were the only significant factors associated with infection in DFUs [24]. In another study, smoking and insulin use were significant in unadjusted analysis (OR=2.6, 95% CI: 1.1–6, p=0.022 and OR=0.21, 95% CI: 0.08–5.2, p<0.001, respectively), but they lost significance or were excluded in the final regression model [8]. Furthermore, a study on DFU-related amputation found that infection site was associated with lower limb amputation in both univariate (OR=0.347, 95% CI: 0.790–0.926, p<0.001) and multivariate analyses (OR=0.816, 95% CI: 0.724–0.919, p<0.001) [26].

This study has several limitations. Its cross-sectional design limits the ability to establish causal or temporal relationships between risk factors and *P. aeruginosa* infection. In addition, being conducted in a single province with a relatively small sample size may restrict the generalizability of the findings. However, the study also has important strengths. Clinical wound samples were collected over a 12-month period, minimizing seasonal variation. Samples were obtained from multiple hospitals and private clinics in Zakho and Duhok, improving representativeness. Identification of P. aeruginosa was confirmed using biochemical methods and 16S rDNA PCR, ensuring high diagnostic accuracy. The study also included a broad range of demographic, clinical, and behavioral variables (e.g., age, BMI, smoking, wound characteristics, and glycemic control), allowing a comprehensive assessment of potential risk factors. Finally, antimicrobial susceptibility testing was performed using CLSI-standard disc diffusion methods, ensuring reliable and comparable resistance data.

Conclusion

Diabetic patients with wound infections represent complex cases that may lead to major complications. In the present study, most sociodemographic factors, including age, sex, residence, education level, smoking status, and family history of diabetes, were not significantly associated with *P. aeruginosa* diabetic wound infections in both unadjusted and adjusted analyses. BMI showed a significant association in the unadjusted model for overweight patients (p = 0.019), but this was not maintained after adjustment. In the multivariate analysis, only occupational status showed a significant association, where government employees (p = 0.046) and pensioners (p = 0.047) had a lower likelihood of *P. aeruginosa* infection compared with daily laborers. Overall, sociodemographic characteristics had limited independent predictive value for *P. aeruginosa* DWIs, except for occupational status. In this study, most clinical variables—including HbA1c level, duration of diabetes, ulcer history, comorbidities, treatment type, exercise status, and wound dressing—were not significantly associated with *P. aeruginosa* diabetic wound infections in both unadjusted and adjusted logistic regression analyses. The only significant independent predictor was wound location, where infections involving the leg region showed a significantly higher risk of *P. aeruginosa* infection (adjusted OR = 3.94, p = 0.02). Overall, these findings suggest that anatomical site of infection plays a key role in *P. aeruginosa* occurrence, while other clinical and behavioral factors showed no independent association. *P. aeruginosa* isolates showed high levels of multidrug resistance, with the highest resistance observed against aztreonam, ceftazidime, and tobramycin. Carbapenems showed moderate resistance, while colistin remained the most effective antibiotic with the highest sensitivity rate.

Therefore, early detection and prompt management of wounds are essential to prevent progression to deep tissue infection. Individuals with a family history of diabetes should be particularly aware of the risk of earlier onset, especially in the presence of high BMI. Improving patient outcomes also depends on strengthening the education level and training of healthcare professionals, particularly nurses, who play a key role in patient education on wound care and complication prevention. Strengthening infection control practices, promoting rational antibiotic use, and improving patient education on wound care are essential to reduce complications and antimicrobial resistance in diabetic patients. In addition, further clinical

trials are needed to guide the selection of appropriate antimicrobial agents for wound dressings to ensure optimal patient care

Acknowledgments

We would like to express sincere gratitude to all participants who took part in this study.

Conflict of Interest

The authors declared no conflict of interest regarding study content.

Financial Disclosure

The study did not receive any financial support

References

[1] Ghobadi A, Sarbarzeh PA, Jalilian M, Abdi A, Manouchehri S. Evaluation of Factors Affecting the Severity of Diabetic Foot Ulcer in Patients with Diabetes Referred to a Diabetes Centre in Kermanshah. Dovepress. 2020; 13: 693–703. http://doi.org/10.2147/DMSO.S242431

[2] Sivanmaliappan TS, Sevanan M. Antimicrobial Susceptibility Patterns of *Pseudomonas aeruginosa* from Diabetes Patients with Foot Ulcers. International Journal ofMicrobiology. 2011; 2011: 1–4. doi: 10.1155/2011/605195.

[3] Zhang J, Deng Y, Wan Y, Wang J, Xu J. Diabetes duration and types of diabetes treatment in data-driven clusters of patients with diabetes, Frontiers in Endocrinol. 2022; 10 (3389): 1–12. doi: 10.3389/fendo.2022.994836.

[4] Amen MR, Kumait AS, Jaafr AY. Risk Factors for Diabetic Foot Ulceration Among Patients: A Cross-Sectional Study in Kirkuk City , Iraq. Journal of Pioneering Medical Sciences.2025; 14 (07): 112–116, 2025.

[5] Mohammed AG, Al-waeli DK, Odhaib SA, Altemimi MT. Assessing the Features of Diabetic Foot Ulcers among Individuals with Type 2 Diabetes Mellitus in Thi Qar, Iraq. F1000 Research. 2025; 13 (539): 1–14.

[6] Ivory JD, Gethin G, Mcintosh C. Antimicrobial dressings for treating local infection in patients with diabetic foot ulcers. The Diabetic Foot Journal. 2018; 21 (3): 155–159.

[7] Kothari B. Clinical study and surgical management of diabetic foot: An observational study. International Journal of Surgery Science. 2022; 6 (1): 130–133.

[8] Veve MP, Mercuro NJ, Sangiovanni RJ, Santarossa M, Patel N. Prevalence and Predictors of *Pseudomonas aeruginosa* Among Hospitalized Patients With Diabetic Foot Infections. Open Forum Infectious Diseases. 2022; 9 (7): 1–8.

[9] Al-Khudhairy MK, Al-Shammari MMM. Prevalence of metallo-β-lactamase–producing *Pseudomonas aeruginosa* isolated from diabetic foot infections in Iraq. New Microbe and New Infect. 2000; 35 (100661): 1–6.

[10] Al Abbas LA, Khaddam W, Shreibati F. INVESTIGATING BACTERIA ISOLATED FROM DIABETIC FOOT ULCERS AND STUDYING THEIR SENSITIVITY TO ANTIBIOTICS –SYRIA. Bulletin of

Pharmaceutical Sciences Assiut University. 2022; 45 (1): 451–458.

[11] Khader KA, Raad AZ. Molecular detection of toxA and lasB virulence genes of *Pseudomonas aeruginosa* strains isolated from Diabetic Foot Ulcers in Erbil, Iraq. Journal of Applied and Natural Science. 2025; 17 (3): 1386–1392.

[12] Muhammed S, Aziz AD, Ahmed MJ. Microbial Identification And Antibiotic Resistance Patterns In Diabetic Foot Ulcers From Patients In Erbil, Kurdistan Region, Iraq. The Review of DIABETIC STUDIES. 2025; 21: 634–644.

[13] Qadir AN, Mahmoud BM, Mahwi TO, Mohammed D, Arif R, Mahmood SO. Prevalence of Microorganisms and Antibiotic Sensitivity Among Patients with Diabetic Foot Ulcer in Sulaimani City, Iraq. Hospital Practices and Research. 2020; 5 (2): 56–63. doi: 10.34172/hpr.2020.11.

[14] Horcajada JP, Montero M, Oliver A, Sorlí L, Luque S, Gómez-Zorrilla S, et al. Epidemiology and Treatment of Multidrug-Resistant and Extensively Drug-Resistant. Clinical Microbiology Reviews. 2019; 32 (4): 1–52.

[15] Kolimechkov S, Petrov L. The Body Mass Index : A Systematic Review. Journal of Exercise Physiology and Health. 2020; 3 (2): 21–27.

[16] Apridamayanti P, Meilinasary KA, Sari R. Antibiotic Sensitivity in *Pseudomonas aeruginosa* of Diabetic Patient ' s Foot Ulcer. Pharm Sci Res. 2016; 3 (2): 80–87.

[17] Callahan A, Liam P. A Clinical Review of Necrotic Skin Lesions as Presentations of Systemic Infection. Preprints.org. 2025: 0–17. doi: 10.20944/preprints202507.1668.v1.

[18] Alexander SK, Strete D. Microbiology: A Photographic Atlas For The Laboratory. USA: Daryl F, 2001.

[19] Cheesbrough M. District Laboratory Practice in Tropical Countries. Second edition. New York, USA: Cambridge University Press, 2006.

[20] Spilker T, Coenye T, Vandamme P, Lipuma JJ. PCR-Based Assay for Differentiation of *Pseudomonas aeruginosa* from Other Pseudomonas Species Recovered from Cystic Fibrosis Patients. JOURNAL OF CLINICAL MICROBIOLOGY. 2004; 42 (5): 2074–2079. doi: 10.1128/JCM.42.5.2074.

[21] Polse RF. Bacteriological and Molecular Identification of *Pseudomonas aeruginosa* fron Burns and Wound Infection in Duhok Province/Kurdistan Region/Iraq. Doctoral dissertation, University of Zakho, 2024.

[22] Ausubel FM, Brent R, Kingston RE, Moore DD, Seidman JG, Smith JA, et al. Current Protocols in Molecular Biology. Ringbou. Wiley S, 2003.

[23] Polse RF, Khalid HM, Mero WMS. Distribution of blaOXA-10, blaPER-1, and blaSHV genes in ESBL-producing *Pseudomonas aeruginosa* strains isolated from burn patients. Scientific Reports. 2023; 13 (18402): 1–8. doi: 10.1038/s41598-023-45417-4.

[24] Samani S, Parsa H. Microbiological Features and Risk Factors in Patients With Diabetic Foot Ulcers. Index Wounds. 2015; 27 (11): 308–312.

[25] Chatterjee D, Sivashanmugam K. Insights on MDR Mechanism of *Pseudomonas aeruginosa* with Emphasis on Diabetic Foot Ulcer in the Indian Subcontinent. Journal of Pure and Applied Microbiolgy. 2024; 18 (2): 837–852. doi: 10.22207/JPAM.18.2.08.

[26] Yao Y, Chen L, Qian Y. Age Characteristics of Patients With Type 2 Diabetic Foot Ulcers and Predictive Risk Factors for Lower Limb Amputation: A Population-Based Retrospective Study. Journal of Diabetes Research. 2024; 2024 (1): 1–8. doi: 10.1155/jdr/2380337.

[27] Garousi M, Monazamitabar S, Mirazi H, Farrokhi Z, Khaledi A, ShakerimoghaddamA. Epidemiology of *Pseudomonas aeruginosa* in diabetic foot infections: a global systematic review and meta-analysi. Germs. 2-23; 13 (4): 362–372.

[28] Mahmoud HM, Abdul S, Abid A, Qadir D. Prevalence of bacterial types in Wagner grade three of diabetic foot ulcer. Iraqi Journal of Science. 2014; 55 (3): 1232–1235.

[29] Molla IB, Berhie MA, Debele KA, Germossa GN, Hailu FB. Persons with Diabetes' Perceptions of Family Burden and Associated Factors. Journal of Diabetes Research. 2023; 2023: 1–8.

[30] Schmidt BM, Huang Y, Banerjee M, Hayek SS, Pop-busui R. Residential Address Amplifies Health Disparities and Risk of Infection in Individuals With Diabetic Foot Ulcers Residential Address Amplifies Health Disparities and Risk of Infection in Individuals With Diabetic Foot Ulcers. Diabetes Care. 2024; 47: 508–515.

[31] J. Lau, J. Huang, and Z. K. Escobar, "Antipseudomonal Antibiotics in Diabetic Foot Infections : A Practical Perspective From a Community Hospital," Open Forum Infect. Dis., vol. 11, no. 6, pp. 1–4, 2024, doi: 10.1093/ofid/ofae258.

[32] Ramirez-Perdomo C, Perdomo-Romero A, Rodríguez-Vélez M. Knowledge and practices for the prevention of the diabetic foot. Rev Gaúcha Enferm. 2019; 40 (e20180161): 1–7.

[33] Ghanem SS, Abdulkreem EM, Alfurayh MS, Ahmed AA, Rikabi HA. The Role of HbA1c in the Follow-Up and Control of Diabetes Mellitus. Cureus. 2025; 17 (2): 1–7. doi: 10.7759/cureus.78357.

[34] Erasmus RT, Blanco EB, Okesina AB, Arana JM, Gqweta Z, Matsha T. Importance of family history in type 2 black South African diabetic patients. Postgraduate Medical Journal. 2001;77:323–325. doi: 10.1136/pmj.77.907.323.

[35] Peng H, Wang M, Guo H, Hou T, Li Y, Zhang H, etal. Parental Transmission of Type 2 Diabetes Risk in Offspring: A Prospective Family-Based Cohort Study in Northern China. Nutrients. 2025; 17 (1361): 1–12.

[36] Alharithy MK, Alobaylan MM, Alsugai ZO, Alswat KA. IMPACT OF FAMILY HISTORY OF DIABETES ON DIABETES CONTROL AND COMPLICATIONS. ENDOCRINE PRACTICE. 2018; 24 (9): 773–779. doi: 10.4158/EP-2018-0071.

[37] Ndosi M, Wright-Hughes A, Brown S, Backhouse M, Lipsky BA, Bhogal M, et al. Prognosis of the infected diabetic foot ulcer: a 12-month prospective observational study. Diabetic Medicine. 2018; 35: 78–88. doi: 10.1111/dme.13537.

[38] Fesseha BK, Abularrage CJ, Hines KF, Sherman R, Frost P, Langan S, et al. Association of

Hemoglobin A 1c and Wound Healing in Diabetic Foot Ulcers. Diabetes Care. 2018; 41: 1478–1485. doi: 10.2337/dc17-1683.

[39] Dogan M, Onar LC, Aydin B, Gumustas SA. Is high level of hemoglobin A1C an indicator for extended period of antibiotherapy in diabetic foot ulcers? Infectious Diseases & Microbiology. 2019; 6 (1): 21–27. doi: 10.14744/nci.2018.25582.

[40] Amer BE, Abdelgalil MS, Hamad AA, Abdelsayed K, Elaraby A, Abozaid AM, et al. Metformin plus lifestyle interventions versus lifestyle interventions alone for the delay or prevention of type 2 diabetes in individuals with prediabetes : a meta-analysis of randomized controlled trials. Diabetology & Metabolic Syndrome. 2024; 16 (273): 1–14.

Click here to access/download
Supporting Information
S1_Raw-Images.jpeg

Prevalence and associated risk factors of *Pseudomonas aeruginosa* infection in Diabetic wounds in Duhok province, Iraq

Marwa S. Ibrahim[1] and Ibrahim A. Naqid[2*]

[1]Department of Medical Laboratory Technology, College of Health and Medical Techniques-Shekhan, Duhok Polytechnic University, Duhok, Kurdistan Region, Iraq

Department of Biomedical Sciences, College of Medicine, University of Zakho, Kurdistan Region, Iraq

Abstract

Background and aims

Diabetes mellitus is a chronic metabolic disorder and a major global health problem, predisposing patients to wound infections, with *Pseudomonas aeruginosa* being a frequent and resistant pathogen. This study aimed to identify *P. aeruginosa*, assess associated risk factors, and determine its antibiotic susceptibility pattern in diabetic wound infections in Duhok Province, Kurdistan Region, Iraq.

Methods

This cross-sectional study was conducted from June 2024 to July 2025 in Duhok Province, Kurdistan Region, Iraq. A total of 141 wound swab samples were collected from diabetic patients aged ≥18 years. *P. aeruginosa* was identified using biochemical tests, Vitek®2, and 16S rDNA PCR. Antimicrobial susceptibility testing was performed by the disc diffusion method on Mueller–Hinton agar, and data were analyzed using GraphPad Prism software.

Results

Out of 141 wound infection samples collected from diabetic patients, *P. aeruginosa* was isolated in 44.68% of cases. Overall, sociodemographic factors showed no significant association with PsA infection, except for occupational status in the multivariable analysis 0.12 [0.02-0.97, p=0.046] and overweight 0.29 [0.10-0.82, p=0.019]. Clinically, most variables including HbA1c, duration of diabetes, comorbidities, treatment type, and wound characteristics were not significantly associated with PsA infection. The only significant predictor was wound location, with leg-region infections showing a higher risk of PsA 3.94 [1.24-12.5, p=0.02]. *P. aeruginosa* isolates showed high levels of multidrug resistance, especially to aztreonam, ceftazidime, cefepime, and tobramycin. Colistin showed the lowest resistance and highest sensitivity, followed by relatively better susceptibility to carbapenems.

Conclusion

P. aeruginosa is a common cause of wound infections in patients with diabetes mellitus, with its prevalence significantly associated with body mass index, occupational status and wound location, with leg-region wounds showing a higher risk. *P. aeruginosa* isolates demonstrated high multidrug resistance, although colistin and carbapenems retained relatively better activity. Early detection and appropriate antibiotic selection are essential to reduce complications and improve patient outcomes.

Keywords:
Pseudomonas aeruginosa; Diabetic wound infection; Prevalence; Risk factors; Antibiotic susceptibility; Duhok Province; Iraq

Introduction

Diabetes mellitus (DM) is a chronic metabolic disorder characterized by persistent hyperglycemia and disturbances in carbohydrate and lipid metabolism, resulting in insulin resistance [1]. It is recognized as a major global public health problem and an emerging epidemic, contributing significantly to morbidity and disability in both developing and developed countries [1, 2]. DM is classified into type 1 and type 2 diabetes [3]. Patients with type 2 diabetes are at increased risk of developing multiple complications, including hypertension, cardiovascular disease, renal failure, cerebrovascular accidents, retinopathy, and neuropathy [4,5]. In addition to comorbidities, several factors influence the risk and severity of infection. These include local factors such as poor tissue perfusion, presence of foreign bodies, necrosis, wound location and size, and poorly controlled diabetes. Behavioral factors such as malnutrition, non-adherence to treatment plans, and smoking, as well as social determinants such as educational level and socioeconomic status, also play important roles [6].

Furthermore, peripheral ischemic disease is a frequent and severe complication that can lead to ulceration and wound infection, particularly diabetic foot ulcers (DFU) [1]. Ulceration is the most common clinical manifestation of wound infections, followed by cellulitis and gangrene. The dorsum of the foot is the most commonly affected site, followed by the forefoot and toes [7]. This condition may further progress to osteomyelitis [8]. Among DM patients who develop foot ulcers, approximately 20–30% require minor amputation, while about 5% undergo major amputation [6].

Chronic wound infections are typically polymicrobial, and microbial colonization of a localized wound can lead to extensive replication on the wound surface. As the microbial load increases, pathogens may penetrate deeper tissues, resulting in clinical signs and symptoms in diabetic patients [6]. Both Gram-positive and Gram-negative bacteria can cause wound infections, with *P. aeruginosa* being one of the most common pathogens in diabetic patients [9, 10]. This organism can cause severe infections in multiple sites, including pneumonia, urinary tract infections, keratitis, and soft tissue infections of open wounds, particularly diabetic foot ulcers and burn injuries [11]. It produces multiple toxins, evades phagocytosis, and exhibits broad antibiotic resistance, making it a highly virulent and difficult-to-treat pathogen [2]. A previous study in Erbil, Iraq reported a prevalence of *P. aeruginosa* in diabetic foot infections of 35.33% [11]. Another study conducted in Erbil [12] found *P. aeruginosa* to be the most common Gram-negative bacterium, accounting for 21.43% of isolates. In contrast, a study in Sulaymaniyah reported a lower prevalence of *P. aeruginosa* among diabetic foot infections (8.5%) [13].

Antibiotic resistance in *P. aeruginosa* has become a major global public health concern, particularly due to the limited availability of effective alternative treatment. Infections caused by this pathogen significantly contribute to morbidity and mortality worldwide. According to the European Centre for Disease Prevention and Control, 13.7% of *P. aeruginosa* isolates are resistant to at least three

antimicrobial groups, while 5.5% are resistant to all five groups under surveillance. This highlights the growing challenge of multidrug resistance and the need for effective antibiotic stewardship and infection control measures [14]. The declining sensitivity of *P. aeruginosa* to commonly used antibiotics underscores the urgent need to prevent antibiotic overuse and to strengthen antimicrobial stewardship efforts, which may help curb resistance in the future [10]. Furthermore, antibiotic selection for the treating aeruginosa infections is often inconsistent, highlighting the critical importance of choosing appropriate, evidence-based therapeutic regimens [11].

Most previous studies in the Kurdistan Region of Iraq have focused mainly on *P. aeruginosa* in diabetic foot ulcers. Therefore, the present study aimed to isolate P. aeruginosa from wound infections at various anatomical sites among diabetic patients in different hospitals in Duhok Province, Kurdistan Region, Iraq. In addition, it aimed to identify key risk factors associated with *P. aeruginosa* wound infections and determine the antimicrobial susceptibility patterns of the isolates.

Material and method

Study design, and sample collection

This cross-sectional study was conducted in Duhok Province, Kurdistan Region, Iraq, from July 2024 to July 2025. A total of 141 wound samples were collected from diabetic outpatients (77 females, 64 males) aged ≥18 years at Zakho General Hospital and Azadi Teaching Hospital using a **convenience sampling** approach, based on the availability of eligible patients during the study period. The sample size of 141 was based on all eligible diabetic wound infection cases collected during the study period in selected hospitals; therefore, a census sampling approach was applied. Wounds were first cleaned with normal saline, after which samples were obtained using sterile swabs and transported in Amies medium. All samples were cultured on appropriate media at the Microbiology Laboratory of Zakho Technical Institute. Written informed consent was obtained from all participants before sampling.

Severity of wound infection:

The severity of wound infection was classified based on the degree of tissue involvement. The severity was defined based on the degree of tissue involvement and clinical presentation, including ulcers (necrosis with erythema, pain, and discharge), abscess formation (pus collection), cellular necrosis (tissue death due to hypoxia or vascular occlusion), and gangrene (advanced necrosis with or without systemic symptoms). Poor skin care and unrecognized skin injuries may progress to chronic ulcers, facilitating microbial invasion of skin tissue. As infection extends to deeper layers, it may involve muscles, joints, and tendon sheaths, leading to pus accumulation, tissue necrosis, and abscess formation. Diabetic ulcers are open skin wounds characterized by necrosis, surrounding erythema, pain, and purulent discharge [16]. Necrosis may also result from vascular occlusion or trauma, leading to tissue hypoxia and cell death. Advanced necrosis can results in eschar formation and may progress to wet or dry gangrene, often accompanied by systemic symptoms such as fever and malaise. Wet gangrene is frequently associated with foul odor due to secondary infection [17

Body Mass Index (BMI)

Patients' weight and height were measured, and body mass index (BMI, kg/m²) was calculated and classified as underweight (<18.5), normal weight (18.5–24.9), overweight (25.0–29.9), or obese (≥30.0).[15].

Bacterial Isolation and Identification

Wound swab samples were immediately cultured on Blood, MacConkey, and Chocolate agars and incubated overnight at 37 °C. Suspected *P. aeruginosa* colonies were selected and sub-cultured on MacConkey agar to obtain pure isolates. Further characterization was performed using biochemical tests, including peptone water, methyl red, Voges–Proskauer, Simmons citrate, Triple Sugar Iron (TSI), cetrimide agar, oxidase test, and Gram staining [18]. All isolates were additionally confirmed using the automated Vitek®2 system (bioMérieux, France).

Antibiotic susceptibility test

Antimicrobial susceptibility *test* of *P. aeruginosa* isolates was performed using twelve antibiotic discs (Bioanalyses, Turkey). The disc diffusion method was performed on Mueller-Hinton agar to assess antibiotic susceptibility and resistance. After 24 hours of incubation, the diameters of the inhibition zone around each disc were measured, and compared to standard reference value. Based on the size of the inhibition zones, the isolates were interpreted as susceptible or resistant in accordance with Clinical and Laboratory Standards Institute (CLSI) guidelines (M100 35rd Edition 2025) [19].

Molecular investigation

Genomic DNA extraction

Genomic DNA of all isolated *P. aeruginosa strains* was extracted, using a commercial extraction kit (PrimePrepTM Genomic DNA Extraction Kit, GeNet Bio, Korea). Following extraction, the concentration and purity of the DNA samples were determined using a NanoDrop spectrophotometer (Thermo scientific, USA). The extracted genomic DNA was subsequently used as a template for polymerase chain reaction (PCR) amplification. All procedures were conducted at the Research Center of the Biology Department, Faculty of Science, University of Zakho.

Primers

A 16S rDNA species-specific primer set was used for the molecular identification of the 16S rDNA gene in *P. aeruginosa.* The primer sequences used were: forward (GGGGGATCTTCGGACCTCA) and reverse (TCCTTAGAGTGCCCACCCG), yielding an expected amplicon size of 956 base pairs as previously described [20]. A positive control for the 16S rDNA gene was obtained from [21] with the accession No. (OR882879).

PCR amplification and gel electrophoresis

The final volume of the PCR amplification reaction was 20 µl consisting of 8 µL of master mix (2x Taq Mix, manufacturer by GDSBio- China, Lot number: 130) 2 µL of forward primer**,** 2 µL of reverse primer**,** 2 µL of genomic DNA (25–50 ng/µL)**,** and 6 µL of PCR-grad. Following preparation of the reaction mixture, the tubes were placed into the thermal cycler for amplification. The amplification conditions are summarized in Table (1). After amplification, the PCR products were analysed by gel electrophoresis. A 1.25% (w/v) agarose gel was prepared using 1× Tris–Borate–EDTA (TBE) buffer**,** and samples were electrophoresed for 5 minutes at 45 V, followed by 30 minutes at 80 V. DNA bands were subsequently visualized under a UV transilluminator (Cleaver Scientific, UK). The procedure followed the standard protocols described by by Ausubel et al [22].

Table 1: The amplification condition of 16S rDNA PCR analysis for 16S rDNA primer

<table>
<tr><th>Initial denaturation</th><th>Denaturation</th><th>Annealing</th><th>Extension</th><th>Final extension</th><th>Reference</th></tr>
<tr><td>94 ºC</td><td>92 ºC</td><td>54 ºC</td><td>72 ºC</td><td>72 ºC</td><td rowspan="3">[20]</td></tr>
<tr><td>2 min</td><td>20 sec</td><td>20 sec</td><td>40 sec.</td><td>5 min</td></tr>
<tr><td>1 cycle</td><td colspan="3">25 cycles</td><td>1 cycle</td></tr>
</table>

Inclusion and exclusion criteria

All clinical samples obtained from diabetic patients aged ≥ 18 years who met the diagnostic criteria for wound infection and attended to hospitals or outpatients' clinics were included in the study. The exclusion criteria were as follows: (1) patients younger than 18-years of age, (2) patients who declined participation in the study and (3) patient who had received antimicrobial therapy prior to sample collection.

Ethics

Ethical approval for this study was obtained from Medical and General Health Research Ethics Committee from the Ministry of Health, Duhok Directorate General of Health, under the reference number (29052024-4-2).

Statistical analysis

raphPad Prism 10.6.1 was used for statistical analysis. Binary logistic regression was employed to calculate odds ratios (ORs), 95% confidence intervals (95% CIs), and p-values. All variables were included in a multivariate logistic regression model. A p-value < 0.05 was considered statistically significant, indicating strong evidence of a relationship between the independent variables and the outcomes under investigation.

Result

Demographic characteristics of the diabetic patients with wound infection

A total of 141 patients were included in the analysis. *Pseudomonas aeruginosa*–associated diabetic wound infections (PsA DWIs) were identified in 63 patients (44.68%), whereas non-PsA DWIs were detected in 78 patients (55.32%). The sociodemographic characteristics of patients with diabetic wound infections, with and without *P. aeruginosa*, are presented in Table 2.

The age distribution among patients with diabetes mellitus (DM) showed no statistically significant association with PsA infection. The highest proportion of cases was observed in the 50–59-year age group (31.91%), with a higher prevalence of non-PsA DWIs than PsA DWIs. The odds ratios (ORs) for PsA infection were 1.13 (95% CI: 0.44–2.91, p = 0.80), 1.17 (95% CI: 0.45–3.05, p = 0.75), and 1.31 (95% CI: 0.45–3.84, p = 0.63) for the 50–59, 60–69, and ≥70-year age groups, respectively, compared with the reference group (18–49 years). The mean age was also not significantly associated with PsA infection (OR = 1.00, 95% CI: 0.97–1.04, p = 0.78).

Similarly, sex distribution did not differ significantly between the two groups. Males had an OR of 0.74 (95% CI: 0.38–1.45, p = 0.38) compared with females. Females comprised 54.61% of the total sample, with comparable proportions in the non-PsA (51.95%) and PsA DWI (48.05%) groups.

Most participants resided in urban areas (78.01%), compared with 21.99% in rural areas. In both settings, the prevalence of PsA DWIs was lower than that of non-PsA DWIs. However, the difference was not statistically significant, with an OR of 1.21 for rural residence (95% CI: 0.55–2.69, p = 0.64).

Among all patients with diabetes mellitus (DM), obesity was the most prevalent BMI category (51.06%), with an equal distribution of PsA and non-PsA diabetic wound infections (DWIs). This was followed by the overweight group (31.91%) and the normal-weight group (17.02%). The highest proportion of PsA DWIs was observed in the normal-weight group (58.33%). Overweight individuals had significantly lower odds of PsA infection compared with the normal-weight group (OR = 0.29, 95% CI: 0.10–0.82, p = 0.019), reflecting a lower proportion of PsA DWIs (28.89%) relative to non-PsA DWIs (71.11%). In contrast, obesity was not significantly associated with PsA infection (OR = 0.71, 95% CI: 0.28–1.82, p = 0.48). Furthermore, the overall mean BMI was not significantly associated with PsA infection (OR = 1.03, 95% CI: 0.98–1.096, p = 0.26

Educational attainment varied among participants: 59.57% were illiterate, 21.48% had primary education, 13.48% had secondary or tertiary education, and 5.67% had higher education. Across all categories, the prevalence of PsA diabetic wound infections (DWIs) was less than or equal to that of non-PsA DWIs. Compared with the illiterate group (reference), the odds ratios (ORs) for PsA infection were 0.43 (95% CI: 0.18–1.04, p = 0.06) for primary education, 0.73 (95% CI: 0.27–1.99, p = 0.54) for

secondary/tertiary education, and 1.00 (95% CI: 0.24–4.27, p = 1.00) for higher education; none of these associations were statistically significant.

Approximately three-quarters (73.76%) of patients were not formally employed, including housewives (51.77%) and unemployed males (21.99%). The remaining participants were government employees (10.64%), daily laborers (9.93%), and pensioners (5.67%). A higher proportion of PsA DWIs was observed only among unemployed individuals (51.61%). However, no statistically significant association was found between occupational status and PsA infection in the unadjusted analysis, with daily laborers serving as the reference group. The ORs were 0.25 (p = 0.098) for government employees, 0.14 (p = 0.10) for pensioners, 0.97 (p = 0.96) for housewives, and 1.07 (p = 0.92) for unemployed individuals.

Regarding smoking status, non-smokers constituted the largest proportion of patients (65.96%), followed by former smokers (17.02%) and current smokers (17.02%). Non-smokers had a slightly higher prevalence of non-PsA DWIs compared with PsA DWIs (52.69% vs. 47.31%). In contrast, PsA DWIs were less frequent among former and current smokers compared with non-PsA DWIs. Neither former smoking (OR = 0.56, p = 0.22) nor current smoking (OR = 0.94, p = 0.90) showed a statistically significant association with PsA infection when compared with non-smokers. The number of patients with and without a family history of diabetes mellitus was nearly equal (74 vs. 67). In both groups, the prevalence of PsA DWIs was slightly lower than that of non-PsA DWIs; however, this difference was not statistically significant (OR = 1.40, p = 0.32).

In the multivariable analysis, some associations changed. The previously observed significant association for overweight individuals was no longer statistically significant (p = 0.08). In contrast, occupational categories such as government employees (p = 0.046) and pensioners (p = 0.047) became statistically significant, indicating an independent association with reduced odds of PsA infection.

Table 2: Sociodemographic characteristics of diabetes wound patients with *P. aeruginosa* and without *P. aeruginosa* infection

**Characteristic**	**Total No. (%) 141 (100)**	**Non-PsA DWIs No. (%) 78 (55.32)**	**PsA DWIs No. (%) 63 (44.68)**	**Unadjusted OR [95% CI, p-value]**	**Adjusted OR [95% CI, p-value]**
**Age Group (Year)**					
18-49	29 (20.57)	17 (58.62)	12 (41.38)	Ref	Ref
50-59	45 (31.91)	25 (55.56)	20 (44.44)	1.13 [0.44-2.91, p=0.80]	1.25 [0.41-3.81, p=0.69]
60-69	42 (29.79)	23 (54.76)	19 (45.24)	1.17 [0.45-3.05, p=0.75]	1.07 [0.33-3.48, p=0.91]
≥70	25 (17.73)	13 (52)	12 (48)	1.31 [0.45-3.84, p=0.63]	1.08 [0.29-4.08, p=0.91]
Mean ± SD	57.48 ± 10.66	57.26 ± 10.52	57.76 ± 10.91	1.00 [0.97-1.04, p=0.78]	------------------------
**Sex**					
Female	77 (54.61)	40 (51.95)	37 (48.05)	Ref	Ref
Male	64 (45.39)	38 (59.38)	26 (40.63)	0.74 [0.38- 1.45, p=0.38]	1.02 [0.06-17.7, p=0.99]
**Region**					

Urban	110 (78.01)	62 (56.36)	48 (43.64)	Ref	Ref
Rural	31 (21.99)	16 (51.61)	15 (48.39)	1.21 [0.55-2.69, p=0.64]	1.5 [0.60-3.78, p=0.39]
**BMI (kg/m^2)**					
Normal	24 (17.02)	10 (41.67)	14 (58.33)	Ref	Ref
Overweight	45 (31.91)	32 (71.11)	13 (28.89)	0.29 [0.10-0.82, p=0.019*]	0.35 [0.11-1.13, p=0.08]
Obesity	72 (51.06)	36 (50)	36 (50)	0.71 [0.28-1.82, p=0.48]	0.69 [0.23-2.09, p=0.51]
Mean ± SD	30.28 ± 5.82	29.80 ± 4.99	30.88 ± 6.69	1.03 [0.98-1.096, p=0.26]	------------------------
**Educational Level**					
Non-educational (illiterate)	84 (59.57)	42 (50)	42 (50)	Ref	Ref
Primary school	30 (21.48)	21 (70)	9 (30)	0.43 [0.18-1.04, p=0.06]	0.42 [0.14-1.23, p=0.11]
Secondary and Tertiary school	19 (13.48)	11 (57.89)	8 (42.11)	0.73 [0.27-1.99, p=0.54]	0.95 [0.26-3.5, p=0.94]
High educational (Institute or college)	8 (5.67)	4 (50)	4 (50)	1 [0.24-4.27, p=1]	4.84 [0.48-48.9, p=0.18]
**Occupational status**					
Daily Labor	14 (9.93)	7 (50)	7 (50)	Ref	Ref
Government Employee	15 (10.64)	12 (80)	3 (20)	0.25 [0.05-1.29, p=0.098]	0.12 [0.02-0.97, p=0.046*]
Pension	8 (5.67)	7 (87.5)	1 (12.5)	0.14 [0.01-1.49, p=0.10]	0.05 [0.003-0.96, p=0.047*]
Housewife	73 (51.77)	37 (50.68)	36 (49.32)	0.97 [0.31-3.05, p=0.96]	0.86 [0.04-16.9, p=0.92]
Unemployed	31 (21.99)	15 (48.39)	16 (51.61)	1.07 [0.30-3.77, p=0.92]	1.18 [0.24-5.77, p=0.84]
**Smoking**					
Non smoker	93 (65.96)	49 (52.69)	44 (47.31)	Ref	Ref
Former smoker	24 (17.02)	16 (66.67)	8 (33.33)	0.56 [0.22-1.43, p=0.22]	0.81 [0.23-2.85, p=0.74]
Current smoker	24 (17.02)	13 (54.17)	11 (45.83)	0.94 [0.38-2.32, p=0.90]	1.94 [0.49-7.63, p=0.35]
**Family History of DM**					
No	67 (47.52)	40 (59.7)	27 (40.3)	Ref	Ref
Yes	74 (52.48)	38 (51.35)	36 (48.65)	1.40 [0.72-2.74, p=0.32]	1.45 [0.66-3.22, p=0.36]
Note: **PsA** = *Pseudomonas aeruginosa,* **DWIs** = Diabetes Wound Infections, **OR**= odd ratio, * = statistically significant difference					

The clinical characteristics of diabetic patients with PsA DWIs and non-PsA DWIs

The clinical characteristics of PsA diabetic wound infections (DWIs) and non-PsA DWIs are presented in Table 3. Among the study population, 14 patients had glycated hemoglobin (HbA1c) levels between 4 and 6.9, while 127 patients had HbA1c levels ranging from 7 to 16. The overall mean HbA1c level was 9.23 ± 2.18.The mean HbA1c level was slightly higher in patients with non-PsA DWIs compared with those with PsA DWIs (9.25 ± 1.98 vs. 9.20 ± 2.43); however, this difference was not statistically significant (OR = 0.99, 95% CI: 0.85–1.15, p = 0.90).

The mean duration of diabetes was 13.86 ± 7.72 years in patients with PsA diabetic wound infections (DWIs) and 13.97 ± 8.99 years in those with non-PsA DWIs, with no statistically significant difference between the groups (OR = 0.99, p = 0.87). Similarly, the duration of wound infection (in months) did not differ significantly between the two groups (OR = 0.99, p = 0.16), although a higher mean duration was observed in non-PsA DWI cases compared with PsA DWI cases (13.13 ± 38.41 vs. 5.82 ± 11.34 months). In terms of wound location, infections in the leg region were the most common (78.72%),

comprising 49.55% of PsA DWIs and 50.45% of non-PsA DWIs. Leg infections were significantly associated with the outcome, with approximately 2.7-fold higher odds of PsA infection (OR = 2.70, 95% CI: 1.11–6.58, $p = 0.03$).

Regarding wound type, wet wounds were the most prevalent (60.99%) and showed higher, though not statistically significant, odds of PsA infection (OR = 1.78, 95% CI: 0.87–3.64, $p = 0.12$). This was followed by dry wounds (35.46%) and mixed wounds (3.55%), with ORs of 1.19 (95% CI: 0.18–7.77, $p = 0.86$). Among wet wounds, PsA and non-PsA DWIs were equally distributed (50% each). In contrast, PsA DWIs accounted for 39% of dry wounds and 40% of mixed wounds, whereas non-PsA DWIs represented 64% and 60% of these categories, respectively.

A higher prevalence of PsA DWIs was observed in ulcers with cellular necrosis and gangrene (54.55%) compared with non-PsA DWIs (45.45%). In other lesion types, PsA DWIs were equal to or less frequent than non-PsA DWIs (36.36% in abscesses, 33.33% in cellular necrosis, 50% in gangrene, 37.84% in ulcers, and 47.95% in ulcers with cellular necrosis). Although some categories showed higher odds ratios (ORs) than abscesses, none were statistically significant (all $p > 0.05$) and confidence intervals (CIs) were wide. Among 112 patients with comorbidities, hypertension was most common (56%). PsA DWIs were more frequent in patients with periodontal disease (51.61%) and hypertension (48.1%), but no significant associations were found (all $p > 0.05$; CIs included 1). Regarding treatment, 31.12% used diet plus oral hypoglycemic agents, while 23.4% used diet plus insulin. PsA DWIs were most common among patients receiving insulin plus oral agents (66.67%). However, treatment type was not significantly associated with PsA DWIs (all $p > 0.05$; CIs included 1). Most patients (90.07%) did not exercise. Non-PsA DWIs were more prevalent in both active and inactive groups, with no significant association ($p = 0.48$), although exercise showed a lower OR (0.66; 95% CI: 0.21–2.08). Wound dressing was used by 91.49% of patients. PsA DWIs were less frequent than non-PsA DWIs, with no statistically significant difference.

All independent variables were not statistically significant predictors of PsA in the multivariable logistic regression analysis, except for wound location (leg region), which was significant ($p = 0.02$).

Table 3: Clinical characteristics of diabetes wound patients with *P. aeruginosa* and without *P. aeruginosa* infection

**Characteristic**	**Total No. (%) 141 (100)**	**Non-PsA DWIs No. (%) 78 (55.32)**	**PsA DWIs No. (%) 63 (44.68)**	**Unadjusted OR [95% CI, p-value]**	**Adjusted OR [95% CI, p-value]**
**HBA1C (Mean ±SD)**	9.23 ± 2.18	9.25 ± 1.98	9.20 ± 2.43	0.99 [0.85-1.15, p=0.90]	0.88 [0.73-1.07, p=0.19]
**Duration of DM by year (Mean ±SD)**	13.92 ± 8.42	13.97 ± 8.99	13.86 ± 7.72	0.99 [0.96-1.04, p=0.87]	0.98 [0.92-1.03, p=0.40]
**History of ulcer by months (Wound infection)**	9.86 ± 29.29	13.13 ± 38.41	5.82 ± 11.34	0.99 [0.97-1.01, p=0.16]	0.98 [0.95-1.01, p=0.23]
**Location of wound infection (skin ulcer)**					
Non-Leg region	30 (21.28)	22 (73.33)	8 (26.67)	Ref	Ref
Leg region (Leg and Foot)	111 (78.72)	56 (50.45)	55 (49.55)	2.7 [1.11-6.58, p=0.03[*]]	3.94 [1.24-12.5, p=0.02[*]]
**Type of Wound infection**					
Dry	50 (35.46)	32 (64)	18 (36)	Ref	Ref
Wet	86 (60.99)	43 (50)	43 (50)	1.78 [0.87-3.64, p=0.12]	2.13 [0.90-5.22, p=0.09]
Both	5 (3.55)	3 (60)	2 (40)	1.19 [0.18-7.77, p=0.86]	0.63 [0.07-5.68, p=0.68]
**Degree of Wound infection (Lesion Severity) (Clinical presentations)**					
Abscess	11 (7.8)	7 (63.64)	4 (36.36)	Ref	Ref
Cellular death	3 (2.13)	2 (66.67)	1 (33.33)	0.88 [0.06-12.97, p=0.92]	0.59 [0.03-13.3, p=0.75]
Gangrene	6 (4.26)	3 (50)	3 (50)	1.75 [0.23-13.2, p=0.59]	1.53 [0.14-17.1, p=0.71]
Ulcer	37 (26.24)	23 (62.16)	14 (37.84)	1.07 [0.26-4.31, p=0.93]	1.93 [0.32-11.73, p=0.48]
Ulcer with Cellular Death	73 (51.77)	38 (52.05)	35 (47.95)	1.61 [0.43-5.98, p=0.48]	2.02 [0.36-11.52, p=0.43]
Ulcer with Cellular death and Gangrene	11 (7.8)	5 (45.45)	6 (54.55)	2.10 [0.38-11.6, p=0.40]	2.52 [0.26-24.01, p=0.42]
**Comorbid condition**					
Hypertension	79 (56)	41 (51.90)	38 (48.10)	1.35 [0.57-3.21, p=0.5]	1.69 [0.62-4.59, p=0.30]
Cardiac disease	43 (30.5)	26 (60.47)	17 (39.53)	0.74 [0.33-1.67, p=0.46]	0.58 [0.22-1.5, p=0.26]
Renal disease	33 (23.4)	20 (60.61)	13 (39.39)	0.74 [0.29-1.91, p=0.54]	0.55 [0.18-1.68, p=0.30]
Eye problem	70 (49.6)	40 (57.14)	30 (42.86)	0.73 [0.30-1.79, p=0.5]	0.72 [0.26-1.99, p=0.53]
Periodontal (gum) disease	31 (22)	15 (48.39)	16 (51.61)	1.61 [0.66-3.95, p=0.3]	1.84 [0.65-5.2, p=0.25]
Other infection	32 (22.7)	21 (65.63)	11 (34.38)	0.5 [0.20-1.2, p=0.12]	0.67 [0.24-1.91, p=0.46]
No other comorbid conditions	29 (20.57)	17 (58.62)	12 (41.38)	0.7 [0.19-2.3, p=0.52]	0.69 [0.17-2.81, p=0.61]

**Types of Diabetic treatment**					
Diets Only	5 (3.55)	4 (80)	1 (20)	Ref	Ref
Insulin Only	4 (2.84)	2 (50)	2 (50)	4.0 [0.21-75.7, p=0.36]	1.76 [0.06-53.75, p=0.75]
Oral Hypoglycemic Tablets Only	19 (13.48)	10 (52.63)	9 (47.37)	3.60 [0.34-38.5, p=0.29]	1.19 [0.09-16.35, p=0.9]
Diets and Insulin	33 (23.4)	20 (60.61)	13 (39.39)	2.60 [0.26-25.9, p=0.42]	1.15 [0.09-14.92, p=0.92]
Diets and Oral Hypoglycemic Tablets	44 (31.21)	22 (50)	22 (50)	4.0 [0.41-38.7, p=0.23]	1.73 [0.14-20.9, p=0.67]
Insulin and Oral Hypoglycemic Tablets	6 (4.26)	2 (33.33)	4 (66.67)	8.0 [0.50-127.9, p=0.14]	2.69 [0.13-56.28, p=0.52]
Diet, Insulin and Hypoglycemic Tablets	25 (17.73)	14 (56)	11 (44)	3.14 [0.31-32.3, p=0.34]	1.29 [0.09-17.54, p=0.85]
No treatment	5 (3.55)	4 (80)	1 (20)	1.0 [0.05-22.2, p=1.0]	0.13 [0.003-5.63, p=0.30]
**Exercise Status**					
No	127 (90.07)	69 (54.33)	58 (45.67)	Ref	Ref
Yes	14 (9.93)	9 (64.29)	5 (35.71)	0.66 [0.21-2.08, p=0.48]	0.50 [0.12-2.08, p=0.34]
**Wound Dressing Applied**					
No	12 (8.51)	7 (58.33)	5 (41.67)	Ref	Ref
Yes	129 (91.49)	71 (55.04)	58 (44.96)	1.14 [0.35-3.79, p=0.83]	0.46 [0.09-2.38, p=0.35]

Note: **PsA** = *Pseudomonas aeruginosa,* **DWIs** = Diabetes Wound Infections, **OR**= odd ratio * = statistically significant difference

Antibiotics susceptibility profile

Table 4 summarizes the antimicrobial susceptibility profile of *P. aeruginosa* isolates. High resistance rates were observed for aztreonam (89%) and ceftazidime (84%), followed by cefepime (81%) and tobramycin (86%). Moderate resistance was noted for amikacin (75%), ciprofloxacin (79%), and levofloxacin (79%), while lower resistance rates were observed for gentamicin (62%) and norfloxacin (56%).

Among carbapenems, resistance to imipenem and meropenem was 52% and 41%, respectively. Notably, colistin demonstrated the lowest resistance rate (37%) and the highest sensitivity (63%) among all tested antibiotics. Overall, *P. aeruginosa* isolates exhibited high levels of multidrug resistance, with relatively better susceptibility to colistin and carbapenems compared to other antibiotic classes.

Table 4: Antibiotics susceptibility profile among *P. aeruginosa* bacteria

Category	**Antibiotics /µg**	**Resistance No. (%)**	**Sensitive No. (%)**
Monobactam	Aztreonam (ATM 30)	56 (89)	7 (11)
Carbapenem	Imipenem (IPM 10)	33 (52)	30 (48)
	Meropenem (MEM 10)	26 (41)	37 (59)
3rd generation Cephalosporins	Ceftazidime (CAZ 30)	53 (84)	10 (16)
4th generation Cephalosporins	Cefepime (FEP 10)	51 (81)	12 (19)
Aminoglycosides	Amikacin (AK 10)	47 (75)	16 (25)
	Gentamicin (CN 10)	39 (62)	24 (38)

	Tobramycin (TOB 10)	54 (86)	9 (14)
Fluoroquinolones	Ciprofloxacin (CIP 10)	50 (79)	13 (21)
	Norfloxacin (NOR 30)	35 (56)	28 (44)
	levofloxacin (LEV 5)	50 (79)	13 (21)
Polypeptides	Colistin (CT 10)	23 (37)	40 (63)

Molecular identification

All *Pseudomonas aeruginosa* isolates were confirmed by PCR amplification of the 16S rDNA gene, producing a single band of 956 base pairs (Fig 1).

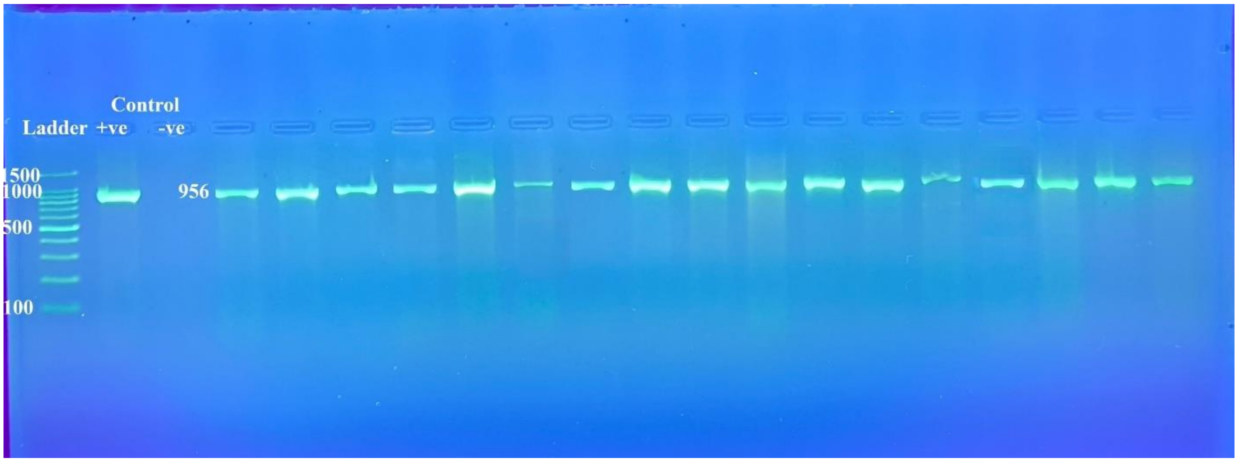

Fig 1: The amplification of PCR for species identification of P. aeruginosa using 16S rDNA primer. *The left lane shows the DNA ladder (100–1500 bp). The positive control (+ve) displays a clear band at 956 bp, while the negative control (−ve) shows no amplification. All tested isolates exhibit a distinct single band at approximately 956 bp, confirming the successful amplification of the target gene and molecular identification of* P. aeruginosa.

Discussion

Patients with diabetes mellitus represent a major risk group for skin wound infections, which may be caused by a variety of microorganisms, including *P. aeruginosa*. Previous studies on *P. aeruginosa* in diabetic patients have primarily focused on diabetic foot infections (DFIs). In contrast, the present study investigated P. *aeruginosa*–associated wound infections across all anatomical sites, reporting a prevalence of 63 cases (44.68%) among 141 patients. The identification of *P.s aeruginosa* in this study was confirmed using conventional biochemical tests, the Vitek®2 system, and 16S rDNA PCR amplification. The use of PCR provided a high level of specificity and accuracy in bacterial identification, thereby strengthening the reliability of the laboratory findings. This molecular confirmation reduces the possibility of misidentification that may occur with conventional methods alone and ensures more precise detection of *P. aeruginosa* in diabetic wound infections. Therefore, the combination of phenotypic and molecular techniques improves the validity of the reported prevalence and supports the robustness of the study results. This finding is consistent with a study conducted in Duhok city, in which all bacterial isolates were similarly confirmed using the 16S rDNA gene [23].

The prevalence of *P. aeruginosa* among DFU observed in this study is comparable to reports from Najaf, Iraq (34.4%) [9], Iran (35%) [24], and various regions of India, including North (23.7%), South (29.8%), North-East (11.89%), East (11.7%) and West India (27%) [25]. Similarly, a study conducted in Erbil, Iraq, reported that 53 of 150 DFU samples (35.33%) were positive with *P. aeruginosa* [11]. In the present study, PsA DWIs were less frequent than non-PsA DWIs, which contrasts with findings from a study in Anhui Province, China, where *P. aeruginosa* accounted for only 9% of infections compared to 91% for non-*P. aeruginosa* pathogens [26]. These variations may be attributed to differences in causative microorganisms, geographic location, and the severity and types of infections [27].

The current study found a higher prevalence rate of DM among females compared to males. Additionally, a higher rate of PsA DWIs was observed in the 50-59 age groups. A comparable study conducted in Kermanshah Province, Iran, reported a female prevalence of 57.4% and a male prevalence of 42.6% [1]. In contrast, a previous study in Najaf City, Iraq, found a lower proportion of females (44.3%) compared to males (55.7%) among 282 DM patients with foot wound infections [9]. Furthermore, another study in Iraq reported a male to female ratio of 2.5:1, while demonstrating a similar age distribution, with the highest incidence of DFI occurring in the 51-60 age groups [28]. The higher proportion of cases among females in the present study may be attributed to cultural variations influencing access to healthcare services [1].

The present study indicated that individuals residing in urban areas are more likely to develop wound infection compared to those living in rural areas. This study is consistent with a study conducted in Ethiopia, by Molla et al (2023), which reported a higher prevalence of DFI in urban population (62.4%) compared to rural (37.6%) [29]. Similarly, a study carried out in Erbil City, Iraq, demonstrated that

infection rates were higher in urban areas (54%) than in rural areas (46%) [11]. These variations may be attributed to differences in study populations and methodologies. Furthermore, several indicators used to assess socioeconomic status at the neighborhood level may provide insight into the environmental and social factors influencing the risk of infection among residents [30].

Conversely, obese individual exhibited the highest rates of infection compared to those who were overweight or normal weight. However, the prevalence rate of PsA DWIs was highest among individuals with normal weight, followed by overweight and obese groups. This finding is consistent with a study conducted in Iraq, which reported a higher incidence of diabetic foot infections (DFIs) among obese participants [5]. Additionally, a study from the United States found that the mean body mass index (BMI) among DFI patients with and without *P. aeruginosa* infection was 33.6% and 30.6%, respectively [31]. Over recent decades, obesity rates have increased dramatically, leading the World Health Organization to classify as a global epidemic and a major threat to public health [15]. Obesity is associated with several comorbid conditions, such as atherosclerosis, which may contribute to the development of diabetic foot ulcers (DFUs) and subsequent infections [5].

Educational level varied among participants, with illiterate individuals showing a higher likelihood of infection compared to other groups. In all categories, the prevalence of *P. aeruginosa* diabetic wound infections (DWIs) was lower than or equal to that of non-*P. aeruginosa* DWIs. To date, no studies have specifically examined the relationship between educational level and *P. aeruginosa* DWIs. Consistent with the present findings, a study conducted in Colombia reported that the majority of individuals with DM had not completed primary education [32]. Similarly, a study from Kirkuk City, Iraq, reported an illiteracy rate of 24%, compared to only 4% among those with higher education [4]. In contrast, several other studies have reported conflicting results, indicating higher rates of diabetic foot infections (DFIs) among individuals with higher educational attainment, including those with high school and university education [5, 33] while lower rates were observed among illiterate patients [29].

Moreover, evidence suggests that individuals with lower levels of education are more likely to develop DM and experience its complications [30]. These findings indicated that the level of education is important in taking care of metabolic diseases and the promotion of health-protective behaviors [4]. Patients with limited education may have reduced awareness regarding glycemic control and proper wound care practices, increasing their susceptibility to infection.

This study is partly consistent with findings from Iraq, where employment status was distributed as follows: housewives (37.6%), employees (12.4%), daily laborers (22.8%), and pensioners (27%) [5]. Similarly, a study from Ethiopia reported housewives (19%), government employees (26.8%), daily laborers (5.3%), pensioners (5%), unemployed (3.8%), and others (farmers and merchants) [29]. In our study, the highest rates of PsA DWIs were observed among non-working, non-occupational, and uneducated patients of both sexes.

Smoking patterns in our study were similar to a previous report, in which most patients were non-smokers (74.9%), followed by smokers (1.6%) and ex-smokers (9.5%) [5]. Another study reported a higher rate of PsA in foot infections compared with non-PsA cases (37% vs. 19%) [8]. In our study, no significant association was found between smoking status and PsA DWIs, although non-smokers had higher rates than current and former smokers.

The distribution of family history of diabetes in our study differs from findings in Iraq, where rates among DFI patients were negative (47%), positive (36%), and unknown (16.2%) [5]. Similarly, other studies reported negative rates of 62.2% and 72.7%, and positive rates of 37.8% and 27.3%, respectively [29,34]. Evidence consistently shows that individuals with a family history of diabetes are at higher risk of developing the disease, likely due to genetic factors [34,35].

The mean HbA1c in our study was comparable to previous reports (8.3 ± 2.2 [24], 8.94 ± 2.1 [36], and 8.61 ± 2.24 [37]), although one study reported a higher mean (10.26 ± 2.0) [5]. Similarly, a U.S. study reported a mean HbA1c of 8.3% [8]. HbA1c was higher in patients with PsA DFI than in non-PsA DFI (8.5% vs. 7.3%). Chronic hyperglycemia impairs wound healing, increases wound size, and is associated with complications and mortality [38,39]. Therefore, maintaining optimal HbA1c levels is essential for preventing diabetic foot complications.

Several studies have reported the mean ± SD of diabetes duration, including 14.5 ± 4.5 years [24], 13.6 ± 8.5 years [36], and 13.54 ± 6.98 years [5]. One study reported that 40.3% of patients had a duration ≤10 years, while 59.7% had >10 years [33], and another reported 11.1% [37]. The mean ulcer duration among DFU patients has been reported as 14.5 ± 4.5 months [24] and 1.8 months [37]. In contrast to studies focusing solely on foot infections, our study included multiple anatomical sites. Toe infections were most common (44%), including 30% PsA and 45% non-PsA DFI. The lateral plantar region accounted for 29% (22% PsA, 30% non-PsA), the heel for 16% (19% PsA, 16% non-PsA), and other sites for 14% (37% PsA, 11% non-PsA). Unspecified sites comprised 9% (4% PsA, 9% non-PsA) [8]. A significant association was observed between wound location and DM ($p = 0.001$), except for foot-related sites.

Based on infection severity, DFIs in our study were mainly ulcers (70%), followed by abscesses (21%) and combined ulcer–abscess lesions (10%). PsA DFIs were more frequent in ulcers (82%) than in non-PsA cases (68%), whereas abscesses and mixed lesions were more common in non-PsA DFIs [8]. Other studies reported varying distributions, including ulcers (44%), cellulitis (20%), abscesses (32%), gangrene (24%), and neuropathic ulcers (4%) [7], while another found ulcers in 50.2% of cases [37]. Overall, wound infection is a common complication of diabetes, often resulting from ulceration and inflammation, and may progress to gangrene and lower limb amputation, contributing to significant morbidity, hospitalization, and mortality [7].

Several studies have examined DFU and associated complications. In one study of 54 DFU cases, hypertension, retinopathy, nephropathy, neuropathy, and osteomyelitis were reported in 11, 8, 11, 24, and 10 patients, respectively [24]. Another study (2023) found that 62.2% of patients had

comorbidities, most commonly hypertension (30.8%), followed by combinations of cardiovascular, renal, and other chronic diseases, while 37.8% had none [29]. Similarly, another report showed hypertension (43.2%) as the most frequent comorbidity, followed by hypercholesterolemia (10.7%). Complications were present in 16% of cases, including nephropathy (1.9%), retinopathy (5.8%), neuropathy (4.9%), and other conditions, while 84% had no complications [33]. Ultimately, poor blood supply, infection, and neuropathy may progress to tissue necrosis, osteomyelitis, gangrene, and amputation [7,39]. The presence of multiple (≥3) complications in DM patients with wound infection is an indicator of increased amputation risk. Therefore, improved education, early detection, and timely management are essential to reduce major amputations [26].

Regarding treatment methods, one study reported that among DM patients, 26.6% used oral hypoglycemic agents, 43.6% insulin, 29.4% both insulin and oral agents, 0.3% other treatments, and 3.3% received no treatment [37]. Another study reported diet alone (10.7%), oral hypoglycemic agents (55.8%), insulin alone (8.3%), combined therapy (12.6%), and 12.6% with no treatment [29]. Similarly, oral hypoglycemic agents, insulin, and combination therapy were reported in 42.9%, 31.6%, and 25.6% of patients, respectively [29]. In addition, 43.2% of DM patients engaged in exercise, while 56.8% did not [33]. The risk of developing type 2 diabetes can be reduced through a healthy diet and regular exercise. Improved insulin sensitivity reduces hepatic glucose production, leading to lower HbA1c levels, which in turn decreases the risk of diabetes-related complications [40].

Most patients are managed with wound dressing and debridement [7], and about two-thirds of DM patients with wound infection receive antimicrobial dressings [37]. Selection of wound dressings should follow best practice recommendations (BPRs) and clinical practice guidelines (CPGs) to ensure appropriate moisture balance, optimal wound care, and support for surgical cleansing. Antimicrobial dressings may also promote faster healing compared with non-antimicrobial dressings [6].

The multivariate logistic regression results of the present study differ from previous reports, which identified various independent predictors of infection in DFU/DFI patients. One study reported that age >65 years (OR=1.242, 95% CI: 1.251–2.114, p=0.001) and HbA1c >7% (OR=2.155, 95% CI: 1.287–2.946, p=0.032) were the only significant factors associated with infection in DFUs [24]. In another study, smoking and insulin use were significant in unadjusted analysis (OR=2.6, 95% CI: 1.1–6, p=0.022 and OR=0.21, 95% CI: 0.08–5.2, p<0.001, respectively), but they lost significance or were excluded in the final regression model [8]. Furthermore, a study on DFU-related amputation found that infection site was associated with lower limb amputation in both univariate (OR=0.347, 95% CI: 0.790–0.926, p<0.001) and multivariate analyses (OR=0.816, 95% CI: 0.724–0.919, p<0.001) [26].

This study has several limitations. Its cross-sectional design limits the ability to establish causal or temporal relationships between risk factors and *P. aeruginosa* infection. In addition, being conducted in a single province with a relatively small sample size may restrict the generalizability of the findings. However, the study also has important strengths. Clinical wound samples were collected over a 12-month period, minimizing seasonal variation. Samples were obtained from multiple hospitals and private clinics in Zakho and Duhok, improving representativeness. Identification of P. aeruginosa was confirmed using biochemical methods and 16S rDNA PCR, ensuring high diagnostic accuracy. The study also included a broad range of demographic, clinical, and behavioral variables (e.g., age, BMI, smoking, wound characteristics, and glycemic control), allowing a comprehensive assessment of potential risk factors. Finally, antimicrobial susceptibility testing was performed using CLSI-standard disc diffusion methods, ensuring reliable and comparable resistance data.

Conclusion

Diabetic patients with wound infections represent complex cases that may lead to major complications. In the present study, most sociodemographic factors, including age, sex, residence, education level, smoking status, and family history of diabetes, were not significantly associated with *P. aeruginosa* diabetic wound infections in both unadjusted and adjusted analyses. BMI showed a significant association in the unadjusted model for overweight patients (p = 0.019), but this was not maintained after adjustment. In the multivariate analysis, only occupational status showed a significant association, where government employees (p = 0.046) and pensioners (p = 0.047) had a lower likelihood of *P. aeruginosa* infection compared with daily laborers. Overall, sociodemographic characteristics had limited independent predictive value for *P. aeruginosa* DWIs, except for occupational status. In this study, most clinical variables—including HbA1c level, duration of diabetes, ulcer history, comorbidities, treatment type, exercise status, and wound dressing—were not significantly associated with *P. aeruginosa* diabetic wound infections in both unadjusted and adjusted logistic regression analyses. The only significant independent predictor was wound location, where infections involving the leg region showed a significantly higher risk of *P. aeruginosa* infection (adjusted OR = 3.94, p = 0.02). Overall, these findings suggest that anatomical site of infection plays a key role in *P. aeruginosa* occurrence, while other clinical and behavioral factors showed no independent association. *P. aeruginosa* isolates showed high levels of multidrug resistance, with the highest resistance observed against aztreonam, ceftazidime, and tobramycin. Carbapenems showed moderate resistance, while colistin remained the most effective antibiotic with the highest sensitivity rate.

Therefore, early detection and prompt management of wounds are essential to prevent progression to deep tissue infection. Individuals with a family history of diabetes should be particularly aware of the risk of earlier onset, especially in the presence of high BMI. Improving patient outcomes also depends on strengthening the education level and training of healthcare professionals, particularly nurses, who play a key role in patient education on wound care and complication prevention. Strengthening infection control practices, promoting rational antibiotic use, and improving patient education on wound care are essential to reduce complications and antimicrobial resistance in diabetic patients. In addition, further clinical

trials are needed to guide the selection of appropriate antimicrobial agents for wound dressings to ensure optimal patient care

Acknowledgments
We would like to express sincere gratitude to all participants who took part in this study.

Conflict of Interest
The authors declared no conflict of interest regarding study content.

Financial Disclosure
The study did not receive any financial support

References

[1] Ghobadi A, Sarbarzeh PA, Jalilian M, Abdi A, Manouchehri S. Evaluation of Factors Affecting the Severity of Diabetic Foot Ulcer in Patients with Diabetes Referred to a Diabetes Centre in Kermanshah. Dovepress. 2020; 13: 693–703. http://doi.org/10.2147/DMSO.S242431

[2] Sivanmaliappan TS, Sevanan M. Antimicrobial Susceptibility Patterns of *Pseudomonas aeruginosa* from Diabetes Patients with Foot Ulcers. International Journal ofMicrobiology. 2011; 2011: 1–4. doi: 10.1155/2011/605195.

[3] Zhang J, Deng Y, Wan Y, Wang J, Xu J. Diabetes duration and types of diabetes treatment in data-driven clusters of patients with diabetes, Frontiers in Endocrinol. 2022; 10 (3389): 1–12. doi: 10.3389/fendo.2022.994836.

[4] Amen MR, Kumait AS, Jaafr AY. Risk Factors for Diabetic Foot Ulceration Among Patients: A Cross-Sectional Study in Kirkuk City , Iraq. Journal of Pioneering Medical Sciences.2025; 14 (07): 112–116, 2025.

[5] Mohammed AG, Al-waeli DK, Odhaib SA, Altemimi MT. Assessing the Features of Diabetic Foot Ulcers among Individuals with Type 2 Diabetes Mellitus in Thi Qar, Iraq. F1000 Research. 2025; 13 (539): 1–14.

[6] Ivory JD, Gethin G, Mcintosh C. Antimicrobial dressings for treating local infection in patients with diabetic foot ulcers. The Diabetic Foot Journal. 2018; 21 (3): 155–159.

[7] Kothari B. Clinical study and surgical management of diabetic foot: An observational study. International Journal of Surgery Science. 2022; 6 (1): 130–133.

[8] Veve MP, Mercuro NJ, Sangiovanni RJ, Santarossa M, Patel N. Prevalence and Predictors of *Pseudomonas aeruginosa* Among Hospitalized Patients With Diabetic Foot Infections. Open Forum Infectious Diseases. 2022; 9 (7): 1–8.

[9] Al-Khudhairy MK, Al-Shammari MMM. Prevalence of metallo-β-lactamase–producing *Pseudomonas aeruginosa* isolated from diabetic foot infections in Iraq. New Microbe and New Infect. 2000; 35 (100661): 1–6.

[10] Al Abbas LA, Khaddam W, Shreibati F. INVESTIGATING BACTERIA ISOLATED FROM DIABETIC FOOT ULCERS AND STUDYING THEIR SENSITIVITY TO ANTIBIOTICS –SYRIA. Bulletin of

Pharmaceutical Sciences Assiut University. 2022; 45 (1): 451–458.

[11] Khader KA, Raad AZ. Molecular detection of toxA and lasB virulence genes of *Pseudomonas aeruginosa* strains isolated from Diabetic Foot Ulcers in Erbil, Iraq. Journal of Applied and Natural Science. 2025; 17 (3): 1386–1392.

[12] Muhammed S, Aziz AD, Ahmed MJ. Microbial Identification And Antibiotic Resistance Patterns In Diabetic Foot Ulcers From Patients In Erbil, Kurdistan Region, Iraq. The Review of DIABETIC STUDIES. 2025; 21: 634–644.

[13] Qadir AN, Mahmoud BM, Mahwi TO, Mohammed D, Arif R, Mahmood SO. Prevalence of Microorganisms and Antibiotic Sensitivity Among Patients with Diabetic Foot Ulcer in Sulaimani City, Iraq. Hospital Practices and Research. 2020; 5 (2): 56–63. doi: 10.34172/hpr.2020.11.

[14] Horcajada JP, Montero M, Oliver A, Sorlí L, Luque S, Gómez-Zorrilla S, et al. Epidemiology and Treatment of Multidrug-Resistant and Extensively Drug-Resistant. Clinical Microbiology Reviews. 2019; 32 (4): 1–52.

[15] Kolimechkov S, Petrov L. The Body Mass Index : A Systematic Review. Journal of Exercise Physiology and Health. 2020; 3 (2): 21–27.

[16] Apridamayanti P, Meilinasary KA, Sari R. Antibiotic Sensitivity in *Pseudomonas aeruginosa* of Diabetic Patient ' s Foot Ulcer. Pharm Sci Res. 2016; 3 (2): 80–87.

[17] Callahan A, Liam P. A Clinical Review of Necrotic Skin Lesions as Presentations of Systemic Infection. Preprints.org. 2025: 0–17. doi: 10.20944/preprints202507.1668.v1.

[18] Alexander SK, Strete D. Microbiology: A Photographic Atlas For The Laboratory. USA: Daryl F, 2001.

[19] Cheesbrough M. District Laboratory Practice in Tropical Countries. Second edition. New York, USA: Cambridge University Press, 2006.

[20] Spilker T, Coenye T, Vandamme P, Lipuma JJ. PCR-Based Assay for Differentiation of *Pseudomonas aeruginosa* from Other Pseudomonas Species Recovered from Cystic Fibrosis Patients. JOURNAL OF CLINICAL MICROBIOLOGY. 2004; 42 (5): 2074–2079. doi: 10.1128/JCM.42.5.2074.

[21] Polse RF. Bacteriological and Molecular Identification of *Pseudomonas aeruginosa* fron Burns and Wound Infection in Duhok Province/Kurdistan Region/Iraq. Doctoral dissertation, University of Zakho, 2024.

[22] Ausubel FM, Brent R, Kingston RE, Moore DD, Seidman JG, Smith JA, et al. Current Protocols in Molecular Biology. Ringbou. Wiley S, 2003.

[23] Polse RF, Khalid HM, Mero WMS. Distribution of blaOXA-10, blaPER-1, and blaSHV genes in ESBL-producing *Pseudomonas aeruginosa* strains isolated from burn patients. Scientific Reports. 2023; 13 (18402): 1–8. doi: 10.1038/s41598-023-45417-4.

[24] Samani S, Parsa H. Microbiological Features and Risk Factors in Patients With Diabetic Foot Ulcers. Index Wounds. 2015; 27 (11): 308–312.

[25] Chatterjee D, Sivashanmugam K. Insights on MDR Mechanism of *Pseudomonas aeruginosa* with Emphasis on Diabetic Foot Ulcer in the Indian Subcontinent. Journal of Pure and Applied Microbiolgy. 2024; 18 (2): 837–852. doi: 10.22207/JPAM.18.2.08.

[26] Yao Y, Chen L, Qian Y. Age Characteristics of Patients With Type 2 Diabetic Foot Ulcers and Predictive Risk Factors for Lower Limb Amputation: A Population-Based Retrospective Study. Journal of Diabetes Research. 2024; 2024 (1): 1–8. doi: 10.1155/jdr/2380337.

[27] Garousi M, Monazamitabar S, Mirazi H, Farrokhi Z, Khaledi A, ShakerimoghaddamA. Epidemiology of *Pseudomonas aeruginosa* in diabetic foot infections: a global systematic review and meta-analysi. Germs. 2-23; 13 (4): 362–372.

[28] Mahmoud HM, Abdul S, Abid A, Qadir D. Prevalence of bacterial types in Wagner grade three of diabetic foot ulcer. Iraqi Journal of Science. 2014; 55 (3): 1232–1235.

[29] Molla IB, Berhie MA, Debele KA, Germossa GN, Hailu FB. Persons with Diabetes' Perceptions of Family Burden and Associated Factors. Journal of Diabetes Research. 2023; 2023: 1–8.

[30] Schmidt BM, Huang Y, Banerjee M, Hayek SS, Pop-busui R. Residential Address Amplifies Health Disparities and Risk of Infection in Individuals With Diabetic Foot Ulcers Residential Address Amplifies Health Disparities and Risk of Infection in Individuals With Diabetic Foot Ulcers. Diabetes Care. 2024; 47: 508–515.

[31] J. Lau, J. Huang, and Z. K. Escobar, "Antipseudomonal Antibiotics in Diabetic Foot Infections : A Practical Perspective From a Community Hospital," Open Forum Infect. Dis., vol. 11, no. 6, pp. 1–4, 2024, doi: 10.1093/ofid/ofae258.

[32] Ramirez-Perdomo C, Perdomo-Romero A, Rodríguez-Vélez M. Knowledge and practices for the prevention of the diabetic foot. Rev Gaúcha Enferm. 2019; 40 (e20180161): 1–7.

[33] Ghanem SS, Abdulkreem EM, Alfurayh MS, Ahmed AA, Rikabi HA. The Role of HbA1c in the Follow-Up and Control of Diabetes Mellitus. Cureus. 2025; 17 (2): 1–7. doi: 10.7759/cureus.78357.

[34] Erasmus RT, Blanco EB, Okesina AB, Arana JM, Gqweta Z, Matsha T. Importance of family history in type 2 black South African diabetic patients. Postgraduate Medical Journal. 2001;77:323–325. doi: 10.1136/pmj.77.907.323.

[35] Peng H, Wang M, Guo H, Hou T, Li Y, Zhang H, etal. Parental Transmission of Type 2 Diabetes Risk in Offspring: A Prospective Family-Based Cohort Study in Northern China. Nutrients. 2025; 17 (1361): 1–12.

[36] Alharithy MK, Alobaylan MM, Alsugai ZO, Alswat KA. IMPACT OF FAMILY HISTORY OF DIABETES ON DIABETES CONTROL AND COMPLICATIONS. ENDOCRINE PRACTICE. 2018; 24 (9): 773–779. doi: 10.4158/EP-2018-0071.

[37] Ndosi M, Wright-Hughes A, Brown S, Backhouse M, Lipsky BA, Bhogal M, et al. Prognosis of the infected diabetic foot ulcer: a 12-month prospective observational study. Diabetic Medicine. 2018; 35: 78–88. doi: 10.1111/dme.13537.

[38] Fesseha BK, Abularrage CJ, Hines KF, Sherman R, Frost P, Langan S, et al. Association of

Hemoglobin A 1c and Wound Healing in Diabetic Foot Ulcers. Diabetes Care. 2018; 41: 1478–1485. doi: 10.2337/dc17-1683.

[39] Dogan M, Onar LC, Aydin B, Gumustas SA. Is high level of hemoglobin A1C an indicator for extended period of antibiotherapy in diabetic foot ulcers? Infectious Diseases & Microbiology. 2019; 6 (1): 21–27. doi: 10.14744/nci.2018.25582.

[40] Amer BE, Abdelgalil MS, Hamad AA, Abdelsayed K, Elaraby A, Abozaid AM, et al. Metformin plus lifestyle interventions versus lifestyle interventions alone for the delay or prevention of type 2 diabetes in individuals with prediabetes : a meta-analysis of randomized controlled trials. Diabetology & Metabolic Syndrome. 2024; 16 (273): 1–14.

Dear Editor,

We are pleased to submit the revised version of our manuscript entitled *"Prevalence and Associated Risk Factors of Pseudomonas aeruginosa Infection in Diabetic Wounds in Duhok Province, Iraq."*

We would like to thank the reviewers and editorial team for their valuable comments and constructive suggestions, which have significantly improved the quality of our manuscript. We have carefully addressed all comments, and all revisions and changes have been highlighted in red color in the revised manuscript for ease of review.

We hope that the revised version meets the journal's requirements and is suitable for publication.

Sincerely,

Dr Ibrahim Naqid

Reviewer #1:

1. The consent form that was supposed to be written was not part of the submission, even though it was mentioned in the manuscript.
   Thank you for your comment. We apologize for the oversight regarding the consent form. The written informed consent, which was mentioned in the manuscript, has now been uploaded to the submission system as required.

2. The English language needs substantial revision. Pseudomonas aeruginosa must consistently be typed in Italic font.
   Thank you for your comment. We have thoroughly revised the manuscript to improve the English language quality throughout the text. In addition, *Pseudomonas aeruginosa* has been consistently formatted in italic font in all instances, as required.

3. The classification of woutnd infection severity in the methodology section requires further clarification. What criteria were used to classify the severity, including the count and percentage?
   Thank you for your comment. We have clarified the classification of wound infection severity in the methodology section. The severity was defined based on the degree of tissue involvement and clinical presentation, including ulcers (necrosis with erythema, pain, and discharge), abscess formation (pus collection), cellular necrosis (tissue death due to hypoxia or vascular occlusion), and gangrene (advanced necrosis with or without systemic symptoms). The count and percentage of each category have been clearly reported in Table 3.

4. Table 3 presents the findings, however, the column headings do not describe the significance of the numbers presented, specifically which number indicates the sample size and which represents the % ?
   Thank you for your comment. We have revised Table 3 to improve clarity. The column headings have been updated to clearly distinguish the sample size (n) and percentage (%), ensuring that each value is correctly identified and easily interpreted.

5. What is the significance of (*) in the P-value column of table 3?
   Thank you for your comment. The asterisk (*) in the P-value column of Table 3 indicates a statistically significant result ($p < 0.05$). This clarification has now been added in the footnote of Table 3 as “ =* statistically significant difference” to ensure clear interpretation.

6. What is the significance of the PCR amplification in this study? No discussion of the findings in the context of the manuscript
   Thank you for your comment. The required changes added in the first paragraph of discussion. PCR amplification of the 16S rDNA gene was used in this study to confirm the identification of *Pseudomonas aeruginosa* with high specificity and accuracy. This molecular method strengthens the reliability of the conventional biochemical and Vitek®2 identification results by reducing the possibility of misidentification. Therefore, PCR confirmation supports the validity of the reported prevalence and ensures accurate detection of *P. aeruginosa* in diabetic wound infections.

7. The discussion should not reiterate the results; it should analyze and interret the findings in relation to the existing published data in the literature.
   Thank you for your comment. We have revised the Discussion section by removing repeated results and ensuring that it focuses on interpretation and comparison of findings with relevant published literature. All redundant result descriptions have been deleted and the section has been restructured to improve analytical depth and alignment with standard discussion format.

8. The conclusion should recommend measures to be taken to address the problems raised in the research questions.
   Thanks. The required changes have been added in the conclusion section. *Pseudomonas aeruginosa* is a significant pathogen in diabetic wound infections with high multidrug resistance, while most sociodemographic and clinical factors showed no strong independent association. The findings highlight the importance of early detection and proper management of diabetic wounds, particularly in high-risk anatomical sites such as the leg region. Strengthening infection control practices, promoting rational antibiotic use, and improving patient education on wound care are essential to reduce complications and antimicrobial resistance in diabetic patients.

9. What are the limitations associated with the current study?
   Thank you for your comment. We have addressed this point by clearly stating the study limitations in the Discussion section. These include the cross-sectional design, which limits causal inference, the single-province setting, and the relatively small sample size, which may affect generalizability.

9. **Reviewer #2: I. Summary of the Research**
This manuscript investigates the prevalence, associated risk factors, and antimicrobial susceptibility patterns of Pseudomonas aeruginosa in diabetic wound infections in Duhok Province, Iraq. A notable strength of this work is its departure from the standard focus on diabetic foot ulcers (DFUs) to include various anatomical sites, which provides a more comprehensive understanding of the pathogen's impact. The study utilizes a multi-pronged identification approach, including molecular confirmation via 16S rDNA PCR, which enhances the analytical validity of the findings.

Thank you for your thoughtful and constructive evaluation of our manuscript. We appreciate your recognition of the study's broader scope beyond diabetic foot ulcers, as well as the inclusion of multiple anatomical sites to provide a more comprehensive understanding of *Pseudomonas aeruginosa* infections in diabetic wounds.
Your positive feedback is highly valued and encourages us to further refine and strengthen our work.

However, the manuscript contains a critical temporal error and several major methodological and statistical deficiencies that must be addressed to ensure scientific integrity and reproducibility.

II. Major Revisions (Critical)

1. Fundamental Temporal Inconsistency
Issue: The study period is reported as being in the future ("July 2024 to July 2025") in the Abstract (Line 35) and the Methods (Line 112). This discrepancy suggests the results are hypothetical or simulated, which severely undermines the credibility of the entire paper.
Recommendation: Immediately correct the dates to reflect the actual, past timeframe of data collection. This is a non-negotiable requirement for truthful reporting.
Thank you for this important comment. We apologize for the error in reporting the study period. The dates have been corrected to reflect the actual data collection timeframe, and all inconsistencies in the Abstract and Methods sections have been revised accordingly to ensure accurate and consistent reporting throughout the manuscript.

2. Statistical Rigor and Multivariable Analysis
Issue: The statistical analysis is underdeveloped, appearing only in Lines 187. The authors list t-tests and Chi-square tests but omit any multivariable analysis (e.g., binary logistic regression). Without controlling for confounders, the claims regarding "associated risk factors" are not robustly supported.
Recommendation: Conduct a multivariable analysis to identify independent risk factors and report adjusted odds ratios (AORs) with 95% confidence intervals (CIs).
Thank you for your comment. We have performed a full reanalysis using appropriate multivariable logistic regression to control for potential confounders. The results have been updated throughout the manuscript, and adjusted odds ratios (AORs) with 95% confidence intervals (95% CIs) are now reported. In addition, the Discussion section has been revised accordingly to reflect the findings of the adjusted analysis and to ensure accurate interpretation of independent risk factors.

3. Sample Size Justification

Issue: The manuscript mentions the sample size of 141 in Line 113, but provides no justification for this number. Without a power analysis, it is unclear if the study was adequately powered to detect significant associations, such as those for BMI and wound site.

Recommendation: Provide a detailed sample size calculation or a retrospective power analysis in the Methods section.

Thank you for your valuable comment. This has been addressed and the sample size justification has been added in the Methods section. The sample size of 141 was determined based on all eligible diabetic wound infection cases admitted during the study period in selected hospitals. Therefore, a census sampling approach was used rather than a calculated sample size.

4. Data Availability and Transparency

Issue: The statement that “All relevant data are within the manuscript and its Supporting Information files” is misleading because there are no Supporting Information files included in the submission.

Recommendation: Upload the anonymized dataset as per the journal’s format (e.g. as Supplementary Files).

Thank you for your comment. We apologize for the confusion. The data availability statement has been revised accordingly, and the anonymized dataset will be uploaded as Supplementary Files in the required journal format.

III. Specific Comments & Technical Points

Results and Logical Consistency

- BMI Misinterpretation: The text in Line 209 (Results) and Line 325 (Discussion) incorrectly implies that obese individuals had the highest "rate" of P. aeruginosa infection. However, Table 2 (Line 233) clearly shows Normal weight individuals had the highest rate (58.33%) within their group. Revise the narrative to match the data.

Thank you for your careful observation, and we apologize for the confusion caused by this inconsistency. Although obesity was the most prevalent BMI category among diabetic patients in the study population, the highest proportion of *Pseudomonas aeruginosa* diabetic wound infections (PsA DWIs) was observed in the normal-weight group (58.33%).

- Unsupported Conclusions: The conclusion in Line 352 states that the highest rates were among the "uneducated". This contradicts Line 217, which explicitly states the association with educational level was not statistically significant (P = 0.2933).

Thank you for your careful review, and we apologize for the confusion. We have revised the conclusion accordingly to ensure consistency and accuracy

- Clarity and Style

Redundancy: Remove the redundant phrasing ("widespread epidemic" repeated) in Line 64.
Thank you for your comment. The redundant phrasing (“widespread epidemic”) in Line 64 has been removed as suggested.

Standardization: Ensure "P. aeruginosa" is formatted consistently throughout.

Thank you for your comment. The formatting of *P. aeruginosa* has been standardized consistently throughout the manuscript.

Sentence Structure: Simplify the convoluted description of wound locations in Line 246 and treatment types in Line 265.

Thank you for your comment. The sentence structure describing wound locations and treatment types has been simplified for clarity

- Reproducibility Details:

Sampling: Explicitly state if consecutive, random, or convenience sampling was used in Line 112.

We have clarified the sampling method in the Methods section

CLSI version: Specify the exact version/year of the CLSI guidelines used in Line 149.

Thank you for your comment. The exact version/year of the Clinical and Laboratory Standards Institute (CLSI) guidelines

PCR Master Mix: Provide the manufacturer and catalog number for the master mix in Line 164.
Thank you for your comment. The manufacturer and catalog number of the PCR master mix have been added as requested
