# Supplementary material for: Prevalence and associated risk factors of Pseudomonas aeruginosa infection in Diabetic wounds in Duhok province, Iraq
Source: PLoS One. 2026 Jul 24;21(7):e0354256. doi: 10.1371/journal.pone.0354256 (PMC13399349; doi:10.1371/journal.pone.0354256)
Supplement: Attachment — Submitted filename: Reviewer comment 25.04.2026.docx [file pone.0354256.s004.docx]

Dear Editor,

We are pleased to submit the revised version of our manuscript entitled “Prevalence and Associated Risk Factors of Pseudomonas aeruginosa Infection in Diabetic Wounds in Duhok Province, Iraq.”

We would like to thank the reviewers and editorial team for their valuable comments and constructive suggestions, which have significantly improved the quality of our manuscript. We have carefully addressed all comments, and all revisions and changes have been highlighted in red color in the revised manuscript for ease of review.

We hope that the revised version meets the journal’s requirements and is suitable for publication.

Sincerely,

Dr Ibrahim Naqid

Reviewer #1:

1. The consent form that was supposed to be written was not part of the submission, even though it was mentioned in the manuscript.

Thank you for your comment. We apologize for the oversight regarding the consent form. The written informed consent, which was mentioned in the manuscript, has now been uploaded to the submission system as required.

2. The English language needs substantial revision. Pseudomonas aeruginosa must consistently be typed in Italic font.

Thank you for your comment. We have thoroughly revised the manuscript to improve the English language quality throughout the text. In addition, Pseudomonas aeruginosa has been consistently formatted in italic font in all instances, as required.

3. The classification of woutnd infection severity in the methodology section requires further clarification. What criteria were used to classify the severity, including the count and percentage?

Thank you for your comment. We have clarified the classification of wound infection severity in the methodology section. The severity was defined based on the degree of tissue involvement and clinical presentation, including ulcers (necrosis with erythema, pain, and discharge), abscess formation (pus collection), cellular necrosis (tissue death due to hypoxia or vascular occlusion), and gangrene (advanced necrosis with or without systemic symptoms). The count and percentage of each category have been clearly reported in Table 3.

4. Table 3 presents the findings, however, the column headings do not describe the significance of the numbers presented, specifically which number indicates the sample size and which represents the % ?

Thank you for your comment. We have revised Table 3 to improve clarity. The column headings have been updated to clearly distinguish the sample size (n) and percentage (%), ensuring that each value is correctly identified and easily interpreted.

5. What is the significance of (*) in the P-value column of table 3?

Thank you for your comment. The asterisk () in the P-value column of Table 3 indicates a statistically significant result (p < 0.05). This clarification has now been added in the footnote of Table 3 as “ = statistically significant difference” to ensure clear interpretation.

6. What is the significance of the PCR amplification in this study? No discussion of the findings in the context of the manuscript

Thank you for your comment. The required changes added in the first paragraph of discussion. PCR amplification of the 16S rDNA gene was used in this study to confirm the identification of Pseudomonas aeruginosa with high specificity and accuracy. This molecular method strengthens the reliability of the conventional biochemical and Vitek®2 identification results by reducing the possibility of misidentification. Therefore, PCR confirmation supports the validity of the reported prevalence and ensures accurate detection of P. aeruginosa in diabetic wound infections.

7. The discussion should not reiterate the results; it should analyze and interret the findings in relation to the existing published data in the literature.

Thank you for your comment. We have revised the Discussion section by removing repeated results and ensuring that it focuses on interpretation and comparison of findings with relevant published literature. All redundant result descriptions have been deleted and the section has been restructured to improve analytical depth and alignment with standard discussion format.

8. The conclusion should recommend measures to be taken to address the problems raised in the research questions.

Thanks. The required changes have been added in the conclusion section. Pseudomonas aeruginosa is a significant pathogen in diabetic wound infections with high multidrug resistance, while most sociodemographic and clinical factors showed no strong independent association. The findings highlight the importance of early detection and proper management of diabetic wounds, particularly in high-risk anatomical sites such as the leg region. Strengthening infection control practices, promoting rational antibiotic use, and improving patient education on wound care are essential to reduce complications and antimicrobial resistance in diabetic patients.

9. What are the limitations associated with the current study?

Thank you for your comment. We have addressed this point by clearly stating the study limitations in the Discussion section. These include the cross-sectional design, which limits causal inference, the single-province setting, and the relatively small sample size, which may affect generalizability.

9. **Reviewer #2: I. Summary of the Research**
   This manuscript investigates the prevalence, associated risk factors, and antimicrobial susceptibility patterns of Pseudomonas aeruginosa in diabetic wound infections in Duhok Province, Iraq. A notable strength of this work is its departure from the standard focus on diabetic foot ulcers (DFUs) to include various anatomical sites, which provides a more comprehensive understanding of the pathogen's impact. The study utilizes a multi-pronged identification approach, including molecular confirmation via 16S rDNA PCR, which enhances the analytical validity of the findings.

Thank you for your thoughtful and constructive evaluation of our manuscript. We appreciate your recognition of the study’s broader scope beyond diabetic foot ulcers, as well as the inclusion of multiple anatomical sites to provide a more comprehensive understanding of Pseudomonas aeruginosa infections in diabetic wounds.

Your positive feedback is highly valued and encourages us to further refine and strengthen our work.

However, the manuscript contains a critical temporal error and several major methodological and statistical deficiencies that must be addressed to ensure scientific integrity and reproducibility.
 **II. Major Revisions (Critical)**

1. Fundamental Temporal Inconsistency
   Issue: The study period is reported as being in the future (“July 2024 to July 2025”) in the Abstract (Line 35) and the Methods (Line 112). This discrepancy suggests the results are hypothetical or simulated, which severely undermines the credibility of the entire paper.
   Recommendation: Immediately correct the dates to reflect the actual, past timeframe of data collection. This is a non-negotiable requirement for truthful reporting.

Thank you for this important comment. We apologize for the error in reporting the study period. The dates have been corrected to reflect the actual data collection timeframe, and all inconsistencies in the Abstract and Methods sections have been revised accordingly to ensure accurate and consistent reporting throughout the manuscript.

2. Statistical Rigor and Multivariable Analysis
   Issue: The statistical analysis is underdeveloped, appearing only in Lines 187. The authors list t-tests and Chi-square tests but omit any multivariable analysis (e.g., binary logistic regression). Without controlling for confounders, the claims regarding "associated risk factors" are not robustly supported.
   Recommendation: Conduct a multivariable analysis to identify independent risk factors and report adjusted odds ratios (AORs) with 95% confidence intervals (CIs).

Thank you for your comment. We have performed a full reanalysis using appropriate multivariable logistic regression to control for potential confounders. The results have been updated throughout the manuscript, and adjusted odds ratios (AORs) with 95% confidence intervals (95% CIs) are now reported. In addition, the Discussion section has been revised accordingly to reflect the findings of the adjusted analysis and to ensure accurate interpretation of independent risk factors.

3. Sample Size Justification
   Issue: The manuscript mentions the sample size of 141 in Line 113, but provides no justification for this number. Without a power analysis, it is unclear if the study was adequately powered to detect significant associations, such as those for BMI and wound site.
   Recommendation: Provide a detailed sample size calculation or a retrospective power analysis in the Methods section.

Thank you for your valuable comment. This has been addressed and the sample size justification has been added in the Methods section. The sample size of 141 was determined based on all eligible diabetic wound infection cases admitted during the study period in selected hospitals. Therefore, a census sampling approach was used rather than a calculated sample size.

4. Data Availability and Transparency
   Issue: The statement that “All relevant data are within the manuscript and its Supporting Information files” is misleading because there are no Supporting Information files included in the submission.
   Recommendation: Upload the anonymized dataset as per the journal’s format (e.g. as Supplementary Files).

Thank you for your comment. We apologize for the confusion. The data availability statement has been revised accordingly, and the anonymized dataset will be uploaded as Supplementary Files in the required journal format.

**III. Specific Comments & Technical Points
Results and Logical Consistency**

- BMI Misinterpretation: The text in Line 209 (Results) and Line 325 (Discussion) incorrectly implies that obese individuals had the highest "rate" of P. aeruginosa infection. However, Table 2 (Line 233) clearly shows Normal weight individuals had the highest rate (58.33%) within their group. Revise the narrative to match the data.

Thank you for your careful observation, and we apologize for the confusion caused by this inconsistency. Although obesity was the most prevalent BMI category among diabetic patients in the study population, the highest proportion of Pseudomonas aeruginosa diabetic wound infections (PsA DWIs) was observed in the normal-weight group (58.33%).

- Unsupported Conclusions: The conclusion in Line 352 states that the highest rates were among the "uneducated". This contradicts Line 217, which explicitly states the association with educational level was not statistically significant (P = 0.2933).

Thank you for your careful review, and we apologize for the confusion. We have revised the conclusion accordingly to ensure consistency and accuracy

- Clarity and Style
Redundancy: Remove the redundant phrasing ("widespread epidemic" repeated) in Line 64. Thank you for your comment. The redundant phrasing (“widespread epidemic”) in Line 64 has been removed as suggested.

Standardization: Ensure "P. aeruginosa" is formatted consistently throughout.

Thank you for your comment. The formatting of P. aeruginosa has been standardized consistently throughout the manuscript.

Sentence Structure: Simplify the convoluted description of wound locations in Line 246 and treatment types in Line 265.

Thank you for your comment. The sentence structure describing wound locations and treatment types has been simplified for clarity

- Reproducibility Details:
Sampling: Explicitly state if consecutive, random, or convenience sampling was used in Line 112.

We have clarified the sampling method in the Methods section

CLSI version: Specify the exact version/year of the CLSI guidelines used in Line 149.

Thank you for your comment. The exact version/year of the Clinical and Laboratory Standards Institute (CLSI) guidelines

PCR Master Mix: Provide the manufacturer and catalog number for the master mix in Line 164.
Thank you for your comment. The manufacturer and catalog number of the PCR master mix have been added as requested
