# Supplementary material for: Prevalence and associated risk factors of Pseudomonas aeruginosa infection in Diabetic wounds in Duhok province, Iraq
Source: PLoS One. 2026 Jul 24;21(7):e0354256. doi: 10.1371/journal.pone.0354256 (PMC13399349; doi:10.1371/journal.pone.0354256)
Supplement: Peer review history

# Peer review: Decision Letter 0

**DOI:** 10.1371/journal.pone.0354256.r001

**Authors:** Ghadeer Suaifan

19 Mar 2026

PONE-D-25-68291

Prevalence and associated risk factors of Pseudomonas aeruginosa infection in Diabetic wounds in Duhok province, Iraq

PLOS One

Dear Dr. Naqid,

Thank you for submitting your manuscript to PLOS ONE. After careful consideration, we feel that it has merit but does not fully meet PLOS ONE’s publication criteria as it currently stands. Therefore, we invite you to submit a revised version of the manuscript that addresses the points raised during the review process.

Please submit your revised manuscript by May 03 2026 11:59PM. If you will need more time than this to complete your revisions, please reply to this message or contact the journal office at plosone@plos.org. When you're ready to submit your revision, log on to https://www.editorialmanager.com/pone/ and select the 'Submissions Needing Revision' folder to locate your manuscript file.

Please include the following items when submitting your revised manuscript:

- A letter that responds to each point raised by the academic editor and reviewer(s). You should upload this letter as a separate file labeled 'Response to Reviewers'.
- A marked-up copy of your manuscript that highlights changes made to the original version. You should upload this as a separate file labeled 'Revised Manuscript with Track Changes'.
- An unmarked version of your revised paper without tracked changes. You should upload this as a separate file labeled 'Manuscript'.

If you would like to make changes to your financial disclosure, please include your updated statement in your cover letter. Guidelines for resubmitting your figure files are available below the reviewer comments at the end of this letter.

If applicable, we recommend that you deposit your laboratory protocols in protocols.io to enhance the reproducibility of your results. Protocols.io assigns your protocol its own identifier (DOI) so that it can be cited independently in the future. For instructions see: https://journals.plos.org/plosone/s/submission-guidelines#loc-laboratory-protocols. Additionally, PLOS ONE offers an option for publishing peer-reviewed Lab Protocol articles, which describe protocols hosted on protocols.io. Read more information on sharing protocols at https://plos.org/protocols?utm_medium=editorial-email&utm_source=authorletters&utm_campaign=protocols.

We look forward to receiving your revised manuscript.

Kind regards,

Ghadeer A.R.Y. Suaifan

Academic Editor

PLOS One

Journal Requirements:

When submitting your revision, we need you to address these additional requirements.

1.Please ensure that your manuscript meets PLOS ONE's style requirements, including those for file naming. The PLOS ONE style templates can be found at

https://journals.plos.org/plosone/s/file?id=wjVg/PLOSOne_formatting_sample_main_body.pdf and

https://journals.plos.org/plosone/s/file?id=ba62/PLOSOne_formatting_sample_title_authors_affiliations.pdf

2. We note that your Data Availability Statement is currently as follows: All relevant data are within the manuscript and in Supporting Information files.

Please confirm at this time whether or not your submission contains all raw data required to replicate the results of your study. Authors must share the “minimal data set” for their submission. PLOS defines the minimal data set to consist of the data required to replicate all study findings reported in the article, as well as related metadata and methods (https://journals.plos.org/plosone/s/data-availability#loc-minimal-data-set-definition).

For example, authors should submit the following data:

- The values behind the means, standard deviations and other measures reported;

- The values used to build graphs;

- The points extracted from images for analysis.

Authors do not need to submit their entire data set if only a portion of the data was used in the reported study.

If your submission does not contain these data, please either upload them as Supporting Information files or deposit them to a stable, public repository and provide us with the relevant URLs, DOIs, or accession numbers. For a list of recommended repositories, please see https://journals.plos.org/plosone/s/recommended-repositories.

If there are ethical or legal restrictions on sharing a de-identified data set, please explain them in detail (e.g., data contain potentially sensitive information, data are owned by a third-party organization, etc.) and who has imposed them (e.g., an ethics committee). Please also provide contact information for a data access committee, ethics committee, or other institutional body to which data requests may be sent. If data are owned by a third party, please indicate how others may request data access.

3. When completing the data availability statement of the submission form, you indicated that you will make your data available on acceptance. We strongly recommend all authors decide on a data sharing plan before acceptance, as the process can be lengthy and hold up publication timelines. Please note that, though access restrictions are acceptable now, your entire data will need to be made freely accessible if your manuscript is accepted for publication. This policy applies to all data except where public deposition would breach compliance with the protocol approved by your research ethics board. If you are unable to adhere to our open data policy, please kindly revise your statement to explain your reasoning and we will seek the editor's input on an exemption. Please be assured that, once you have provided your new statement, the assessment of your exemption will not hold up the peer review process.

4. PLOS ONE now requires that authors provide the original uncropped and unadjusted images underlying all blot or gel results reported in a submission’s figures or Supporting Information files. This policy and the journal’s other requirements for blot/gel reporting and figure preparation are described in detail at https://journals.plos.org/plosone/s/figures#loc-blot-and-gel-reporting-requirements and https://journals.plos.org/plosone/s/figures#loc-preparing-figures-from-image-files. When you submit your revised manuscript, please ensure that your figures adhere fully to these guidelines and provide the original underlying images for all blot or gel data reported in your submission. See the following link for instructions on providing the original image data: https://journals.plos.org/plosone/s/figures#loc-original-images-for-blots-and-gels.

In your cover letter, please note whether your blot/gel image data are in Supporting Information or posted at a public data repository, provide the repository URL if relevant, and provide specific details as to which raw blot/gel images, if any, are not available. Email us at plosone@plos.org if you have any questions.

5. If the reviewer comments include a recommendation to cite specific previously published works, please review and evaluate these publications to determine whether they are relevant and should be cited. There is no requirement to cite these works unless the editor has indicated otherwise.

[Note: HTML markup is below. Please do not edit.]

Reviewers' comments:

Reviewer's Responses to Questions

**Comments to the Author**

1. Is the manuscript technically sound, and do the data support the conclusions?

The manuscript must describe a technically sound piece of scientific research with data that supports the conclusions. Experiments must have been conducted rigorously, with appropriate controls, replication, and sample sizes. The conclusions must be drawn appropriately based on the data presented.

Reviewer #1: Partly

Reviewer #2: Yes

**********

2. Has the statistical analysis been performed appropriately and rigorously?

Reviewer #1: Yes

Reviewer #2: No

**********

3. Have the authors made all data underlying the findings in their manuscript fully available?

The PLOS Data policy requires authors to make all data underlying the findings described in their manuscript fully available without restriction, with rare exception (please refer to the Data Availability Statement in the manuscript PDF file). The data should be provided as part of the manuscript or its supporting information, or deposited to a public repository. For example, in addition to summary statistics, the data points behind means, medians and variance measures should be available. If there are restrictions on publicly sharing data—e.g. participant privacy or use of data from a third party—those must be specified.

Reviewer #1: Yes

Reviewer #2: Yes

**********

4. Is the manuscript presented in an intelligible fashion and written in standard English?

PLOS ONE does not copyedit accepted manuscripts, so the language in submitted articles must be clear, correct, and unambiguous. Any typographical or grammatical errors should be corrected at revision, so please note any specific errors here.

Reviewer #1: No

Reviewer #2: No

**********

5. Review Comments to the Author

Please use the space provided to explain your answers to the questions above. You may also include additional comments for the author, including concerns about dual publication, research ethics, or publication ethics. (Please upload your review as an attachment if it exceeds 20,000 characters)

Reviewer #1: 1. The consent form that was supposed to be written was not part of the submission, even though it was mentioned in the manuscript.

2. The English language needs substantial revision. Pseudomonas aeruginosa must consistently be typed in Italic font.

3. The classification of woutnd infection severity in the methodology section requires further clarification. What criteria were used to classify the severity, including the count and percentage?

4. Table 3 presents the findings, however, the column headings do not describe the significance of the numbers presented, specifically which number indicates the sample size and which represents the % ?

5. What is the significance of (*) in the P-value column of table 3?

6. What is the significance of the PCR amplification in this study? No discussion of the findings in the context of the manuscript.

7. The discussion should not reiterate the results, it should analyze and interret the findings in relation to the existing published data in the literature.

8. The conclusion should recommend measures to be taken to address the problems raised in the research questions.

9. What are the limitations associated with the current study?

Reviewer #2: I. Summary of the Research

This manuscript investigates the prevalence, associated risk factors, and antimicrobial susceptibility patterns of Pseudomonas aeruginosa in diabetic wound infections in Duhok Province, Iraq. A notable strength of this work is its departure from the standard focus on diabetic foot ulcers (DFUs) to include various anatomical sites, which provides a more comprehensive understanding of the pathogen's impact. The study utilizes a multi-pronged identification approach, including molecular confirmation via 16S rDNA PCR, which enhances the analytical validity of the findings.

However, the manuscript contains a critical temporal error and several major methodological and statistical deficiencies that must be addressed to ensure scientific integrity and reproducibility.

II. Major Revisions (Critical)

1. Fundamental Temporal Inconsistency

Issue: The study period is reported as being in the future (“July 2024 to July 2025”) in the Abstract (Line 35) and the Methods (Line 112). This discrepancy suggests the results are hypothetical or simulated, which severely undermines the credibility of the entire paper.

Recommendation: Immediately correct the dates to reflect the actual, past timeframe of data collection. This is a non-negotiable requirement for truthful reporting.

2. Statistical Rigor and Multivariable Analysis

Issue: The statistical analysis is underdeveloped, appearing only in Lines 187. The authors list t-tests and Chi-square tests but omit any multivariable analysis (e.g., binary logistic regression). Without controlling for confounders, the claims regarding "associated risk factors" are not robustly supported.

Recommendation: Conduct a multivariable analysis to identify independent risk factors and report adjusted odds ratios (AORs) with 95% confidence intervals (CIs).

3. Sample Size Justification

Issue: The manuscript mentions the sample size of 141 in Line 113, but provides no justification for this number. Without a power analysis, it is unclear if the study was adequately powered to detect significant associations, such as those for BMI and wound site.

Recommendation: Provide a detailed sample size calculation or a retrospective power analysis in the Methods section.

4. Data Availability and Transparency

Issue: The statement that “All relevant data are within the manuscript and its Supporting Information files” is misleading because there are no Supporting Information files included in the submission.

Recommendation: Upload the anonymized dataset as per the journal’s format (e.g. as Supplementary Files).

III. Specific Comments & Technical Points

Results and Logical Consistency:

- BMI Misinterpretation: The text in Line 209 (Results) and Line 325 (Discussion) incorrectly implies that obese individuals had the highest "rate" of P. aeruginosa infection. However, Table 2 (Line 233) clearly shows Normal weight individuals had the highest rate (58.33%) within their group. Revise the narrative to match the data.

- Unsupported Conclusions: The conclusion in Line 352 states that the highest rates were among the "uneducated". This contradicts Line 217, which explicitly states the association with educational level was not statistically significant (P = 0.2933).

- Clarity and Style

Redundancy: Remove the redundant phrasing ("widespread epidemic" repeated) in Line 64.

Incomplete Sentence: Correct the truncated sentence regarding ECDC data in Line 96.

Standardization: Ensure "P. aeruginosa" is formatted consistently throughout.

Sentence Structure: Simplify the convoluted description of wound locations in Line 246 and treatment types in Line 265.

- Reproducibility Details:

Sampling: Explicitly state if consecutive, random, or convenience sampling was used in Line 112.

CLSI version: Specify the exact version/year of the CLSI guidelines used in Line 149.

PCR Master Mix: Provide the manufacturer and catalog number for the master mix in Line 164.

The entire manuscript needs major proofreading for language and grammar.

**********

6. PLOS authors have the option to publish the peer review history of their article (what does this mean?). If published, this will include your full peer review and any attached files.

If you choose “no”, your identity will remain anonymous but your review may still be made public.

**Do you want your identity to be public for this peer review?**  For information about this choice, including consent withdrawal, please see our Privacy Policy.

Reviewer #1: No

Reviewer #2: No

**********

[NOTE: If reviewer comments were submitted as an attachment file, they will be attached to this email and accessible via the submission site. Please log into your account, locate the manuscript record, and check for the action link "View Attachments". If this link does not appear, there are no attachment files.]

To ensure your figures meet our technical requirements, please review our figure guidelines: https://journals.plos.org/plosone/s/figures

You may also use PLOS’s free figure tool, NAAS, to help you prepare publication quality figures: https://journals.plos.org/plosone/s/figures#loc-tools-for-figure-preparation.

NAAS will assess whether your figures meet our technical requirements by comparing each figure against our figure specifications.

# Author comment: Author response to Decision Letter 1

**DOI:** 10.1371/journal.pone.0354256.r002

29 Apr 2026

Dear Editor,

We are pleased to submit the revised version of our manuscript entitled “Prevalence and Associated Risk Factors of Pseudomonas aeruginosa Infection in Diabetic Wounds in Duhok Province, Iraq.”

We would like to thank the reviewers and editorial team for their valuable comments and constructive suggestions, which have significantly improved the quality of our manuscript. We have carefully addressed all comments, and all revisions and changes have been highlighted in red color in the revised manuscript for ease of review.

We hope that the revised version meets the journal’s requirements and is suitable for publication.

Sincerely,

Dr Ibrahim Naqid

Reviewer #1:

1. The consent form that was supposed to be written was not part of the submission, even though it was mentioned in the manuscript.

Thank you for your comment. We apologize for the oversight regarding the consent form. The written informed consent, which was mentioned in the manuscript, has now been uploaded to the submission system as required.

2. The English language needs substantial revision. Pseudomonas aeruginosa must consistently be typed in Italic font.

Thank you for your comment. We have thoroughly revised the manuscript to improve the English language quality throughout the text. In addition, Pseudomonas aeruginosa has been consistently formatted in italic font in all instances, as required.

3. The classification of woutnd infection severity in the methodology section requires further clarification. What criteria were used to classify the severity, including the count and percentage?

Thank you for your comment. We have clarified the classification of wound infection severity in the methodology section. The severity was defined based on the degree of tissue involvement and clinical presentation, including ulcers (necrosis with erythema, pain, and discharge), abscess formation (pus collection), cellular necrosis (tissue death due to hypoxia or vascular occlusion), and gangrene (advanced necrosis with or without systemic symptoms). The count and percentage of each category have been clearly reported in Table 3.

4. Table 3 presents the findings, however, the column headings do not describe the significance of the numbers presented, specifically which number indicates the sample size and which represents the % ?

Thank you for your comment. We have revised Table 3 to improve clarity. The column headings have been updated to clearly distinguish the sample size (n) and percentage (%), ensuring that each value is correctly identified and easily interpreted.

5. What is the significance of (*) in the P-value column of table 3?

Thank you for your comment. The asterisk () in the P-value column of Table 3 indicates a statistically significant result (p < 0.05). This clarification has now been added in the footnote of Table 3 as “ = statistically significant difference” to ensure clear interpretation.

6. What is the significance of the PCR amplification in this study? No discussion of the findings in the context of the manuscript

Thank you for your comment. The required changes added in the first paragraph of discussion. PCR amplification of the 16S rDNA gene was used in this study to confirm the identification of Pseudomonas aeruginosa with high specificity and accuracy. This molecular method strengthens the reliability of the conventional biochemical and Vitek®2 identification results by reducing the possibility of misidentification. Therefore, PCR confirmation supports the validity of the reported prevalence and ensures accurate detection of P. aeruginosa in diabetic wound infections.

7. The discussion should not reiterate the results; it should analyze and interret the findings in relation to the existing published data in the literature.

Thank you for your comment. We have revised the Discussion section by removing repeated results and ensuring that it focuses on interpretation and comparison of findings with relevant published literature. All redundant result descriptions have been deleted and the section has been restructured to improve analytical depth and alignment with standard discussion format.

8. The conclusion should recommend measures to be taken to address the problems raised in the research questions.

Thanks. The required changes have been added in the conclusion section. Pseudomonas aeruginosa is a significant pathogen in diabetic wound infections with high multidrug resistance, while most sociodemographic and clinical factors showed no strong independent association. The findings highlight the importance of early detection and proper management of diabetic wounds, particularly in high-risk anatomical sites such as the leg region. Strengthening infection control practices, promoting rational antibiotic use, and improving patient education on wound care are essential to reduce complications and antimicrobial resistance in diabetic patients.

9. What are the limitations associated with the current study?

Thank you for your comment. We have addressed this point by clearly stating the study limitations in the Discussion section. These include the cross-sectional design, which limits causal inference, the single-province setting, and the relatively small sample size, which may affect generalizability.

9. Reviewer #2: I. Summary of the Research

This manuscript investigates the prevalence, associated risk factors, and antimicrobial susceptibility patterns of Pseudomonas aeruginosa in diabetic wound infections in Duhok Province, Iraq. A notable strength of this work is its departure from the standard focus on diabetic foot ulcers (DFUs) to include various anatomical sites, which provides a more comprehensive understanding of the pathogen's impact. The study utilizes a multi-pronged identification approach, including molecular confirmation via 16S rDNA PCR, which enhances the analytical validity of the findings.

Thank you for your thoughtful and constructive evaluation of our manuscript. We appreciate your recognition of the study’s broader scope beyond diabetic foot ulcers, as well as the inclusion of multiple anatomical sites to provide a more comprehensive understanding of Pseudomonas aeruginosa infections in diabetic wounds.

Your positive feedback is highly valued and encourages us to further refine and strengthen our work.

However, the manuscript contains a critical temporal error and several major methodological and statistical deficiencies that must be addressed to ensure scientific integrity and reproducibility.

II. Major Revisions (Critical)

1. Fundamental Temporal Inconsistency

Issue: The study period is reported as being in the future (“July 2024 to July 2025”) in the Abstract (Line 35) and the Methods (Line 112). This discrepancy suggests the results are hypothetical or simulated, which severely undermines the credibility of the entire paper.

Recommendation: Immediately correct the dates to reflect the actual, past timeframe of data collection. This is a non-negotiable requirement for truthful reporting.

Thank you for this important comment. We apologize for the error in reporting the study period. The dates have been corrected to reflect the actual data collection timeframe, and all inconsistencies in the Abstract and Methods sections have been revised accordingly to ensure accurate and consistent reporting throughout the manuscript.

2. Statistical Rigor and Multivariable Analysis

Issue: The statistical analysis is underdeveloped, appearing only in Lines 187. The authors list t-tests and Chi-square tests but omit any multivariable analysis (e.g., binary logistic regression). Without controlling for confounders, the claims regarding "associated risk factors" are not robustly supported.

Recommendation: Conduct a multivariable analysis to identify independent risk factors and report adjusted odds ratios (AORs) with 95% confidence intervals (CIs).

Thank you for your comment. We have performed a full reanalysis using appropriate multivariable logistic regression to control for potential confounders. The results have been updated throughout the manuscript, and adjusted odds ratios (AORs) with 95% confidence intervals (95% CIs) are now reported. In addition, the Discussion section has been revised accordingly to reflect the findings of the adjusted analysis and to ensure accurate interpretation of independent risk factors.

3. Sample Size Justification

Issue: The manuscript mentions the sample size of 141 in Line 113, but provides no justification for this number. Without a power analysis, it is unclear if the study was adequately powered to detect significant associations, such as those for BMI and wound site.

Recommendation: Provide a detailed sample size calculation or a retrospective power analysis in the Methods section.

Thank you for your valuable comment. This has been addressed and the sample size justification has been added in the Methods section. The sample size of 141 was determined based on all eligible diabetic wound infection cases admitted during the study period in selected hospitals. Therefore, a census sampling approach was used rather than a calculated sample size.

4. Data Availability and Transparency

Issue: The statement that “All relevant data are within the manuscript and its Supporting Information files” is misleading because there are no Supporting Information files included in the submission.

Recommendation: Upload the anonymized dataset as per the journal’s format (e.g. as Supplementary Files).

Thank you for your comment. We apologize for the confusion. The data availability statement has been revised accordingly, and the anonymized dataset will be uploaded as Supplementary Files in the required journal format.

III. Specific Comments & Technical Points

Results and Logical Consistency

- BMI Misinterpretation: The text in Line 209 (Results) and Line 325 (Discussion) incorrectly implies that obese individuals had the highest "rate" of P. aeruginosa infection. However, Table 2 (Line 233) clearly shows Normal weight individuals had the highest rate (58.33%) within their group. Revise the narrative to match the data.

Thank you for your careful observation, and we apologize for the confusion caused by this inconsistency. Although obesity was the most prevalent BMI category among diabetic patients in the study population, the highest proportion of Pseudomonas aeruginosa diabetic wound infections (PsA DWIs) was observed in the normal-weight group (58.33%).

- Unsupported Conclusions: The conclusion in Line 352 states that the highest rates were among the "uneducated". This contradicts Line 217, which explicitly states the association with educational level was not statistically significant (P = 0.2933).

Thank you for your careful review, and we apologize for the confusion. We have revised the conclusion accordingly to ensure consistency and accuracy

- Clarity and Style

Redundancy: Remove the redundant phrasing ("widespread epidemic" repeated) in Line 64. Thank you for your comment. The redundant phrasing (“widespread epidemic”) in Line 64 has been removed as suggested.

Standardization: Ensure "P. aeruginosa" is formatted consistently throughout.

Thank you for your comment. The formatting of P. aeruginosa has been standardized consistently throughout the manuscript.

Sentence Structure: Simplify the convoluted description of wound locations in Line 246 and treatment types in Line 265.

Thank you for your comment. The sentence structure describing wound locations and treatment types has been simplified for clarity

- Reproducibility Details:

Sampling: Explicitly state if consecutive, random, or convenience sampling was used in Line 112.

We have clarified the sampling method in the Methods section

CLSI version: Specify the exact version/year of the CLSI guidelines used in Line 149.

Thank you for your comment. The exact version/year of the Clinical and Laboratory Standards Institute (CLSI) guidelines

PCR Master Mix: Provide the manufacturer and catalog number for the master mix in Line 164.

Thank you for your comment. The manufacturer and catalog number of the PCR master mix have been added as requested

Attachment Submitted filename: Reviewer comment 25.04.2026.docx

# Peer review: Decision Letter 1

**DOI:** 10.1371/journal.pone.0354256.r003

**Authors:** Ghadeer Suaifan

7 Jul 2026

<p>Prevalence and associated risk factors of Pseudomonas aeruginosa infection in Diabetic wounds in Duhok province, Iraq

PONE-D-25-68291R1

Dear Dr. Naqid,

We’re pleased to inform you that your manuscript has been judged scientifically suitable for publication and will be formally accepted for publication once it meets all outstanding technical requirements.

Within one week, you’ll receive an e-mail detailing the required amendments. When these have been addressed, you’ll receive a formal acceptance letter and your manuscript will be scheduled for publication.

An invoice will be generated when your article is formally accepted. Please note, if your institution has a publishing partnership with PLOS and your article meets the relevant criteria, all or part of your publication costs will be covered. Please make sure your user information is up-to-date by logging into Editorial Manager at Editorial Manager® and clicking the ‘Update My Information' link at the top of the page. For questions related to billing, please contact billing support.

If your institution or institutions have a press office, please notify them about your upcoming paper to help maximize its impact. If they’ll be preparing press materials, please inform our press team as soon as possible -- no later than 48 hours after receiving the formal acceptance. Your manuscript will remain under strict press embargo until 2 pm Eastern Time on the date of publication. For more information, please contact onepress@plos.org.

Kind regards,

Ghadeer A.R.Y. Suaifan

Academic Editor

PLOS One

Additional Editor Comments (optional):

Reviewers' comments:

Reviewer's Responses to Questions

**Comments to the Author**

1. If the authors have adequately addressed your comments raised in a previous round of review and you feel that this manuscript is now acceptable for publication, you may indicate that here to bypass the “Comments to the Author” section, enter your conflict of interest statement in the “Confidential to Editor” section, and submit your "Accept" recommendation.

Reviewer #1: (No Response)

**********

2. Is the manuscript technically sound, and do the data support the conclusions?

The manuscript must describe a technically sound piece of scientific research with data that supports the conclusions. Experiments must have been conducted rigorously, with appropriate controls, replication, and sample sizes. The conclusions must be drawn appropriately based on the data presented.

Reviewer #1: Yes

**********

3. Has the statistical analysis been performed appropriately and rigorously?

Reviewer #1: Yes

**********

4. Have the authors made all data underlying the findings in their manuscript fully available?

The PLOS Data policy requires authors to make all data underlying the findings described in their manuscript fully available without restriction, with rare exception (please refer to the Data Availability Statement in the manuscript PDF file). The data should be provided as part of the manuscript or its supporting information, or deposited to a public repository. For example, in addition to summary statistics, the data points behind means, medians and variance measures should be available. If there are restrictions on publicly sharing data—e.g. participant privacy or use of data from a third party—those must be specified.

Reviewer #1: Yes

**********

5. Is the manuscript presented in an intelligible fashion and written in standard English?

PLOS ONE does not copyedit accepted manuscripts, so the language in submitted articles must be clear, correct, and unambiguous. Any typographical or grammatical errors should be corrected at revision, so please note any specific errors here.

Reviewer #1: Yes

**********

6. Review Comments to the Author

Please use the space provided to explain your answers to the questions above. You may also include additional comments for the author, including concerns about dual publication, research ethics, or publication ethics. (Please upload your review as an attachment if it exceeds 20,000 characters)

Reviewer #1: Comments to authors have been included in the attached PDF file and must be addressed prior to acceptance. Once addressed, the manuscript could be accepted for publications.

Recommendations for further research should be added at the enc.

**********

7. PLOS authors have the option to publish the peer review history of their article (what does this mean?). If published, this will include your full peer review and any attached files.

If you choose “no”, your identity will remain anonymous but your review may still be made public.

**Do you want your identity to be public for this peer review?**  For information about this choice, including consent withdrawal, please see our Privacy Policy.

Reviewer #1: No

**********

Attachment Submitted filename: PONE-D-25-68291_R1.pdf

# Editor report: Acceptance letter

**DOI:** 10.1371/journal.pone.0354256.r004

**Authors:** Ghadeer Suaifan

PONE-D-25-68291R1

PLOS One

Dear Dr. Naqid,

I'm pleased to inform you that your manuscript has been deemed suitable for publication in PLOS One. Congratulations! Your manuscript is now being handed over to our production team.

At this stage, our production department will prepare your paper for publication. This includes ensuring the following:

* All references, tables, and figures are properly cited

* All relevant supporting information is included in the manuscript submission,

* There are no issues that prevent the paper from being properly typeset

You will receive further instructions from the production team, including instructions on how to review your proof when it is ready. Please keep in mind that we are working through a large volume of accepted articles, so please give us a few days to review your paper and let you know the next and final steps.

Lastly, if your institution or institutions have a press office, please let them know about your upcoming paper now to help maximize its impact. If they'll be preparing press materials, please inform our press team within the next 48 hours. Your manuscript will remain under strict press embargo until 2 pm Eastern Time on the date of publication. For more information, please contact onepress@plos.org.

You will receive an invoice from PLOS for your publication fee after your manuscript has reached the completed accept phase. If you receive an email requesting payment before acceptance or for any other service, this may be a phishing scheme. Learn how to identify phishing emails and protect your accounts at https://explore.plos.org/phishing.

If we can help with anything else, please email us at customercare@plos.org.

Thank you for submitting your work to PLOS ONE and supporting open access.

Kind regards,

PLOS ONE Editorial Office Staff

on behalf of

Prof Ghadeer A.R.Y. Suaifan

Academic Editor

PLOS One
